# Lung cancer susceptibility from *GSTM1* deletion and air pollution with smoking status: a meta-prediction of worldwide populations

**DOI:** 10.18632/oncotarget.25693

**Published:** 2018-07-24

**Authors:** Pojui Yu, Joyce D. Kusuma, Maria Aurora R. Suarez, Shyang-Yun Pamela Koong Shiao

**Affiliations:** ^1^ Department of Nursing, Fu Jen Catholic University Hospital, New Taipei City, Taiwan (R.O.C.); ^2^ School of Nursing, College of Medicine, National Taiwan University, Taipei, Taiwan (R.O.C.); ^3^ Heritage Victor Valley Medical Group, Augusta University, Augusta, GA, USA; ^4^ Critical Care and Telemetry, Citrus Valley Health Partners, Augusta University, Augusta, GA, USA; ^5^ College of Nursing and Medical College of Georgia, Augusta University, Augusta, GA, USA

**Keywords:** Glutathione S transferase mu 1, lung cancer, meta-prediction, air pollution, smoking

## Abstract

*Glutathione S transferase mu 1* (*GSTM1*) gene has been associated with lung cancer (LC) risk, for GSTM1 enzyme playing a vital role in detoxification pathway and protective against toxic insults. The major objective of this study was to investigate *GSTM1* deletion pattern and its association with LC in the world’s population by using meta-prediction techniques. The secondary objective was to examine the effects of air pollution, smoking status, and other factors for gene-environment interactions with *GSTM1* deletion and LC risk. We completed a comprehensive search to yield a total of 170 studies (40,296 cases and 48,346 controls) published from 1999 to 2017 for meta-analyses. The results revealed that *GSTM1* deletion type was associated with increased risk of LC, while *GSTM1* present type provided protective effect for all populations combined worldwide. Subgroup analysis on the rank order of risks from highest to lowest, among racial–ethnic groups, were Chinese, South East Asian, other North Asian, European, and finally American. Additional predictive analyses presented that air pollution played a significant role with increased risks of *GSTM1* deletion and LC susceptibility, and the risks increased for smokers with higher levels of air pollution. Based on the findings of meta-predictive analysis, increased air pollution levels and smoking status presented additive effects to the LC risk susceptibilities and *GSTM1* gene polymorphisms, for gene-environment interactions. Future studies are needed to examine gene-environment interactions for *GSTM1* interacting with environmental factors and dietary interventions to mitigate the toxic effects, for LC prevention.

## INTRODUCTION

Lung cancer (LC) accounts for the second most commonly diagnosed cancer among adults and 25% of all cancer deaths, with delayed diagnosis at a late stage being associated with poor prognosis [1–4]. *Glutathione S transferase mu 1* (*GSTM1*) gene has been associated with LC risk, with GSTM1 enzyme playing a vital role in detoxification pathway and protective effect against toxic insults [2, 5–7]. GSTM1 is one of phase II detoxification enzymes that detoxify electrophilic compounds, including carcinogens, therapeutic drugs, environmental toxins, and byproducts of oxidative stress by conjugation with glutathione (GSH). *GSTM1* gene was known to be highly polymorphic and the polymorphism affects the expression of enzyme levels [5–11]. Two identified variants in *GSTM1* are a deletion and a substitution. A deletion of *GSTM1* or null mutation deactivates the enzymes, which results in the loss of function within the detoxification pathway [2–4]. *GSTM1* null genotype has been associated with increased risk of many cancers [8], and increased environmental toxins and carcinogens further increase the susceptibility of LC [2, 4, 5, 7, 12].

Environmental toxicants such as air pollution and smoking can expose lung, an organ, to oxidative stress and dis-regulate reactive oxygen species [2, 4, 5, 13–15]. Studies suggested that exposure to oxidative stress cause damage to cellular DNA that leads to mutations, genomic instability, and ultimately malignancy [2–4, 13, 14, 16]. Several studies indicated that consumption of cruciferous vegetables can reduce the risk of LC. These plants contain isothiocyanates (ITC) and indole-3-carbinol, which are known to induce phase II enzyme in the detox pathway [14, 17, 18]. ITC and indoles may inhibit the bio-activation of carcinogen from air pollution and smoke, enhance excretion of carcinogenic metabolites before it causes damage to DNA, and induce cell cycle arrest and apoptosis [18, 19]. These processes affirm the crucial role of micronutrients in the detoxification pathway for LC prevention.

To date, results from epidemiological studies on the association of *GSTM1* mutation and LC have been inconsistent and mixed with heterogeneous findings. Meta-predictive analysis can be used to address heterogeneous findings, and to cross validate the findings using various analytical methods [20]. Additional studies indicated the effects of air pollution on the association with *GSTM1* deletion. Despite these findings, previous meta-analyses did not examine the effects of gene-environment interaction, specifically air pollution and smoking status, on the association with *GSTM1* and LC risk. To fill this gap and to provide further evidence, we conducted a meta-analysis by adding meta-predictive techniques to examine the impact of exposure to air pollution on the risk of *GSTM1* deletion and LC susceptibility in various populations of the world, with subgroup analyses of LC types, smoking status, and gender status. In this meta-prediction study, we integrated the use of big-data machine-learning analytics in addition to the conventional pooled analysis, including the global maps and heat maps to visualize grouping patterns.

## RESULTS

### Characteristics of study subjects

**Figure 1:**
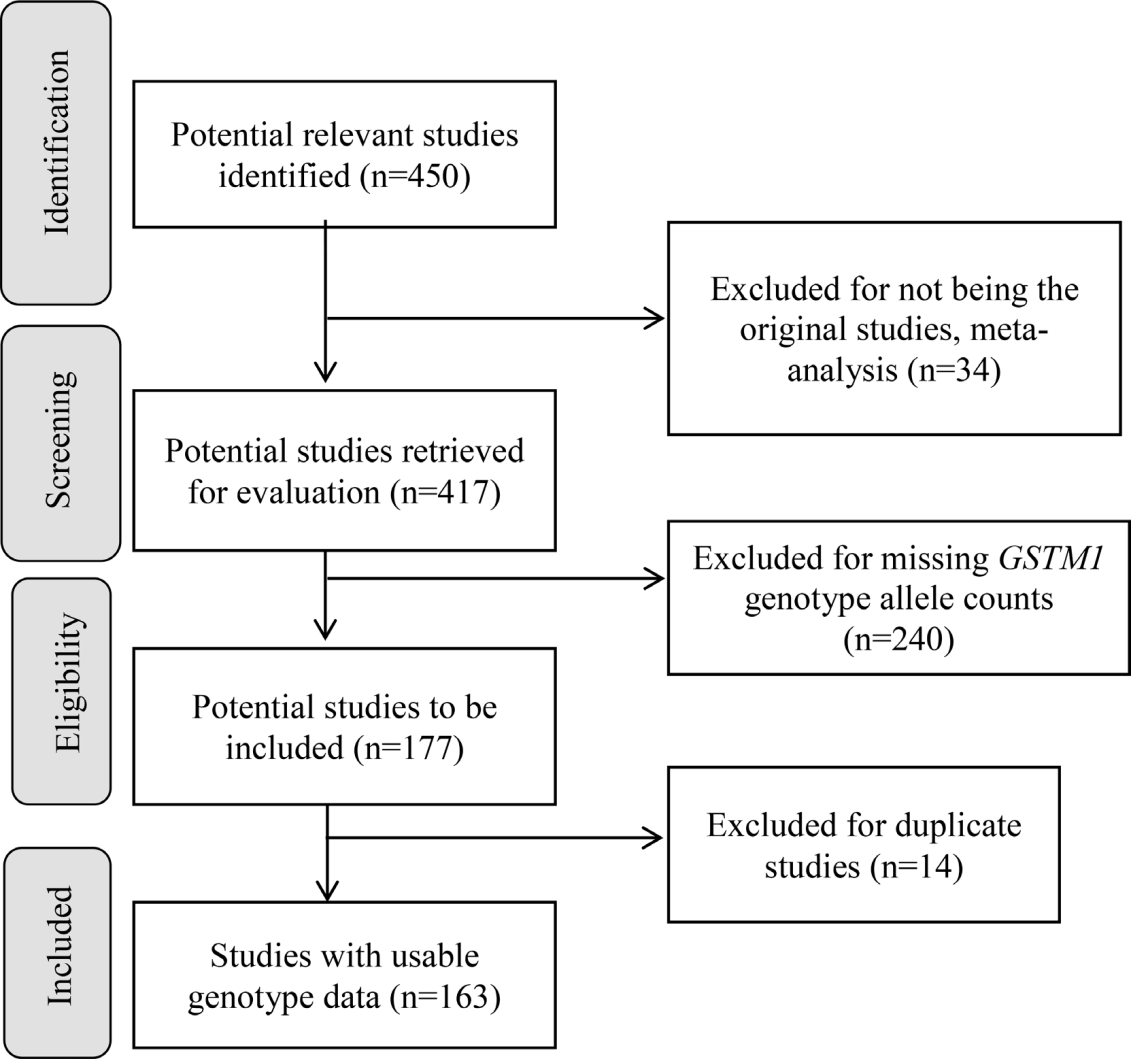
Progression on the selection of studies for the meta-analysis

**Figure 2:**
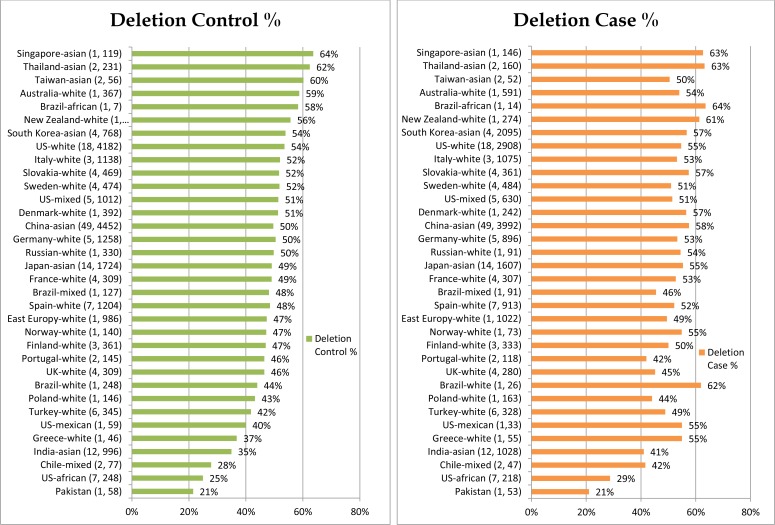
*GSTM1* % deletion per control and case groups

We have summarized how we selected studies in Figure 1. We initially identified 450 potential relevant studies published from 1999 to 2017. Through systematic screening process, we located a total of 163 papers (40,296 cases and 48,346 controls) that included data for *GSTM1* deletion. These studies were conducted in 5 continents of the world and 7 studies also included data for more than one racial-ethnic groups, yielding a total of 170 studies (see Supplementary Table 1, see Figure 2 for % *GSTM1* deletion in control and LC groups).

### Pooled analysis - by ethnic groups

For all included studies, Table 1 (summary schema) and Table 2 (detailed pooled analyses) presented increased risk of LC with *GSTM1* deletion (RR = 1.10, *p* < 0.0001), while *GSTM1* present genotype were protective against LC (RR = 0.91, *p* < 0.0001). Subgroup analysis per ethnic groups showed the rank order of highest risk of LC with *GSTM1* deletion, being among Chinese (RR = 1.20, *p* < 0.0001), South East Asian (RR = 1.12, *p* = 0.0165), other North Asian (RR = 1.08, *p* < 0.0001), European (RR = 1.06, *p* = 0.0005), and finally American (RR = 1.04, *p* = 0.02). There were no significances on the risks for the three additional ethnic subgroups of Oceanian (2 studies), African American (8 studies), and mixed ethnic groups (8 studies).

**Table 1:** Schema of significant findings on *GSTM1 deletion* and risk of lung cancer per ethnic subgroups (*n* = 170)

GSTM1 (n Case/n Control)	Deletion (n Case/n Control)	LC Type		Smoker Status^A^			Gender^B^		
		NSCLC	Mixed	Yes	No	NA	Male	Female	NA
All (40,236/48,200)									
	170 Studies (21,248/23,513) RR 1.10	38 Studies (4,231/4,132) RR 1.11	132 Studies (17,050/19,440) RR 1.10	63 Studies (7,776/6,756) RR 1.09	49 Studies (1,513/3,494) RR 1.15	104 Studies (11,508/12,430) RR 1.11	25 Studies (5,072/3,883) RR 1.08	26 Studies (2,221/3,165) RR 1.13	139 Studies (13,774/16,269) RR 1.11
European									
(13,386/17,012)	48 Studies (6,915/8,372) RR 1.06	7 Studies (497/630) NS	41 Studies (6,418/7,742) RR 1.06	18 Studies (3,766/3,400) NS	12 Studies (323/1,277) NS	28 Studies (2,444/3,108) RR 1.07	11 Studies (2,160/2,303) NS	10 Studies (555/1,058) NS	37 Studies (4,460/5,087) RR 1.08
Oceanian									
(1,541/1,101)	2 Studies (865/632) NS	1 Study (591/367)	1 Study (274/265)	1 Study (591/367)	--	1 Study (274/265)	1 Study (420/247)	1 Study (171/120)	1 Study (274/265)
American									
(5,350/7,828)	18 Studies (2,876/4,026) RR 1.04	4 Studies (798/820) NS	14 Studies (2,078/3,206) NS	8 Studies (1,094/1,329) NS	5 Studies (61/509) NS	10 Studies (1,695/2,063) NS	1 Study (35/95)	2 Studies (250/324) NS	16 Studies (2,591/3,607) NS
Mixed									
(1,537/2,510)	8 Studies (768/1,216) NS	--	8 Studies (768/1,216) NS	1 Study (136/171)	1 Study (7/74)	7 Studies (625/970) NS	1 Study (53/35)	1 Study (50/63)	7 Studies (665/1,118) NS
African American									
(790/1,024)	8 Studies (233/258) NS	1 Study (36/28)	7 Studies (197/230) NS	1 Study (33/22)	1 Study (3/2)	7 Studies (196/234) NS	--	1 study (36/28)	7 Studies (197/230) NS
Mexican									
(60/146)	1 Study (33/59)	--	1 study (33/59)	--	--	1 study (33/59)	--	--	1 study (33/59)
North Asian									
(7,432/5,888)	18 Studies (4,159/3,006) RR 1.08	5 Studies (578/774) NS	13 Studies (3,581/2,232) RR 1.09	7 Studies (685/509) RR 1.18	5 Studies (211/335) NS	10 Studies (3,232/2,088) RR 1.06	6 Studies (2,439/1,034) RR 1.08	4 Studies (621/710) RR 1.21	11 Studies (1,099/1,262) RR 1.08
Chinese									
(6,932/8,974)	49 Studies (3,992/4,452) RR 1.20	16 Studies (1,483/1,338) RR 1.17	33 Studies (2,509/3,114) RR 1.21	19 Studies (1,154/749) RR 1.27	18 Studies (712/923) RR 1.22	30 Studies (2,162/2,798) RR 1.19	3 Studies (268/340) NS	5 Studies (418/689) NS	44 Studies (3,306/3,423) RR 1.20
South East Asian									
(3,268/3,863)	18 Studies (1,407/1,492) RR 1.12	4 Studies (248/175) NS	14 Studies (1,159/1,317) NS	8 Studies (317/209) NS	7 Studies (196/374) NS	10 Studies (847/845) RR 1.20	2 Study (117/76) RR 1.57	2 Studies (120/173) RR 1.36	15 Studies (1,149/1,218) RR 1.08

*Note*: *GSTM1* = *Glutathione S transferase mu 1*; RR = relative risk; LC = lung cancer; NSCLC = non-small cell lung cancer; NA = not available; -- no data;

^A^46 studies had data for both smoker and non-smoker groups; ^B^20 studies had both male and female groups.

**Table 2:** Pooled analysis: *GSTM1* deletion and risk of lung cancer

Genotype (number of studies)	LC Case *N* = 40,296 *n* (%)	Control *N* = 48,346 *n* (%)	Test of Heterogeneity			Statistical Model	Test of Association	
			Q	*p*	I^2^ (%)		Risk Ratio (95% CI)	*p*
Deletion (170)	21,248 (52.7)	23,513 (48.6)	356.76	<0.0001	52.6%	Random	1.10 (1.08–1.13)	<0.0001
European (48)	6,915 (51.7)	8,372 (49.2)	80.47	0.002	41.6%	Random	1.06 (1.03–1.10)	0.0005
Oceanian (2)	865 (56.1)	632 (57.4)	6.47	0.01	84.5%	Random	1.00 (0.84–1.20)	0.97
American (18)	2,876 (53.8)	4,026 (51.4)	11.92	0.80	0.0%	Fixed	1.04 (1.01–1.08)	0.02
Mixed (8)	768 (50.0)	1,216 (48.4)	11.68	0.11	40.1%	Fixed	1.00 (0.94–1.07)	0.92
African American (8)	233 (29.5)	258 (25.2)	3.62	0.82	0.0%	Fixed	1.16 (0.99–1.35)	0.07
Mexican (1)	33 (55.0)	59 (40.4)	-	-	-	-	-	-
North Asian (18)	4,159(56.0)	3,006 (51.1)	19.22	0.32	11.5%	Fixed	1.08 (1.05–1.12)	<0.0001
Chinese (49)	3,992 (57.6)	4,452 (49.6)	122.14	<0.0001	60.7%	Random	1.20 (1.14–1.26)	<0.0001
South East Asian (18)	1,407 (43.1)	1,492 (38.6)	42.05	0.0007	59.6%	Random	1.12 (1.02–1.22)	0.0165
Present (170)	19,048 (47.3)	24,833 (51.4)	336.93	<0.0001	49.8%	Random	0.91 (0.89–0.93)	<0.0001
European (48)	6,471 (48.3)	8,640 (50.8)	71.96	0.01	34.7%	Random	0.95 (0.92–0.98)	0.0006
Oceanian (2)	676 (43.9)	469 (42.6)	6.22	0.01	83.9%	Random	0.99 (0.78–1.26)	0.95
American (18)	2,474 (46.2)	3,802 (48.6)	10.82	0.87	0.0%	Fixed	0.96 (0.92–0.99)	0.03
Mixed (8)	769 (50.0)	1,294 (51.6)	8.75	0.27	20.0%	Fixed	1.00 (0.94–1.06)	0.92
African American (8)	557 (70.5)	766 (74.8)	3.71	0.81	0.0%	Fixed	0.95 (0.89–1.00)	0.06
Mexican (1)	27 (45.0)	87 (59.6)	-	-	-	-	-	-
North Asian (18)	3,273(44.0)	2,882 (48.9)	19.00	0.33	10.5%	Fixed	0.91 (0.88–0.95)	<0.0001
Chinese (49)	2,940 (42.4)	4,522 (50.4)	117.76	<0.0001	59.2%	Random	0.82 (0.77–0.86)	<0.0001
South East Asian (18)	1,861 (56.9)	2,371 (61.4)	41.09	0.0009	58.6%	Random	0.93 (0.87–0.99)	0.0148
Subgroup of Deletion Type								
LC type								
NSCLC (38)	4,167 (19.6)	4,069 (17.3)	85.03	<0.0001	56.5%	Random	1.11 (1.06–1.17)	<0.0001
Mixed (132)	17,050 (80.4)	19,440 (82.7)	272.89	<0.0001	52.0%	Random	1.10 (1.07–1.13)	<0.0001
Smoker								
Yes (63)	7,776 (37.4)	6,756 (29.8)	121.19	<0.0001	49.7%	Random	1.09 (1.05–1.13)	<0.0001
No (49)	1,513 (7.3)	3,494 (15.4)	69.97	0.0209	31.4%	Random	1.15 (1.09–1.22)	<0.0001
NA (104)	11,508 (55.3)	12,430 (54.8)	243.89	<0.0001	58.2%	Random	1.11 (1.07–1.14)	<0.0001
Gender								
Male (25)	5,072 (24.1)	3,883 (16.7)	58.69	0.0003	55.7%	Random	1.08 (1.02–1.13)	0.0045
Female (26)	2,221 (10.5)	3,165 (13.6)	68.93	<0.0001	60.8%	Random	1.13 (1.06–1.22)	0.0003
NA (139)	13,774 (65.4)	16,269 (69.8)	249.95	<0.0001	46.0%	Random	1.11 (1.08–1.13)	<0.0001

Note. Data Included from 170 studies. Only 1 study was Mexican and not counted in subgroup analysis. *GSTM1* = *Glutathione S transferase mu 1*; LC = Lung Cancer; NSCLC = Non-small cell lung cancer; 46 studies had data for both smoker and non-smoker groups; 20 studies had data for both male and female groups; Q = Cochran’s Q; RR = relative risk; CI = confidence interval; NA = not available.

### Subgroup analyses by LC type per total population and ethnic groups

Per LC subtypes, Table 1 presented that the risks of LC were similar for different LC types with *GSTM1* deletion for all populations combined (non-small cell LC [NSCLC]: RR = 1.11, *p* < 0.0001; Mixed LC types: RR = 1.10, *p* < 0.0001). For ethnic subgroup analyses per LC types, the risk of LC for Chinese was slightly lower in NSCLC subtype than the mixed LC subtype (NSCLC: RR = 1.17, *p* < 0.0001; mixed LC: RR = 1.21, *p* < 0.0001) (see Supplementary Table 2). Additionally, significant risks were noted for mixed LC type in north Asian (RR = 1.09, *p* < 0.0001) and European (RR = 1.06, *p* = 0.0007).

Subgroup analyses by smoking and gender status per total population and ethnic groups

Per smoking status (Table 1), the risk of LC was mixed and presented inconsistent findings across ethnic subgroups. The risk was slightly higher for non-smokers (RR = 1.15, *p* < 0.0001) than smokers (RR = 1.09; *p* < 0.0001). However, the reversed findings were noted among Chinese (smokers: RR = 1.27, *p* < 0.0001; non-smokers: RR = 1.22, *p* < 0.0001) (see Supplementary Table 3). There were no significances for subgroup analyses of smoking status for other racial-ethnic subgroups.

The risk of LC was also mixed and presented inconsistent findings across gender subgroups (Table 1). The risk was slightly higher for female (RR = 1.13, *p* = 0.0003) than male (RR = 1.08, *p* = 0.0045). Similar risks of LC are noted in other North Asian (female: RR = 1.21, *p* = 0.0403; male: RR = 1.08, *p* = 0.0039). However, the reversed findings were noted among South East Asian male when compared to female (male: RR = 1.57, *p* = 0.0004; female: RR = 1.36, *p* = 0.0006) (see Supplementary Table 4). No significant findings were present for subgroup analyses on gender status of other racial ethnic groups.

### Subgroup analyses by countries

To identify sources of heterogeneity, we further performed subgroup analyses per countries using geographic information system (GIS) to visualize regional distributions and to validate the heterogeneity of the findings. Countries were divided based on geographical area. These geographical analyses showed the rank order of highest risk of LC with *GSTM1* deletion, being Chinese, South East Asian, other North Asian, European countries and American countries (Table 2, Supplementary Figure 1A–1D). The global maps demonstrated the variations in the *GSTM1* deletion and their LC risk susceptibilities across regions. In the first two GIS maps, we used the continuous color spectrum from yellow to red, representing the increasing levels of polymorphisms, and in the third map, red-green colors – red indicating LC risk, and green indicating protective effects. Similar to the pooled meta-analysis, GIS maps showed that *GSTM1* deletion played a risk role in LC in most countries except Australia, Pakistan, Poland, Sweden, Italy, United Kingdom (UK) and Portugal (Supplementary Figure 2).

### Meta-prediction

Given the heterogeneous findings on the effects of *GSTM1* deletion and the risk susceptibility of LC, we performed meta-predictive analysis using both big-data machine-learning predictive analytics and conventional analyses (Table 3). We used both partition tree and Tukey’s tests to examine the potential interaction between air pollution and deletions, and their impact on LC risks. Based on the guidelines from the World Health Organization on air pollution, we used the levels of death from air pollution (APD) as the measure of air quality (Level 2: 51–100, Level 3: 101–250, and Level 4: > 251 deaths/million) [33–38]. The partition tree and Tukey’s test results converged and showed significant differences between APD Levels 3 and 4 (*p* < 0.0001), and between Levels 2 and 4 (*p* = 0.0056) for percent *GSTM1* deletion by APD for LC cases. The same trend of statistical significance was noted on *GSTM1* present type for LC cases. Furthermore, on the risk for *GSTM1* deletion, significant differences were identified between Levels 3 and 4 (*p* = 0.0479), with the smallest AICc of -24.28. There were no significant differences based on gender status. To further illustrate the significance, we plotted those results on nonlinear curves. We noticed increased percentages of *GSTM1* deletion for all groups of LC (Supplementary Figure 3A), NSCLC (Supplementary Figure 3B) and mixed LC type (Supplementary Figure 3C); and non-smoker groups (Supplementary Figure 3D) with the increased air pollution (Level 2: < 100, Level 3: 101–250, and Level 4: > 251 deaths/million). In contrast, the increase in deletion rates per air pollution levels were not as noticeable for the control groups. The results on the heat map were revealing for data density with the red blocks being the areas of high data concentration and the nonlinear fit line following the dense data (the red cells) for the percentages of *GSTM1* deletion (Supplementary Figure 4A–4H) and LC risk (Supplementary Figure 5A–5D).

**Table 3:** Meta-prediction: Death from air pollution (APD) on *GSTM1* for control (Ct) and lung cancer (LC) cases, and LC risks

Partition tree						Tukey’s test					
Variable	AlCc	APD	Count	Mean	SD	Levels compared	Difference	SE Difference	Lower CI	Upper CI	*p*
Ct Deletion	1255.60	2&3 4	89 81	45.49 48.65	10.52 8.47	4/3 4/2 2/3	3.2217 3.0779 0.1438	1.6917 1.9478 2.0896	–0.7790 –1.5285 –4.7978	7.2224 7.6842 5.0854	0.1406 0.2571 0.9974
LC Deletion	1241.31	2&3 4	89 81	49.14 55.97	9.99 8.25	4/3 4/2 2/3	7.4552 5.8628 1.5923	1.6190 1.8642 1.9998	3.6264 1.4544 –3.1370	11.2839 10.2713 6.3216	<0.0001 0.0056 0.7059
RR Deletion	-24.28	2&3 4	89 81	1.1007 1.1826	0.1777 0.2632	4/3 4/2 2/3	0.0933 0.0642 0.0291	0.0391 0.0451 0.0484	–0.0007 –0.0424 –0.0853	0.1860 0.1709 0.1435	0.0479 0.3310 0.8197
LC Subgroups - NSCLC											
Ct Deletion	284.38	2&3 4	15 23	44.85 48.66	12.66 6.99	4/3 4/2 2/3	5.8817 2.4280 3.4537	4.4341 3.8031 5.0979	–4.9697 –6.8792 –9.0223	16.7332 11.7352 15.9298	0.3903 0.8000 0.7780
LC Deletion	276.45	2&3 4	15 23	49.21 54.62	10.80 6.94	4/3 4/2 2/3	8.3600 3.4568 4.9032	3.9557 3.3928 4.5479	–1.3207 –4.8462 –6.2269	18.0407 11.7599 16.0332	0.1017 0.5701 0.5337
RR Deletion	-5.90	2 3&4	9 29	1.15 1.14	0.29 0.18	2/3 2/4 4/3	0.0341 0.0150 0.0191	0.1125 0.0839 0.0977	–0.2413 –0.1904 –0.2204	0.3095 0.2204 0.2586	0.9507 0.9826 0.9792
*Mixed LC* Ct Deletion	977.85	2&3 4	74 58	45.61 48.65	10.13 9.05	4/3 4/2 3/2	2.8863 3.3022 0.4160	1.8943 2.2913 2.3640	–1.6054 –2.1306 –5.1893	7.3779 8.7351 6.0213	0.2833 0.3229 0.9831
LC Deletion	970.32	2&3 4	74 58	49.12 56.50	9.90 8.71	4/3 4/2 2/3	7.7055 6.7632 0.9423	1.8401 2.2257 2.2963	3.3424 1.4859 –4.5025	12.0685 12.0405 6.3871	0.0002 0.0080 0.9114
RR Deletion	-14.01	2&3 4	74 58	1.09 1.20	0.16 0.29	4/3 4/2 2/3	0.1142 0.0915 0.0227	0.0442 0.0535 0.0552	0.0094 –0.0353 –0.1081	0.2191 0.2183 0.1536	0.0291 0.2050 0.9107
*Smoker* Ct Deletion	470.38	2 3&4	14 49	44.03 46.76	9.56 9.83	4/3 4/2 3/2	0.1909 2.8077 2.6168	2.8888 3.1891 3.4704	–6.7516 –4.8564 –5.7234	7.1334 10.4717 10.9569	0.9976 0.6547 0.7324
LC Deletion	462.77	2&3 4	33 30	45.65 56.35	10.91 6.84	4/3 4/2 3/2	7.8321 14.5944 6.7624	2.6206 2.8930 3.1482	1.5341 7.6419 –0.8035	14.1300 21.5470 14.3282	0.0112 <0.0001 0.0888
RR Deletion	3.27	2&3 4	33 30	1.01 1.25	0.14 0.32	4/3 4/2 3/2	0.1938 0.2849 0.0910	0.0702 0.0775 0.0844	0.0250 0.0985 –0.1117	0.3626 0.4712 0.2938	0.0206 0.0015 0.5308
*Non-smoker* Ct Deletion	388.52	2&3 4	22 27	42.07 48.32	13.86 10.63	4/3 4/2 2/3	6.5741 5.7677 0.8064	4.1549 4.7373 5.3371	–3.4884 –5.7054 –12.1191	16.6366 17.2407 13.7320	0.2635 0.4491 0.9875
LC Deletion	401.24	2 3&4	9 40	44.29 56.05	10.27 14.50	4/3 4/2 3/2	4.3969 13.1857 8.7889	4.6874 5.3445 6.0211	–6.9552 0.2423 –5.7932	15.7490 26.1291 23.3709	0.6193 0.0450 0.3196
RR Deletion	99.91	2 3&4	9 40	1.08 1.31	0.24 0.70	4/2 3/4 3/2	0.1391 0.2993 0.4383	0.2441 0.2141 0.2750	–0.4522 –0.2193 –0.2278	0.7303 0.8178 1.1044	0.8369 0.3505 0.2586

*Note*. AICc = Alkaike’s information criterion; APD = Death rates from air pollution levels per million (2: ≤*100*, 3: *101–250*, 4: ≥ *251*); RR = risk ratio; *GSTM1 = Glutathione S transferase mu 1*; CI = confidence interval.

**Figure 3:**
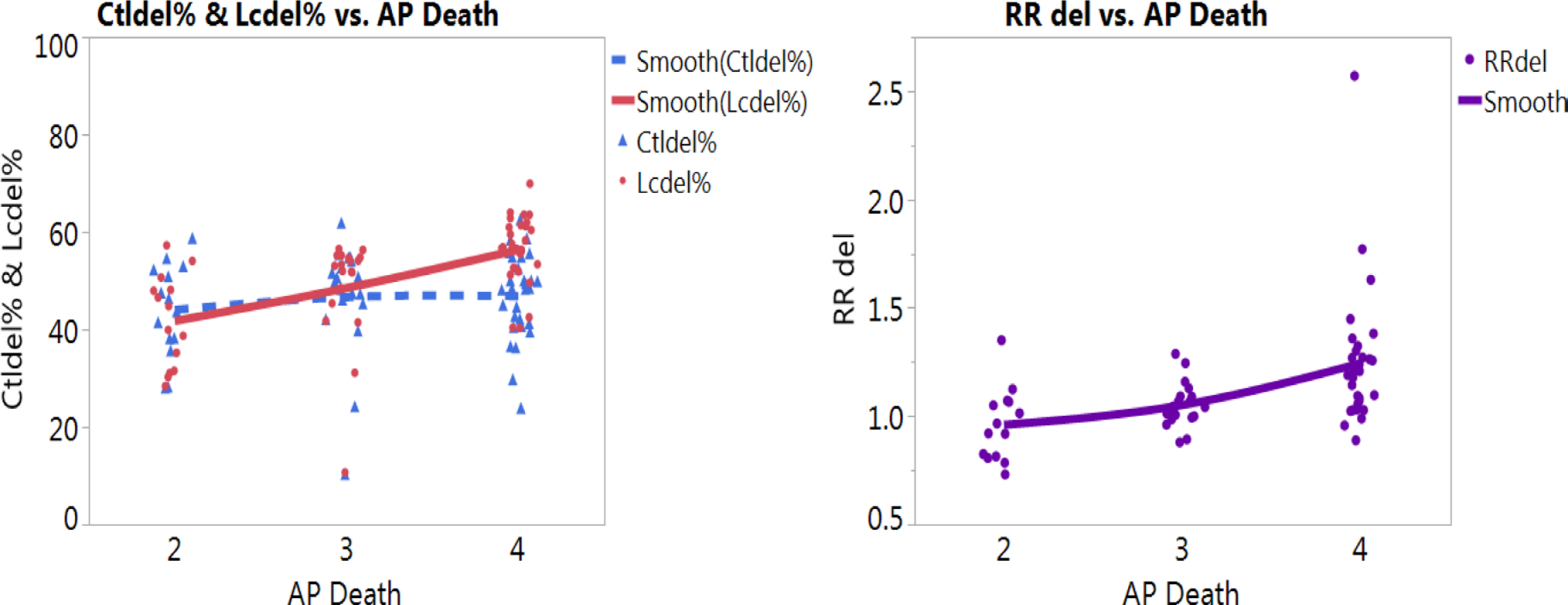
Nonlinear fit on percentages of *GSTM1* deletion per control (blue color) and lung cancer (LC, red color) groups (left graph) and LC risk (right graph) with death from air pollution in smokers (AP death: Death from air pollution, Levels per million: 2: < 100, 3:101–250, 4: > 251)

**Figure 4:**
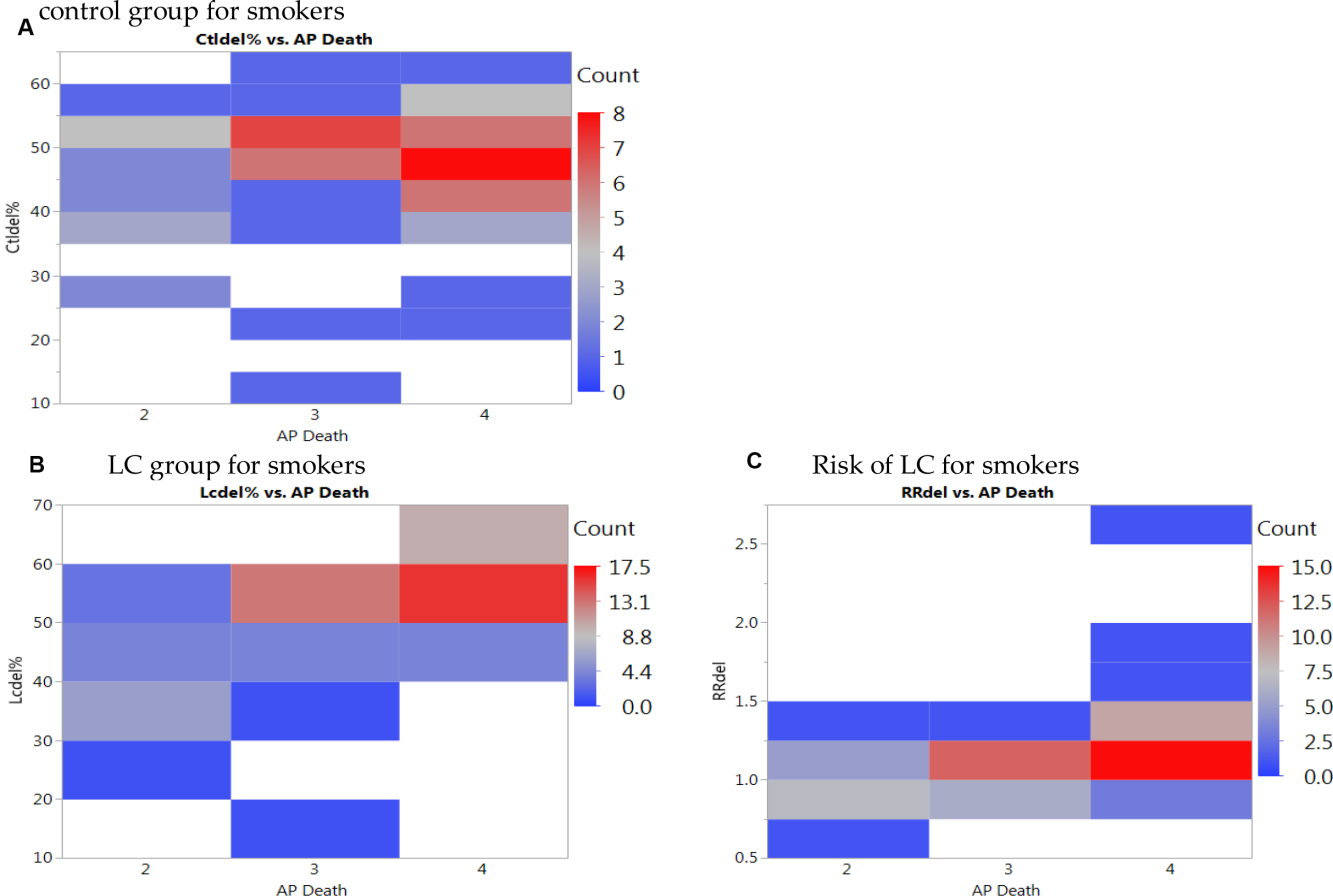
Heat maps of *GSTM1* deletion for smokers per (**A**) control group, (**B**) lung cancer (LC) group, and (**C**) LC risk for smokers with death from air pollution (Levels per million: 2: < 100, 3:101–250, 4: > 251).

Higher percentages of *GSTM1* deletion was also noted with the smoking status for smokers (Figure 3, left graph). We noticed obvious increased risks of LC for smokers with the increased air pollution from low levels (Level 2 and Level 3) to high level (Level 4) (Figure 3 right graph). Similar trends, however, no obvious increases of LC risks were noted for non-smokers or other LC types with *GSTM1* deletion (Supplementary Figure 3A–3D). The most noteworthy finding is that with the increased air pollution levels, in smokers, the LC risk (Figure 3, right graph) was significantly higher in Level 4 (RR = 1.25) than other two levels (RR = 1.01) (*p* < 0.05 for both Tukey’s tests between Level 4 versus Levels 3 and 2), based on *GSTM1* deletion. These significantly increased LC risks for smokers at higher air pollution, contrary to not noticeable increases for other subgroups (Supplementary Figure 3A–3D), presented additive effects of gene-environment interactions based on *GSTM1* deletion interacting with air pollution and smoking status. The results on the heat map were revealing for data density with the red blocks being the areas of high data concentration and the nonlinear fit line following the dense data (the red cells) for the percentages of *GSTM1* deletion (Figure 4A, 4B) and LC risk (Figure 4C).

## DISCUSSION

To date, previous studies presented the combined effects of GST family on the LC risks [21–26]. By using the meta-predictive analyses, we provided the most inclusive analyses of LC risk susceptibility based on *GSTM1* deletion interacting with air pollution and smoking status. We completed a comprehensive search to yield a total of 170 studies (40,296 cases and 48,346 controls) published from 1999 to 2017. The analyses by countries indicated increased *GSTM1* deletion rates and LC risks in Asian countries. Subgroup analysis on the rank order of risks from highest to lowest, among racial–ethnic groups, were Chinese, South East Asian, other North Asian, European, and finally American. These studies were conducted around the globe and its continents (e.g., Australia, Europe, North and South America, and Asia). The most investigated racial-ethnic populations for *GSTM1* in association with LC was Asian (85 studies), Caucasian (68 studies), African (8 studies), Mexican (1 study), and mixed racial-ethnic groups (8 studies). Our results confirmed previous meta-analyses that *GSTM1* deletion was associated with increased risk of LC [27–32], while *GSTM1* present genotype provided protective effect.

Additional noteworthy findings from subgroup analyses showed that higher risk of LC was presented among non-smokers than smokers with *GSTM1* deletion in worldwide populations combined. Conversely, smokers had higher risks of LC than non-smokers of LC with *GSTM1* deletion in Chinese subgroup. The findings about non-smokers having overall higher risk of LC than smokers with *GSTM1* deletion in our findings are consistent with a previous meta-analysis of Chinese populations [2]. However, we used risk ratios to standardize these risks (with the total counts as the denominator instead of one of the deletion or present type as the denominator) as contrary to the use of odds ratios (using one of the deletion or present type as the denominator) in previous meta-analyses. The standardized ratio is necessary when conducting gene-environment interactions across various factors for their standardized effects on the outcomes of polymorphism or LC risk [20, 33, 34]. The mechanism of higher LC risks from tobacco included inhibiting GST detoxification pathway and on the phase 1 metabolism of cytochrome *P4501A* promoting the carcinogenic effect and limiting the detoxification property of *GSTM1* [35, 36]. Furthermore, the high prevalence of air pollution in China and in other countries may have more impact on the results for smoking and LC risk [3, 35–40]. Future studies can continue to use the standardized risk ratios to see the differences on the risks across different subtypes.

In the gender subgroup analyses, in Southeast Asian subgroup, male gender had higher risk of LC than female gender. For two Southeast Asian studies, male patients had the history of cigarette smoking, tobacco chewing and drinking alcohol [35, 36] with more smokers in the LC group (66%) than the control group (37%). Possible additional explanation on difference of risk between gender may lay in dietary intake, that quercetin-rich foods taken in South Asians could reduce the risk of LC through overall upregulation of *GSTM1*, especially for smokers [19, 39]. Individual studies noted increased *GSTM1* deletion in squamous cell carcinoma (SCC) than other LC subtypes [37, 38], in younger and female LC patients [39], and in smokers [2, 40]. A previous meta-analysis for Chinese populations presented higher risks of LC with *GSTM1* deletion for SCC and adenocarcinoma (AC) than the small cell (SC) LC types [2]. A second meta-analysis in Chinese population also reported association of SCC and SC LC than AC subtypes being associated with smoking history [28]. No previous meta-analysis studies reported interaction structure or nested structure of LC subtypes with smoking and gender status, rather all studies reported grouping strata of these factors with *GSTM1* without interaction structure. For LC subtypes, we found similar risks for NSCLC and mixed LC subgroups, with the strata of smoking status according to the data presented in the original studies. Future studies are needed to report *GSTM1* deletion with interactions of LC subtypes nested with the smoking and gender strata.

Our findings illustrated the complexity of gene-environment interactions with smoking status across regions and ethnic groups. As the studies on ITC and indole from crucifer-vegetable consumptions showed critical role of micronutrients in detoxification pathway and LC prevention [14, 17–19], studies are needed to further identify ways to decrease LC risks in population studies. Specifically, future studies are needed to examine how diet, environmental factors including air pollution and smoking status interact with *GSTM1* deletion and polymorphisms across different regions and ethnic groups to prevent LC. Dietary management can be further examined in future intervention studies associating gene-environment interactions for LC prevention. Additionally, future research can be designed to examine other factors for gene environment interactions, such as ITC vegetable consumptions, smoking status, and other risk factors in association with gene-environment interactions for LC prevention.

Using meta-predictive techniques, we further presented the potential impact of air pollution on increased *GSTM1* deletion rates and LC risks. Air pollution played a significant role with increased *GSTM1* deletion and LC susceptibility for smokers. In countries with high levels of air pollution (Level 4), for smokers, *GSTM1* deletion was a risk to LC susceptibility for most countries except Turkey. Among countries with lower levels of air pollution (Level 2), for smokers, *GSTM1* deletion was a risk in Finland and India. From the risk analyses, we found that smoking and increased air pollution had additive effects to the LC risk susceptibilities in addition to the effects of *GSTM1* gene polymorphisms on LC risks, for gene-environment interactions (Figure 2).

This meta-analysis should be interpreted within the context of its potential limitations. Limitations of the study include that this study is a population-based study. While we added the effects of air pollution and smoking as possible important contributors of *GSTM1* deletion and LC risks, this study is not a study to examine the mechanisms to delineate the interaction effects of air pollution and smoking on *GTSM1* deletion and LC. From our meta-prediction analysis, we found that air pollution is the most influential factor but not gender and other factors for their effects on *GSTM1* polymorphism and LC risks interacting with gene polymorphisms [41–45]. As none of the original individual studies reported the *GSTM1* deletion within the interaction or nested contexts of LC subtypes with smoking, we were unable to delineate additional interaction effects for other factors such as gender status with the current meta-analysis data layout. Future studies are needed to accumulate common data elements for the important factors in addition to the gene polymorphisms with a data repository that enables the examination of gene-environment interactions of *GSTM1* with various LC subtypes with new emerging interaction analytics [46, 47].

## MATERIALS AND METHODS

### Characteristics of original studies

A literature search was conducted using PubMed database for human studies on LC and *GSTM1*. The database was periodically searched for latest articles over the course of investigation till 2017, until no additional eligible studies were identified. Additionally, previous meta-analysis and review papers were used to cross reference and trace back to all original studies (See Supplementary References 1–33 following Supplementary Table 1). Of the 163 papers included, additional factors such as gender, smoking status, and types of LC were entered into the database for analysis. Seven papers have data for two racial-ethnic groups for both LC cases and controls [21–26, 48], yielding additional 7 study groups. These studies were conducted around the globe and its continents (e.g., Australia, Europe, North and South America, and Asia). Furthermore, the racial and ethnic composition of each study were checked. The most investigated racial-ethnic populations for *GSTM1* in association with LC was Asian (85 studies), Caucasian (68 studies), African (8 studies), and mixed-race groups (8 studies).

### Inclusion and exclusion criteria

The inclusion criteria were studies that 1) examined the association of *GSTM1* and LC risk, reporting the genotype allele counts for both LC cases and controls, 2) were written in English or 3) had abstract written in English with tables of genotype counts that were clearly presented. We excluded studies that 1) were written in non-English languages without genotype counts, 2) did not provide *GSTM1* genotype allele counts for LC cases and controls, and 3) were of duplicate studies. Figure 1 presents the study selection process. Of 451 identified potential relevant articles, 240 were excluded as they did not provide *GSTM1* genotype counts for LC cases and controls, and 34 were previous meta-analyses. Of the remaining 177 studies, 14 were of duplicate studies (See Supplementary References 34–47 following Supplementary Table 1). At the end, 163 papers, 7 papers with additional subgroups, with appropriate genotype counts were included in the pooled analysis (Figure 1, See Supplementary References 48–210 following Supplementary Table 1).

### Quality measures

Data extractions and entry were checked for accuracy, and systematically organized to identify possible patterns. Preliminary analysis was run to ensure that the ranges of entries and pooled results were accurate for all studies. Each study was evaluated for quality using a set of appropriate indicators adapted from multiple sources on the assessment of studies. Integrated sources for these criteria included the U.S. QUOROM consensus process on the quality of meta-analysis [49], quality reporting for observational studies [50, 51], and in recent studies using the similar analytics [45, 52]. Details on quality indicators that were used to assess the studies were presented in Supplementary Table 1. The total range of quality score was 0–30 based on three domains: 1) external validity with 10 items on demographic factors (score range of 0–11); 2) internal validity with 12 items on methods and procedures (score range of 0–12); and, 3) report quality with 7 items on study results (score range of 0–7) [52]. The total quality score of included studies ranged from 8 to 28 (out of 30 maximum score). Studies scored above 50% for the possible total score were judged to have trustworthy findings [49]. We included all studies as we did not observe differences with pooled analyses when studies with low quality scores were analyzed in separate groups for sensitivity analyses.

### Data synthesis and analysis

We entered the air-quality data for various countries. Specifically, we verified from various sources for the most current and complete air-pollution data including the death rates from air pollution (death rates per million, Level 1: < 50, Level 2: 51–100, Level 3: 101–250, Level 4: 251–400, Level 5: > 401 [53, 54]. We further verified these levels with current scales on air pollution data [55–58], and the most complete and current data on air pollution data was used for the analyses. There were no studies with Levels 1 or 5 pollution, therefore only Levels 2–4 pollution were included for final analysis.

Prior to analyses, we entered all data into Excel spreadsheets (Microsoft Corp, Redmond, WA). Hardy-Weinberg Equilibrium (HWE) analyses were checked, which was developed to assess the distribution equilibrium for the evolutionary mechanisms on the population genetics [59, 60]. Departure from the HWE with a *p* value *P <* 0.05 may be associated with factors such as population migration or stratification, and disease association. The associations of *GSTM1* deletion with LC risk was estimated by calculating pooled risk ratio (RR)s and 95% CI between cases and controls, using StatsDirect version 3.0 software (Cheshire, UK). Pooled RR has been used in most recent consensus reports for standardized risk ratios for more conservative reports and for standardization across all factors included in the gene-environment interaction analysis [20].

We utilized JMP pro 13 program (SAS Institute, Cary, NC) for meta-prediction analysis to examine the association of air pollution associated death (APD) to *GSTM1* deletion polymorphisms and LC risk. We used partition tree to examine the association between independent and dependent variables. The “goodness of the partition” can be judged using Akaike’s information criterion (AIC) or AIC with correction (AICc), in which a smaller AIC or AICc suggests a better model [52, 61]. AIC is a fitness index for trading off the complexity of a model against how well the model fits the data. Increasing the number of free parameters to be estimated improves the model fitness, however, the model might be unnecessarily complex. To reach a balance between fitness and parsimony, the “best” model is the one with the lowest AIC value. In this sense, AIC is better than R^2^ and adjusted R^2^ used in meta-regression [20], which always go up as additional variables enter in the model, favoring complexity. However, AIC does not necessarily change by adding variables. Rather, it varies based upon the composition of the predictors and thus it is a better indicator of the model quality.

Additionally, we used nonlinear fit, heat maps, and Tukey’s posthoc test to further validate meta-prediction findings. In particular, we used Tukey’s tests to compare AICc results with the partition trees [62]. All *p* values were two-tailed with a significant level at *P <* 0.05. GIS maps was prepared to better visualize the heterogeneity of *GSTM1* deletion polymorphism with LC risks on the world map. We applied meta-predictive analytical techniques using recursive partition tree, nonlinear fit and heat maps for data visualization to reveal nonlinear patterns in this study, in addition to the conventional pooled-analysis technique, to visualize the heterogeneity. While meta-regression is used commonly for advanced meta-analysis for meta-prediction [20], it is important to point out that regression analysis, as a linear model, is unable to detect nonlinear patterns. Further, it is well known that regression based on R^2^ tends to yield a complex and overfitted model because R^2^ always goes up with additional predictors. On the other hand, AIC or AICc does not necessarily change with the addition of variables. Rather, it varies based upon the composition of the predictors; thus, it is more likely to yield an optimal model [63–65].

## SUPPLEMENTARY MATERIALS FIGURES AND TABLES




