# Correction: Lung cancer susceptibility from *GSTM1* deletion and air pollution with smoking status: a meta-prediction of worldwide populations

**DOI:** 10.18632/oncotarget.26388

**Published:** 2018-11-16

**Authors:** Pojui Yu, Joyce D. Kusuma, Maria Aurora R. Suarez, Shyang-Yun Pamela Koong Shiao

**Affiliations:** ^1^ Department of Nursing, Fu Jen Catholic University Hospital, New Taipei City, Taiwan (R.O.C.); ^2^ School of Nursing, College of Medicine, National Taiwan University, Taipei, Taiwan (R.O.C.); ^3^ Heritage Victor Valley Medical Group, Big Bear Lake, CA, USA; ^4^ Department of Critical Care and Telemetry, Citrus Valley Health Partners, West Covina, CA, USA; ^5^ College of Nursing and Medical College of Georgia, Augusta University, Augusta, GA, USA

**This article has been corrected:** The correct affiliations information is given below:

^3^ Heritage Victor Valley Medical Group, Big Bear Lake, CA, USA

^4^ Department of Critical Care and Telemetry, Citrus Valley Health Partners, West Covina, CA, USA

Original article: Oncotarget. 2018; 9:31120-31132. https://doi.org/10.18632/oncotarget.25693

