# Supplementary material for: Lung cancer susceptibility from GSTM1 deletion and air pollution with smoking status: a meta-prediction of worldwide populations
Source: Oncotarget. 2018 Jul 24;9(57):31120–32. doi: 10.18632/oncotarget.25693 (PMC6089566; doi:10.18632/oncotarget.25693)
Supplement: Supplementary file 1 [file oncotarget-09-31120-s001.pdf]

Lung cancer susceptibility from *GSTM1* deletion and air pollution with smoking status: a meta-prediction of worldwide populations

SUPPLEMENTARY MATERIALS

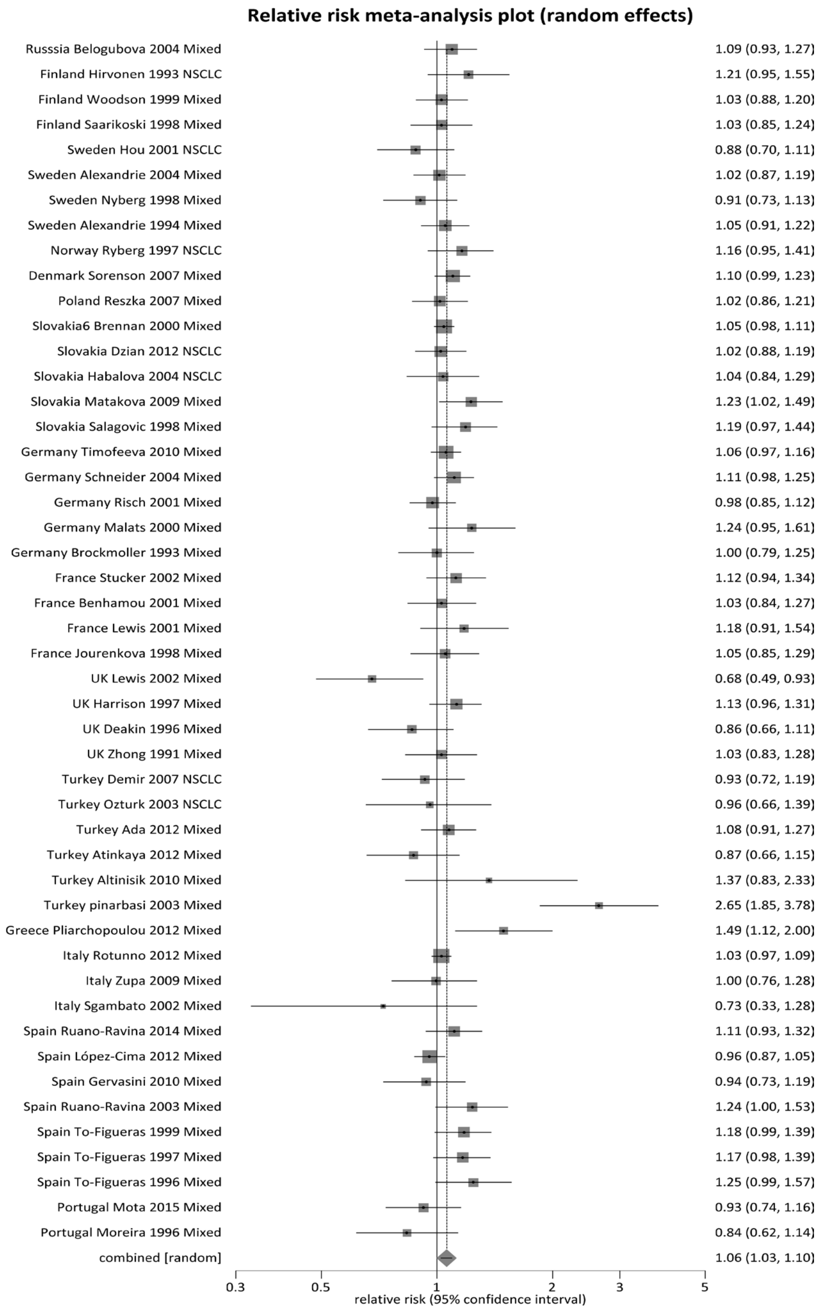

Supplementary Figure 1A: Forest plot for meta-analysis of *GSTM1* deletion type, European countries. Note. NSCLC = non-small cell lung cancer; Mixed = NSCLC and small cell lung cancer.

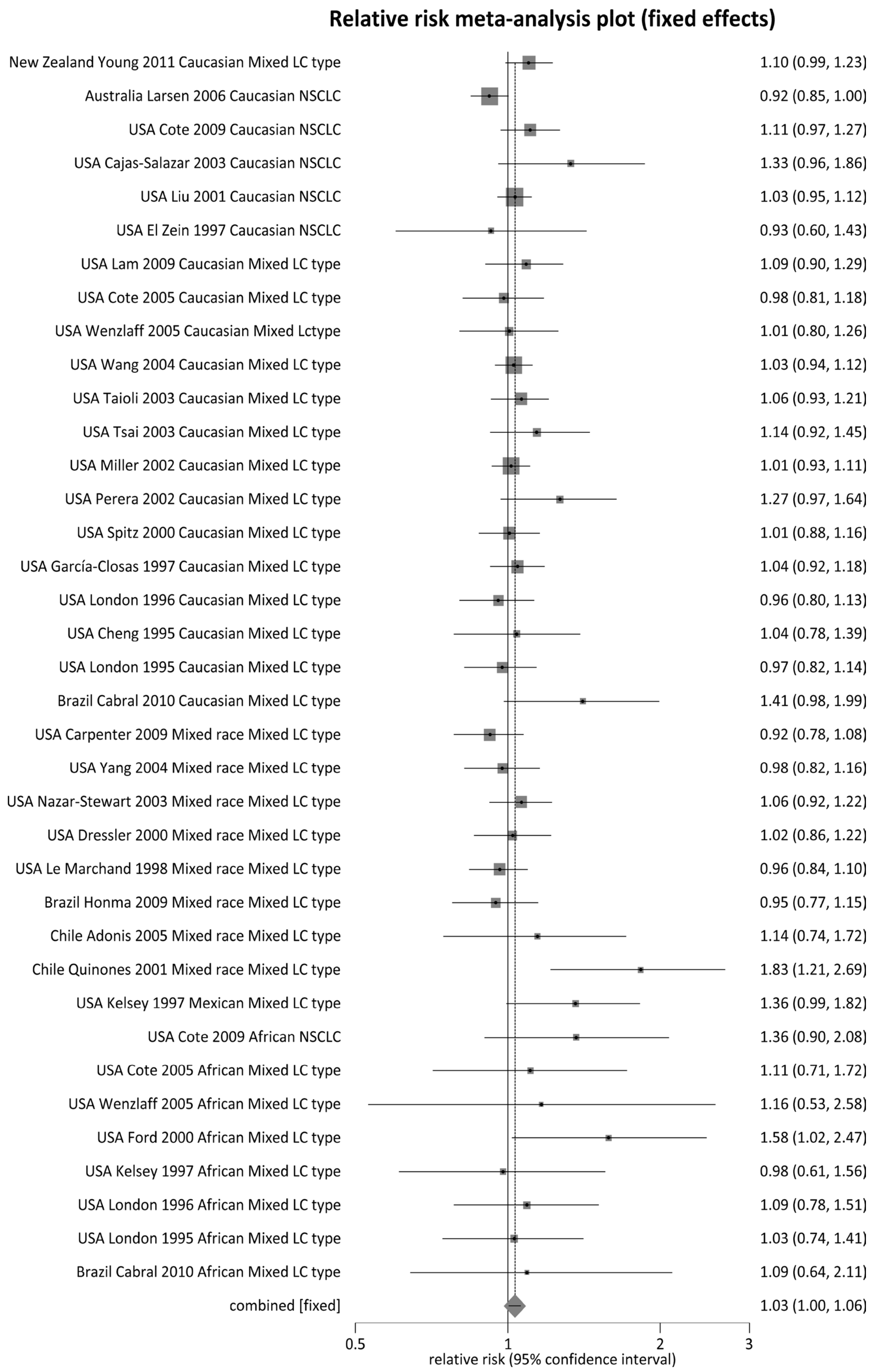

Supplementary Figure 1B: Forest plot for meta-analysis of *GSTM1* deletion type, Oceana and America. Note. Mixed race = Caucasian, African and other race; NSCLC = non small cell lung cancer; Mixed = NSCLC and small cell lung cancer.

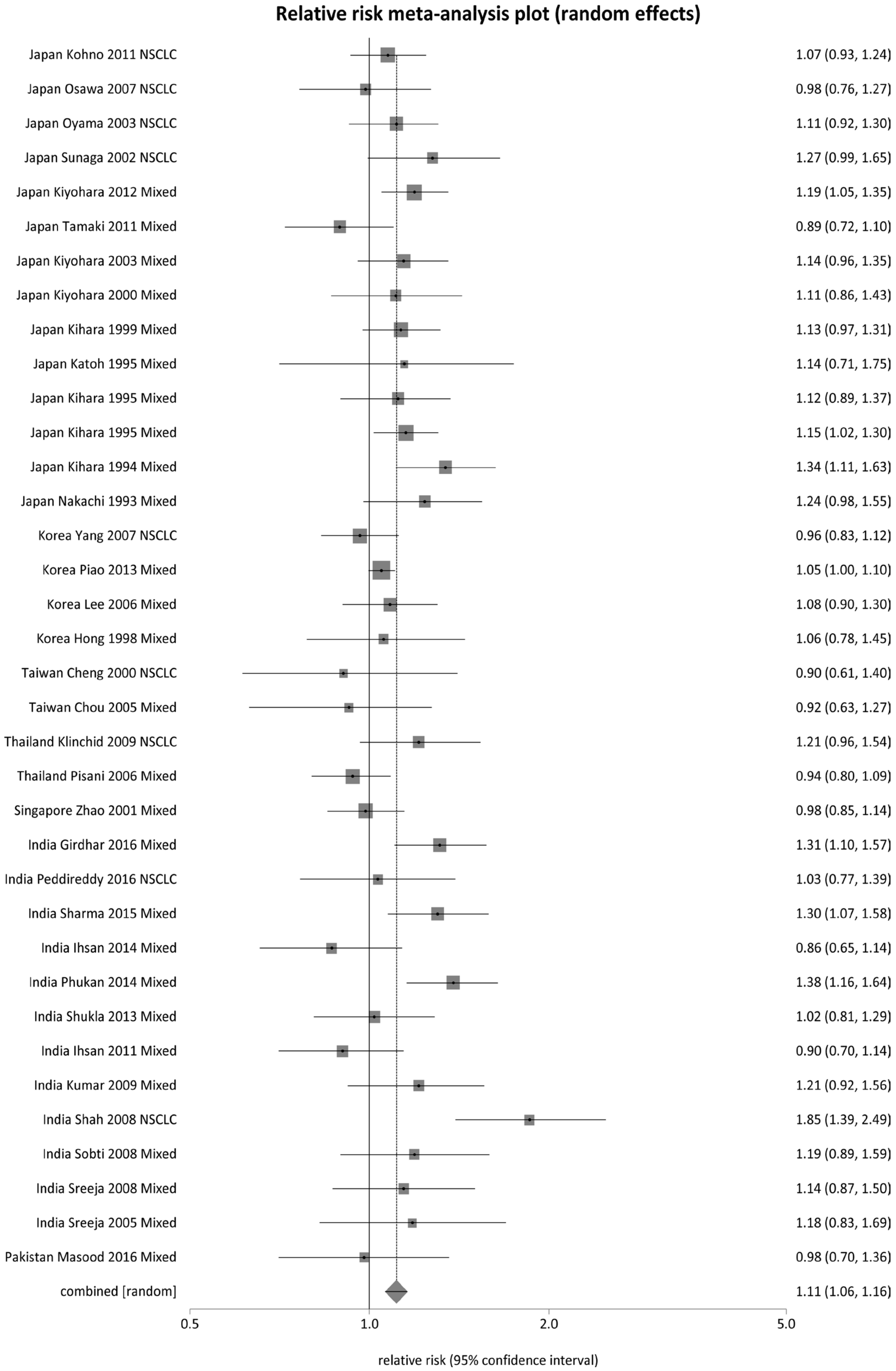

Supplementary Figure 1C: Forest plot for meta-analysis of *GSTM1* deletion type, Asian countries except China. Note. NSCLC = non-small cell lung cancer; Mixed = NSCLC and small cell lung cancer.

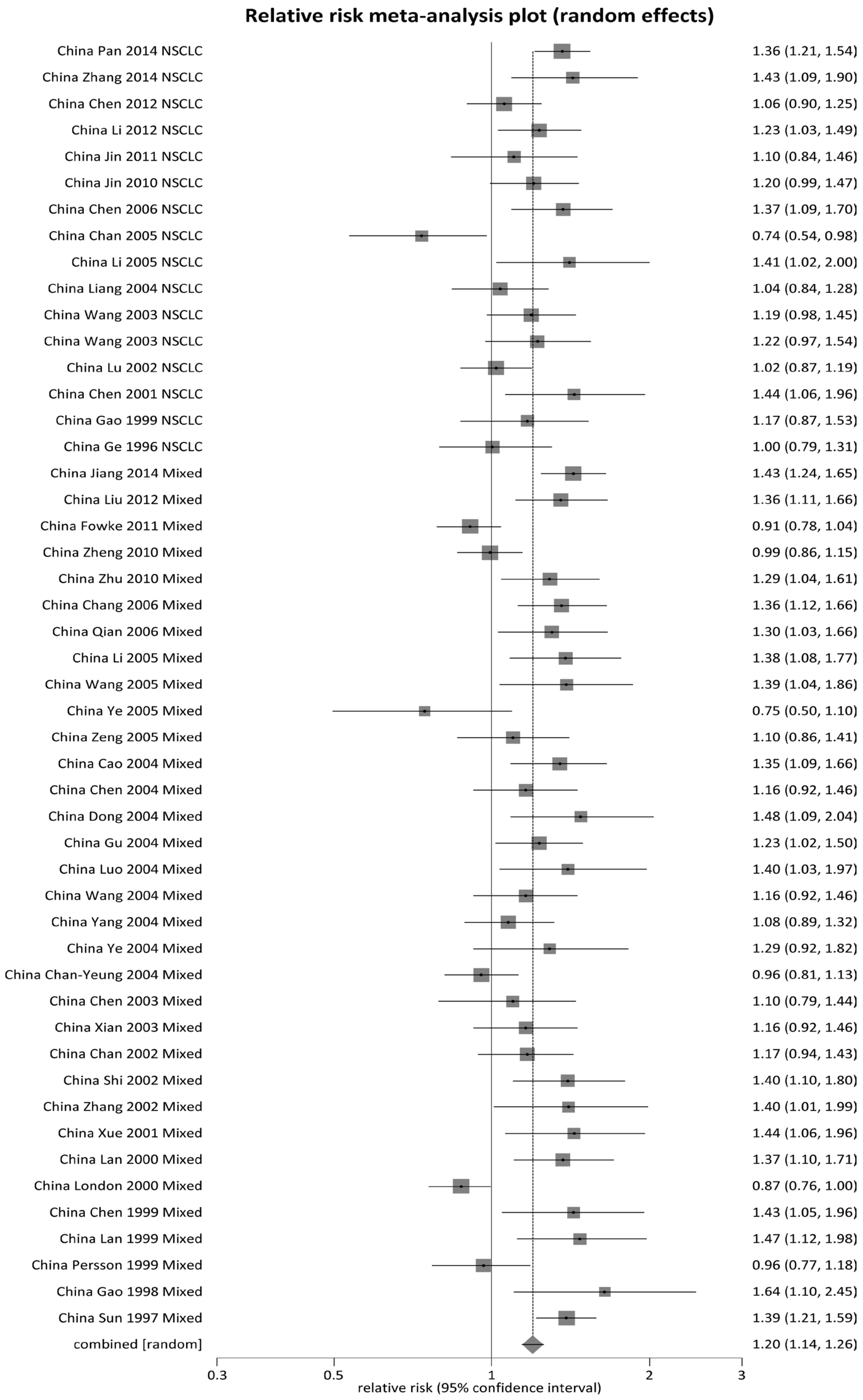

Supplementary Figure 1D: Forest plot for meta-analysis of *GSTM1* deletion type, China. Note. NSCLC = non-small cell lung cancer; Mixed = NSCLC and small cell lung cancer.

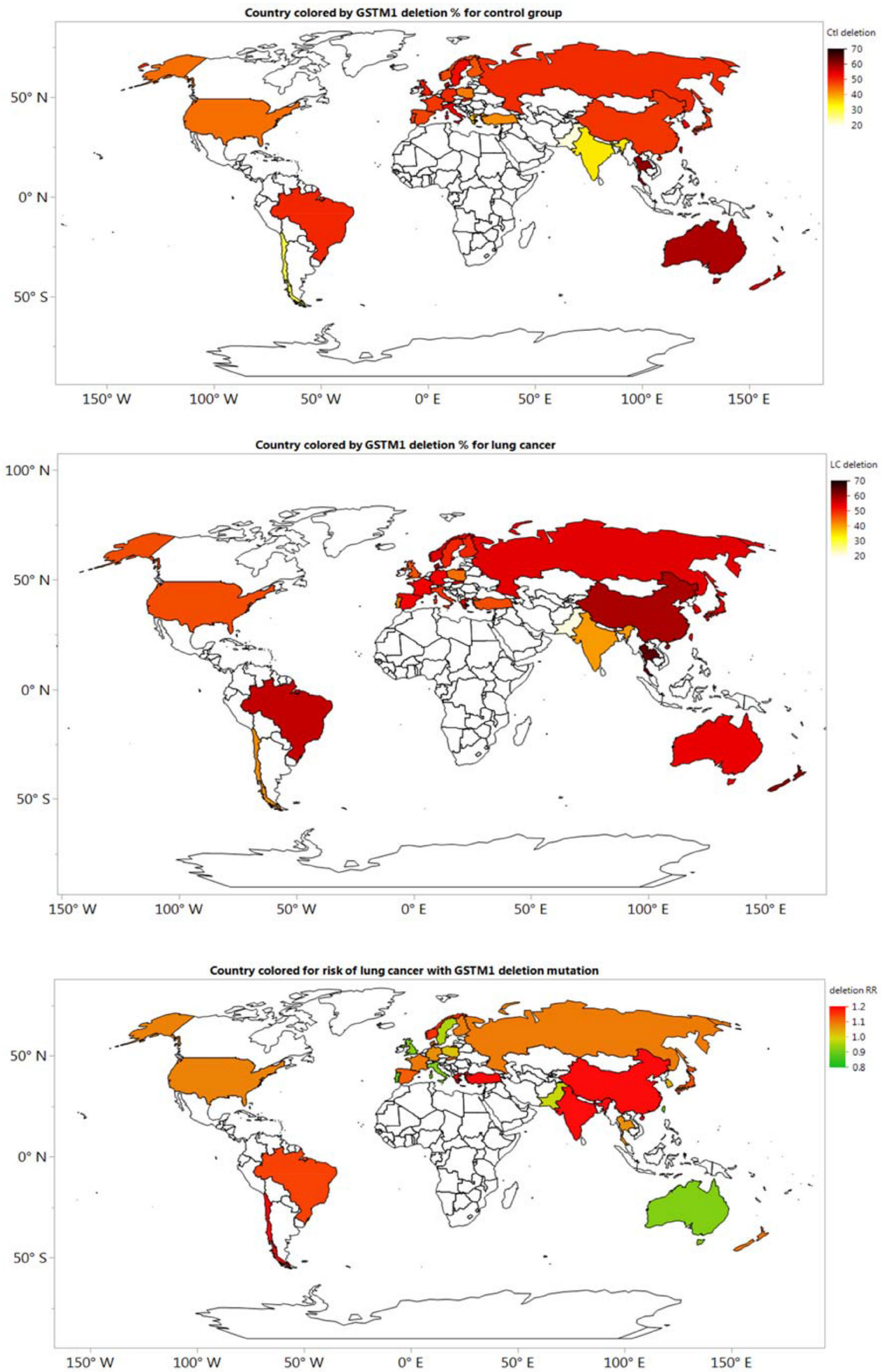

Supplementary Figure 2: Geographic information maps for total % *GSTM1* deletion per control and lung cancer (LC) groups, and LC risks.

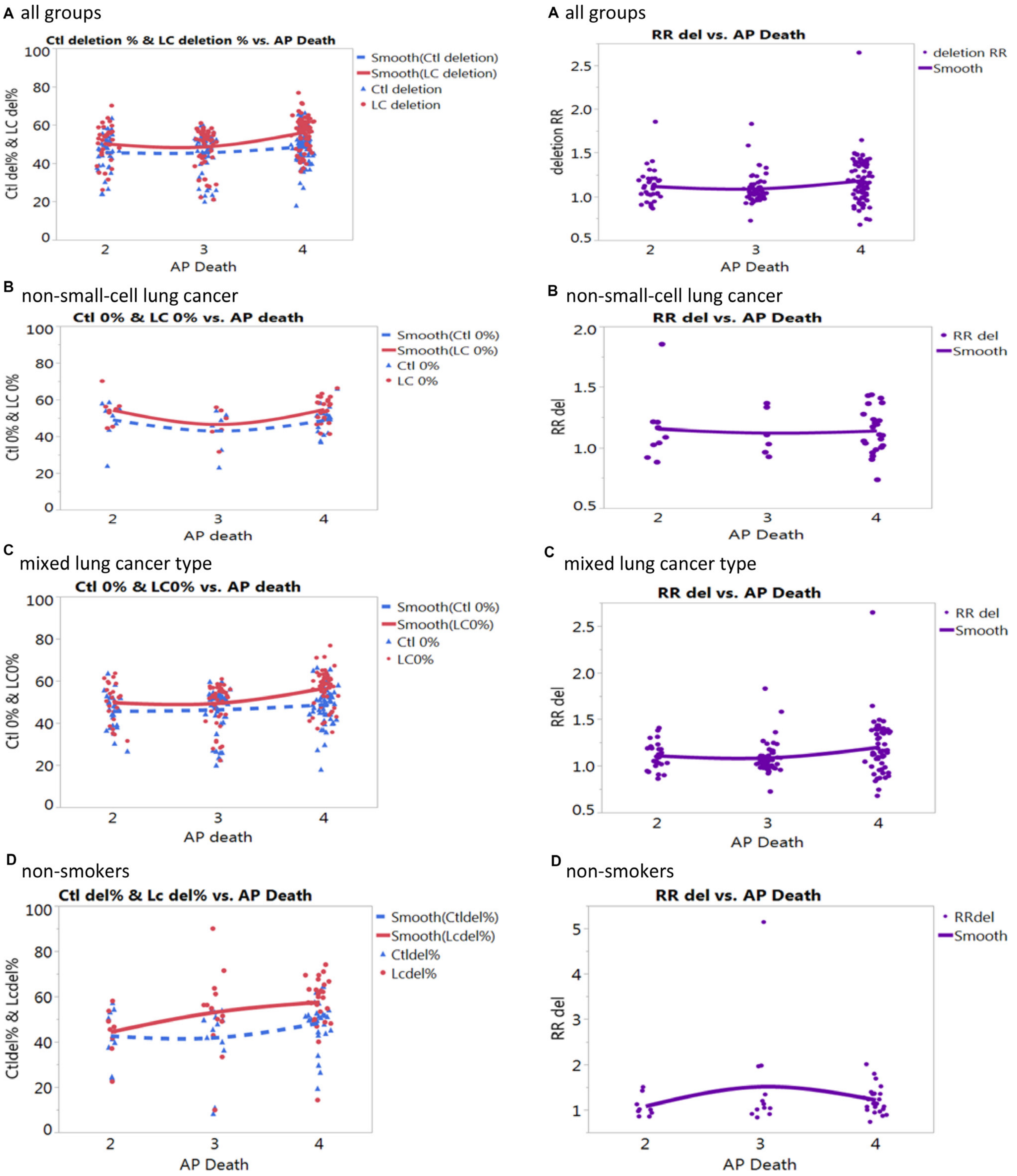

Supplementary Figure 3: Nonlinear fit on percentages of *GSTM1* deletion per control (blue color) and lung cancer (LC, red color) groups (left) and LC risk (right) with death from air pollution in: (**A**) all groups, (**B**) non-small-cell LC, (**C**) mixed LC, and (**D**) non-smokers (AP death: Death from air pollution, Levels per million: 2: ≤ 100, 3:101–250, 4: ≥ 251).

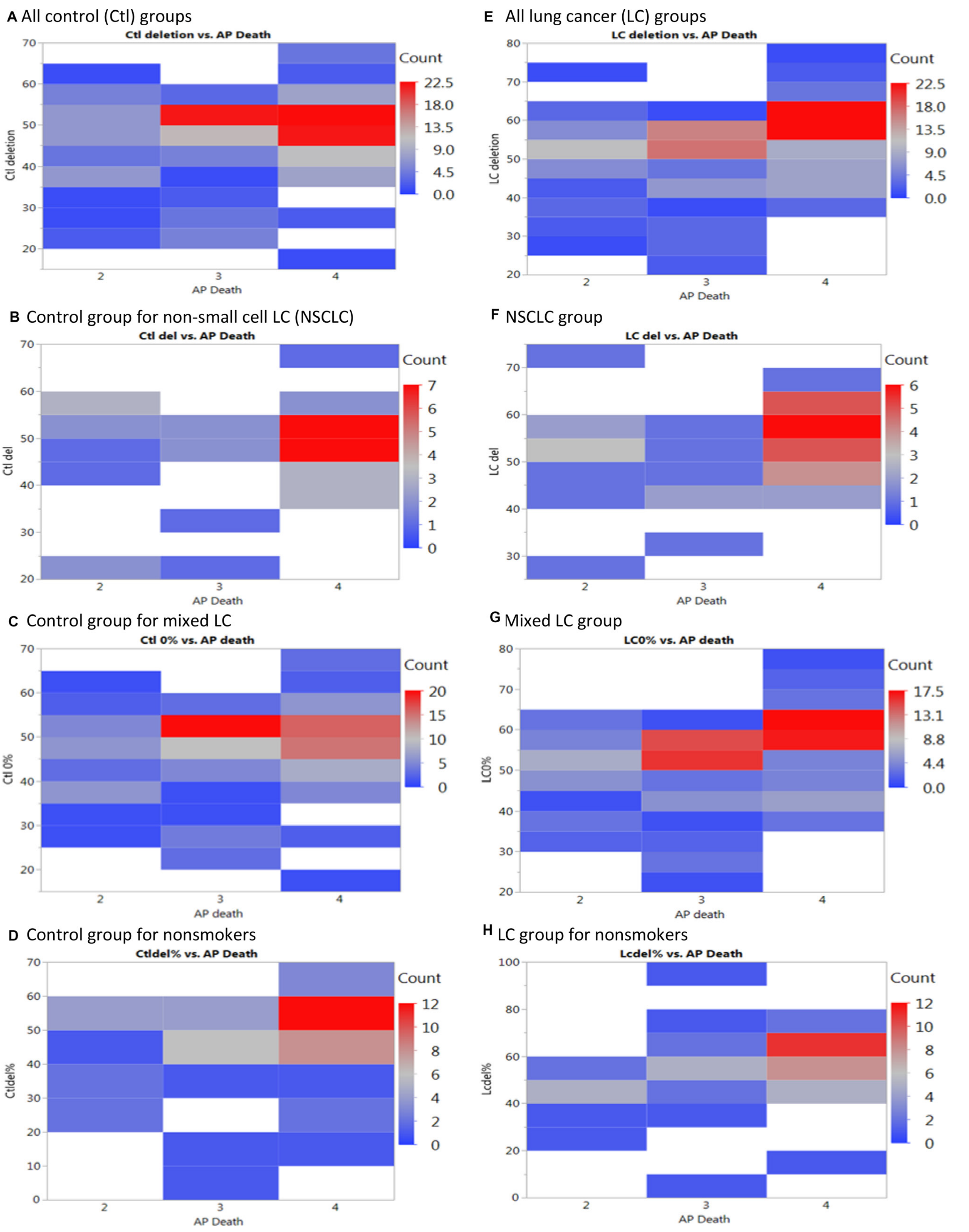

Supplementary Figure 4: Heat maps of *GSTM1* deletion for (**A**) all control groups, (**B**) control group for non-small cell lung cancer (NSCLC), (**C**) control group for mixed LC group, (**D**) control group for nonsmokers, (**E**) for all LC groups, (**F**), NSCLC group, (**G**) mixed LC group, and (**H**) LC group for nonsmokers with death from air pollution (Levels per million: 2: < 100, 3:101–250, 4: > 251).

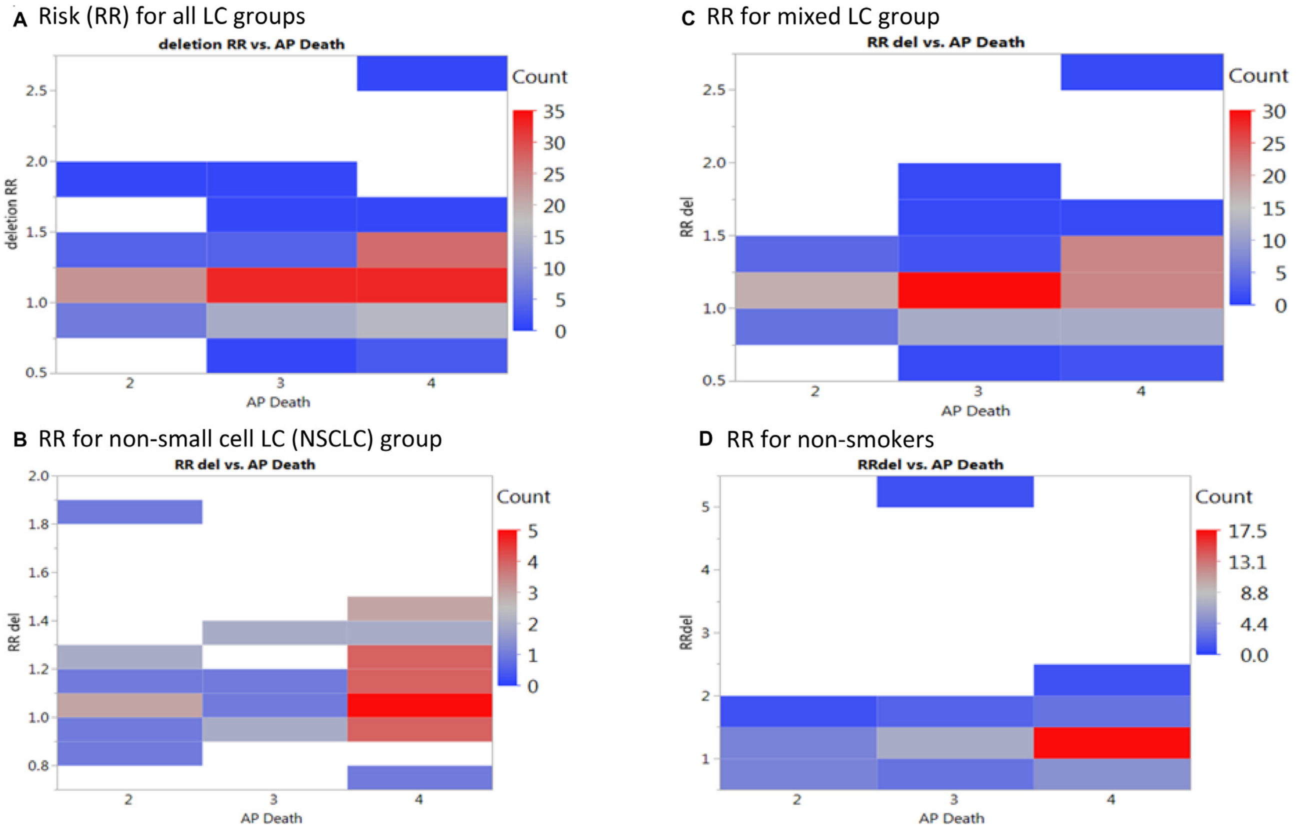

Supplementary Figure 5: Heat maps of *GSTM1* deletion for lung cancer risk in (**A**) all lung cancer (LC) groups, (**B**) non-small cell LC (NSCLC), (**C**) mixed LC group, and (**D**) nonsmokers LC group, with death from air pollution (Levels per million: 2: < 100, 3:101–250, 4: > 251).

Supplementary Table 1: Characteristics of studies included in the meta-analysis by continents in the world (170 studies). See Supplementary_Table_1

Supplementary Table 2: Pooled analysis: *GSTM1* deletion and risk of lung cancer with lung cancer types

Genotype (number of studies)	LC Case $N = 21,248$ n (%)	Control $N = 23,513$ n (%)	Test of heterogeneity			Statistical model	Test of association	
			Q	p	I^2 (%)		Risk ratio (95% CI)	p
NSCLC [38]	4,167 (19.6)	4,069 (17.3)	85.03	<0.0001	56.5%	Random	1.11 (1.06–1.17)	<0.0001
European [7]	497 (7.2)	630 (7.5)	5.74	0.4532	0.0%	Fixed	1.03 (0.95–1.12)	0.4165
Oceanian [1]	591 (68.3)	367 (58.1)						
American [4]	798 (27.7)	820 (20.4)	3.03	0.3866	1.1%	Fixed	1.06 (0.99–1.13)	0.0831
Mixed [0]								
African American [1]	36 (15.5)	28 (10.9)						
Mexican [0]								
North Asian [5]	578 (13.9)	774 (25.7)	4.19	0.3808	4.5%	Fixed	1.06 (0.98–1.15)	0.1545
Chinese [16]	1,483 (37.1)	1,338 (30.1)	29.63	0.0133	49.4%	Random	1.17 (1.08–1.26)	<0.0001
South East Asian [4]	248 (17.6)	175 (11.7)	11.16	0.0109	73.1%	Random	1.22 (0.91–1.63)	0.1746
Mixed [132]	17,050 (80.4)	19,440 (82.7)	272.89	<0.0001	52.0%	Random	1.10 (1.07–1.13)	<0.0001
European [41]	6,418 (92.8)	7,742 (92.5)	74.56	0.0007	46.4%	Random	1.06 (1.03–1.10)	0.0007
Oceanian [1]	274 (31.7)	265 (41.9)						
American [14]	2,078 (72.3)	3,206 (79.6)	8.57	0.8049	0.0%	Fixed	1.03 (0.99–1.07)	0.1123
Mixed [8]	768 (100.0)	1,216 (100.0)	11.68	0.1116	40.1%	Fixed	1.00 (0.94–1.07)	0.9213
African American [7]	197 (84.5)	230 (89.1)	2.93	0.8177	0.0%	Fixed	1.12 (0.95–1.32)	0.1637
Mexican [1]	33 (100.0)	59 (100.0)	—	—	—	—	—	
North Asian [13]	3,581 (86.1)	2,232 (74.3)	14.56	0.2662	17.6%	Fixed	1.09 (1.05–1.13)	<0.0001
Chinese [33]	2,509 (62.9)	3,114(69.9)	92.51	<0.0001	65.4%	Random	1.21 (1.14–1.30)	<0.0001
South East Asian [14]	1,159 (82.4)	1,317 (87.3)	28.06	0.0089	53.7%	Random	1.09 (1.00–1.19)	0.0585

Note. Data Included from 170 studies. *GSTM1 = Glutathione S-transferase Mu 1*; NSCLC = Non-small cell lung cancer; Q = Cochran's Q; RR = relative risk; CI = confidence interval; - = no data.

Supplementary Table 3: Pooled analysis: *GSTM1* deletion and risk of lung cancer with smoking status

Genotype (number of studies)	LC Case *N* = 21,248 *n* (%)	Control *N* = 23,513 *n* (%)	Test of heterogeneity			Statistical model	Test of association	
			Q	*p*	I^2 (%)		Risk ratio (95% CI)	*p*
**Smoker [63]**	7,776 (37.4)	6,756 (29.8)	121.19	<0.0001	49.7%	Random	1.09 (1.05–1.13)	<0.0001
European [18]	3,766 (57.6)	3,400 (43.7)	17.08	0.4489	0.5%	Fixed	1.02 (0.98–1.05)	0.2919
Oceanian [1]	591 (68.3)	367 (58.1)						
American [8]	1,094 (38.4)	1,329 (34.1)	3.63	0.8216	0.0%	Fixed	1.03 (0.97–1.09)	0.2994
Mixed [1]	136 (17.7)	171 (14.1)						
African American [1]	33 (14.2)	22 (8.5)						
Mexican [0]								
North Asian [7]	685 (16.6)	509 (17.4)	2.47	0.8722	0.0%	Fixed	1.18 (1.09–1.28)	<0.0001
Chinese [19]	1,154 (28.6)	749 (16.8)	36.89	0.0054	51.2%	Random	1.27 (1.15–1.40)	<0.0001
South East Asian [8]	317 (23.3)	209 (14.6)	6.47	0.3724	7.3%	Fixed	0.96 (0.82–1.12)	0.5974
**Non-smoker [49]**	1,513 (7.3)	3,494 (15.4)	69.97	0.0209	31.4%	Random	1.15 (1.09–1.22)	<0.0001
European [12]	323 (4.9)	1,277 (16.4)	20.80	0.0354	47.1%	Random	1.08 (0.94–1.23)	0.2632
Oceanian [0]								
American [5]	61 (2.1)	509 (13.0)	3.18	0.5278	0.0%	Fixed	1.12 (0.93–1.35)	0.2169
Mixed [1]	7 (0.9)	74 (6.1)						
African American [1]	3 (1.3)	2 (0.8)						
Mexican [0]								
North Asian [5]	211 (5.1)	335 (11.4)	8.11	0.0877	50.7%	Fixed	1.11 (0.98–1.26)	0.1012
Chinese [18]	712 (17.7)	923 (20.6)	21.01	0.226	19.1%	Fixed	1.22 (1.14–1.31)	<0.0001
South East Asian [7]	196 (14.4)	374 (26.2)	4.10	0.5354	0.0%	Fixed	1.16 (0.96–1.39)	0.1191
**NA [104]**	11,508 (55.3)	12,430 (54.8)	243.89	<0.0001	58.2%	Random	1.11 (1.07–1.14)	<0.0001
European [28]	2,444 (37.4)	3,108 (39.9)	61.32	0.0002	56.0%	Random	1.07 (1.01–1.14)	0.0217
Oceanian [1]	274 (31.7)	265 (41.9)						
American [10]	1,695 (59.5)	2,063 (52.9)	6.78	0.6605	0.0%	Fixed	1.03 (0.98–1.08)	0.221
Mixed [7]	625 (81.4)	970 (79.8)	10.88	0.092	44.9%	Fixed	0.99 (0.92–1.06)	0.769
African American [7]	196 (84.5)	234 (90.7)	1.43	0.9639	0.0%	Fixed	1.10 (0.94–1.30)	0.2431
Mexican [1]	33 (100.0)	59 (100.0)	—	—	—	—	—	
North Asian [10]	3,232 (78.3)	2,088 (71.2)	7.99	0.5351	0.0%	Fixed	1.06 (1.01–1.10)	0.0073
Chinese [30]	2,162 (53.7)	2,798 (62.6)	96.88	<0.0001	70.1%	Random	1.19 (1.11–1.29)	<0.0001
South East Asian [10]	847 (62.3)	845 (59.2)	27.54	0.0011	67.3%	Random	1.20 (1.06–1.37)	0.0051

Note. Data Included from 170 studies, 46 studies had data for both smoker and non-smoker;. *GSTM1* = Glutathione S-transferase Mu 1; Q = Cochran's Q; RR = relative risk; CI = confidence interval; NA = not available; - = no data.

Supplementary Table 4: Pooled analysis: *GSTM1* deletion and risk of lung cancer with gender status

Genotype (number of studies)	LC Case $N = 21{,}248$ n (%)	Control $N = 23{,}513$ n (%)	Test of heterogeneity			Statistical model	Test of association	
			Q	p	I^2 (%)		Risk ratio (95% CI)	p
**Male [25]**	5,072 (24.1)	3,883 (16.7)	58.69	0.0003	55.7%	Random	1.08 (1.02–1.13)	0.0045
European [11]	2,160 (30.1)	2,303 (27.3)	10.69	0.3825	6.4%	Fixed	1.04 (0.99–1.09)	0.0959
Oceanian [1]	420 (48.6)	247 (39.1)						
American [1]	35 (1.2)	95 (2.4)						
Mixed [1]	53 (6.9)	35 (2.9)						
African American [0]	—	—						
Mexican [0]	—	—						
North Asian [6]	2,439 (58.6)	1,034 (34.4)	3.64	0.6023	0.0%	Fixed	1.08 (1.02–1.14)	0.0039
Chinese [3]	268 (6.7)	340 (7.6)	15.31	0.0005	86.9%	Random	1.08 (0.77–1.50)	0.6621
South East Asian [2]	117 (8.4)	76 (5.2)	3.66	0.0558	72.7%	Fixed	1.57 (1.23–2.01)	0.0004
**Female [26]**	2,221 (10.5)	3,165 (13.6)	68.93	<0.00011	60.8%	Random	1.13 (1.06–1.22)	0.0003
European [10]	555 (7.7)	1,058 (12.5)	7.81	0.5533	0.0%	Fixed	1.01 (0.94–1.09)	0.7219
Oceanian [1]	171 (19.8)	120 (19.0)						
American [2]	250 (8.7)	324 (8.0)	0.33	0.5685	0.0%	Fixed	1.09 (0.97–1.22)	0.1669
Mixed [1]	50 (6.5)	63 (5.2)						
African American [1]	36 (15.5)	28 (10.9)						
Mexican [0]	—	—						
North Asian [4]	621 (14.9)	710 (23.6)	11.87	0.0079	74.7%	Random	1.21 (1.01–1.46)	0.0403
Chinese [5]	418 (10.5)	689 (15.5)	25.19	<0.0001	84.1%	Random	1.24 (0.96–1.60)	0.0951
South East Asian [2]	120 (8.7)	173 (11.8)	0.53	0.4662	0.0%	Fixed	1.36 (1.14–1.62)	0.0006
**NA [139]**	13, 741 (65.4)	16,210 (69.8)	249.95	<0.0001	46.0%	Random	1.11 (1.08–1.13)	<0.0001
European [37]	4,460 (62.2)	5,087 (60.2)	67.51	0.0011	46.7%	Random	1.08 (1.03–1.13)	0.0004
Oceanian [1]	274 (31.7)	265 (41.9)						
American [16]	2,591 (90.1)	3,607 (89.6)	10.72	0.7724	0.0%	Fixed	1.03 (1.00–1.07)	0.0735
Mixed [7]	665 (86.6)	1,118 (91.9)	11.64	0.0706	48.4%	Fixed	1.00 (0.93–1.07)	0.9724
African American [7]	197 (84.5)	230 (89.1)	2.93	0.8177	0.0%	Fixed	1.12 (0.95–1.32)	0.1637
Mexican [1]	33 (100.0)	59 (100.0)	—	—	—	—	—	
North Asian [11]	1,099 (26.4)	1,262 (42.0)	11.32	0.3328	11.7%	Fixed	1.08 (1.02–1.15)	0.0084
Chinese [44]	3,306 (82.8)	3,423 (70.1)	88.21	<0.0001	51.3%	Random	1.20 (1.14–1.26)	<0.0001
South East Asian [15]	1,149 (82.9)	1,218 (83.0)	21.95	0.0797	36.2%	Fixed	1.08 (1.02–1.15)	0.0133

Note. Data Included from 170 studies, 20 studies had both male and female data; *GSTM1* = Glutathione S-transferase Mu 1; Q = Cochran's Q; RR = relative risk; CI = confidence interval; NA = not available; - = no data.
