# Supplementary material for: Lung cancer susceptibility from GSTM1 deletion and air pollution with smoking status: a meta-prediction of worldwide populations
Source: Oncotarget. 2018 Jul 24;9(57):31120–32. doi: 10.18632/oncotarget.25693 (PMC6089566; doi:10.18632/oncotarget.25693)
Supplement: Supplementary file 2 [file oncotarget-09-31120-s002.docx]

**Supplementary Table 1: Characteristics of studies included in the meta-analysis by Continents in the world (170 studies)**

| **First Author, Year (reference number)** | **Ethnicity**  **-Country**  **Gender** | | **Smoking** | | | **Case, CS, n (%)** | | | | | | | | **Control, S, n (%)** | | | | | | | | | | | | **Quality score** | |  |
| --- | --- | --- | --- | --- | --- | --- | --- | --- | --- | --- | --- | --- | --- | --- | --- | --- | --- | --- | --- | --- | --- | --- | --- | --- | --- | --- | --- | --- |
|  |  |  |  |  |  | **CS** | | **Present** | | | **Deletion** | | | **S** | | | **Present** | | | **Deletion** | | | **HWE** | | |  |  |  |
| **Oceanian** |  | | |  | |  | | |  | | |  | | |  | | |  | |  | |  | | |  | | |  |
| Young, 2011  (48) | Caucasian  -New Zealand | | |  | | 1, 2 | | | 173  (38.7) | | | 274  (61.3) | | | 1 | | | 211  (44.3) | | 265  (55.7) | | no | | | 23  (7, 9, 7) | | |  |
| Larsen, 2006  (49) | Caucasian  -Australia  Male  Female | | | Yes | | 1 | | | 503  (46.0)  366 (46.6)  137 (44.5)  502  (45.9) | | | 591  (54.0)  420 (53.4)  171 (55.5)  591 (54.1) | | | 1, 2 | | | 258  (41.3)  178 (41.9)  80 (40.0)  258 (41.3) | | 367  (58.7)  247 (58.1)  120 (60.0)  367 (58.7) | | yes | | | 21  (9, 6, 6) | | |  |
|  |  | | |  | |  | | |  | | |  | | |  | | |  | |  | |  | | |  | | |  |
| **European** |  | | |  | |  | | |  | | |  | | |  | | |  | |  | |  | | |  | | |  |
| Belogubova, 2004 (50) | Caucasian  -Russia  Male  Female | | | Yes  No | | 1, 2 | | | 76  (45.5)  70  (47.3)  6  (31.6)  69  (46.6)  7  (36.8) | | | 91  (54.5)  78  (52.7)  13  (68.4)  79  (53.4)  12  (63.2) | | | 1 | | | 333  (50.2)  156  (51.5)  177  (49.2)  187  (50.1)  146  (50.3) | | 330  (49.8)  147  (48.5)  183  (50.8)  186  (49.9)  144  (49.7) | | no | | | 18  (6, 6, 6) | | |  |
| Woodson, 1999 (51) | Caucasian  -Finland  Male | | | Yes | | 1, 2 | | | 159  (49.8)  159  (49.8)  151 (49.3) | | | 160  (50.2)  160  (50.2)  155 (50.7) | | | 1 | | | 171  (51.4)  171 (51.4)  179 (52.5) | | 162  (48.6)  162 (48.6)  162 (47.5) | | no | | | 23  (8, 9, 6) | | |  |
| Saarikoski, 1998 (52) | Caucasian  -Finland | | |  | | 1, 2 | | | 108  (51.9) | | | 100  (48.1) | | | 1 | | | 157  (53.4) | | 137  (46.6) | | no | | | 17  (6, 6, 5) | | |  |
| Hirvonen, 1993 (53) | Caucasian  -Finland | | |  | | 1 | | | 65  (47.1) | | | 73  (52.9) | | | 1 | | | 80  (56.3) | | 62  (43.7) | | no | | | 15  (4, 6, 5) | | |  |
| Alexandrie, 2004 (54) | Caucasian  -Sweden | | | Yes  No | | 1, 2 | | | 237  (45.2)  102  (52.0)  13  (41.9) | | | 287  (54.8)  94  (48.0)  18  (58.1) | | | 1 | | | 240  (45.3)  130  (47.8)  99 (42.7) | | 290  (54.7)  142  (52.2)  133 (57.3) | | yes | | | 19  (8, 5, 6) | | |  |
| Hou, 2001  (55) | Caucasian  -Sweden | | | Yes  No | | 1 | | | 93  (54.7)  38  (61.3)  55  (50.9) | | | 77  (45.3)  24  (38.7)  53  (49.1) | | | 1 | | | 70  (48.6)  24  (47.1)  46 (49.5) | | 74  (51.4)  27  (52.9)  47  (50.5) | | no | | | 18  (6, 6, 6) | | |  |
| Nyberg, 1998  (56) | Caucasian  -Sweden | | | Yes  No | | 1, 2 | | | 100  (54.3)  53  (55.2)  47  (53.4) | | | 84  (45.7)  43  (44.8)  41  (46.6) | | | 1 | | | 80 (49.7)  44  (53.7)  36 (45.6) | | 81  (50.3)  38  (46.3)  43  (54.4) | | no | | | 19  (6, 8, 5) | | |  |
| Alexandrie, 1994 (57) | Caucasian  -Sweden | | |  | | 1, 2 | | | 131  (44.3) | | | 165 (55.7) | | | 1 | | | 155 (47.1) | | 174 (52.9) | | yes | | | 16  (7, 4, 5) | | |  |
| Ryberg, 1997  (58) | Caucasian  -Norway | | |  | | 1 | | | 60  (45.1) | | | 73  (54.9) | | | 1 | | | 157 (52.9) | | 140 (47.1) | | no | | | 15  (7, 3, 5) | | |  |
| Sørensen, 2007  (59) | Caucasian  -Denmark  Male  Female | | |  | | 1, 2 | | | 186 (43.5)  90  (39.6)  96  (47.8) | | | 242 (56.5)  137  (60.4)  105 (62.2) | | | 1 | | | 372 (48.7)  204  (48.1)  168 (49.4) | | 392 (51.3)  220  (51.9)  172 (50.6) | | yes | | | 20  (8, 6, 6) | | |  |
| Reszka, 2007  (60) | Caucasian  -Poland | | |  | | 1, 2 | | | 207  (55.9) | | | 163  (44.1) | | | 2 | | | 192  (56.8) | | 146  (43.2) | | no | | | 20  (7, 9, 4) | | |  |
| Brennan, 2000 (61) | Caucasian  -6^a^ countries | | | Yes  No | | 1, 2 | | | 1043  (50.5)  962  (50.4)  81  (51.9) | | | 1022  (49.4)  947  (49.6)  75  (48.1) | | | 1 | | | 1097  (52.3)  709  (51.6)  388 (54.8) | | 986  (47.3)  666  (48.4)  320 (45.2) | | no | | | 11  (5, 2, 4) | | |  |
| Dzian, 2012  (62) | Caucasian  -Slovakia | | |  | | 1 | | | 100  (43.5) | | | 130  (56.5) | | | 2 | | | 130  (44.8) | | 160  (55.2) | | yes | | | 17  (8, 3, 6) | | |  |
| Matakova, 2009 (63) | Caucasian  -Slovakia | | |  | | 1, 2 | | | 66  (41.3) | | | 94  (58.8) | | | 1 | | | 115  (52.3) | | 105  (47.7) | | yes | | | 28  (10, 12, 6) | | |  |
| Habalová, 2004 (64) | Caucasian  -Slovakia  Male  Female | | | Yes  No | | 1 | | | 53  (43.8)  44  (42.7)  9  (50.0)  47  (42.7)  6  (54.5) | | | 68  (56.2)  59  (57.3)  9  (50.0)  63  (57.3)  5  (45.5) | | | 1 | | | 69  (46.0)  53 (46.5)  16 (44.4)  40 (45.5)  29 (46.8) | | 81  (54.0)  61  (53.5)  20  (55.6)  48  (54.5)  33  (53.2) | | no | | | 16  (7, 4, 5) | | |  |
| Salagovic, 1998 (65) | Caucasian  -Slovakia | | |  | | 1, 2 | | | 48  (41.0) | | | 69  (59.0) | | | 1 | | | 125  (50.4) | | 123  (49.6) | | no | | | 16  (3, 8, 5) | | |  |
| Timofeeva, 2010 (66) | Caucasian  -Germany  Male  Female | | | Yes | | 1, 2 | | | 279 (45.5)  185  (47.1)  94  (42.7)  262 (45.9) | | | 334 (54.5)  208  (52.9)  126 (57.3)  309 (54.1) | | | 1 | | | 607 (48.5)  397  (51.0)  210 (44.5)  398 (49.3) | | 644 (51.5)  382  (49.0)  262 (55.5)  410 (50.7) | | yes | | | 18  (7, 5, 6) | | |  |
| Schneider, 2004 (67) | Caucasian  -Germany | | | Yes  No | | 1, 2 | | | 212 (47.5)  191 (48.0)  7  (38.9) | | | 234 (52.5)  207 (52.0)  11  (61.1) | | | 2 | | | 328 (52.7)  253 (53.9)  74 (49.0) | | 294 (47.3)  216 (46.1)  77  (51.0) | | no | | | 20  (7, 7, 6) | | |  |
| Risch, 2001  (68) | Caucasian  -Germany  Male  Female | | |  | | 1, 2 | | | 183 (47.8)  105  (45.5)  28  (44.4) | | | 200 (52.2)  126  (54.5)  35  (55.6) | | | 2 | | | 161 (46.5)  99  (43.0)  35 (53.8) | | 185 (53.5)  131  (57.0)  30  (46.2) | | yes | | | 20  (7, 7, 6) | | |  |
| Malats, 2000  (69) | Caucasian  -Germany | | |  | | 1, 2 | | | 56  (45.9) | | | 66  (54.1) | | | 1, 2 | | | 68 (56.2) | | 53  (43.8) | | no | | | 20  (7, 7, 6) | | |  |
| Brockmöller, 1993 (70) | Caucasian  -Germany  Male  Female | | | Yes  No | | 1, 2 | | | 55  (47.0)  43  (47.8)  12  (42.9)  51  (45.9)  4  (66.7) | | | 62  (53.0)  46  (52.2)  16  (57.1)  60  (54.1)  2  (33.3) | | | 2 | | | 73 (47.1)  42  (50.6)  31  (43.1)  61  (45.2)  12 (60.0) | | 82  (52.9)  41  (49.4)  41  (56.9)  74  (54.8)  8  (40.0) | | no | | | 21  (6, 10, 5) | | |  |
| Stücker, 2002  (71) | Caucasian  -France | | | Yes | | 1, 2 | | | 118 (47.8)  143 (60.1) | | | 129 (52.2)  95  (39.9) | | | 2 | | | 136 (53.5)  117 (49.2) | | 118 (46.5)  121 (50.8) | | no | | | 17  (7, 5, 5) | | |  |
| Benhamou, 2001 (72) | Caucasian  -France | | |  | | 1, 2 | | | 69  (46.0) | | | 81  (54.0) | | | 2 | | | 82 (47.7) | | 90  (52.3) | | yes | | | 26  (8, 12, 6) | | |  |
| Lewis, 2001  (73) | Caucasian  -France | | |  | | 1, 2 | | | 57  (47.9) | | | 62  (52.1) | | | 1 | | | 67 (55.8) | | 53  (44.2) | | no | | | 8  (4, 1, 3) | | |  |
| Jourenkova, 1998 (74) | Caucasian  -France | | | Yes | | 1, 2 | | | 66  (45.2)  78  (53.4) | | | 80  (54.8)  68  (46.6) | | | 2 | | | 81 (47.9)  99 (58.6) | | 88  (52.1)  70  (41.4) | | yes | | | 22  (8, 8, 6) | | |  |
| Lewis, 2002  (75) | Caucasian  -UK  Male  Female | | |  | | 1, 2 | | | 56  (64.4)  37  (66.1)  19  (61.3) | | | 31  (35.6)  19  (33.9)  12  (38.7) | | | 2 | | | 68 (47.6)  37  (45.7)  31 (50.0) | | 75  (52.4)  44  (54.3)  31  (50.0) | | no | | | 17  (7, 4, 6) | | |  |
| Harrison, 1997 (76) | Caucasian  -UK | | |  | | 1, 2 | | | 67  (39.9) | | | 101 (60.1) | | | 1 | | | 179 (46.6) | | 205 (53.4) | | no | | | 17  (7, 5, 5) | | |  |
| Deakin, 1996  (77) | Caucasian  -UK | | |  | | 1, 2 | | | 56  (52.8) | | | 50  (47.2) | | | 2 | | | 58 (45.3) | | 70  (54.7) | | no | | | 11  (4, 5, 2) | | |  |
| Zhong, 1991  (78) | Caucasian  -UK | | |  | | 1, 2 | | | 130 (57.0) | | | 98  (43.0) | | | 1 | | | 131 (58.2) | | 94  (41.8) | | no | | | 17  (4, 9, 4) | | |  |
| Ada, 2012  (79) | Caucasian  -Turkey | | |  | | 1, 2 | | | 90  (42.3) | | | 123 (57.7) | | | 2 | | | 107 (46.3) | | 124 (53.7) | | no | | | 18  (6, 6, 6) | | |  |
| Atinkaya, 2012 (80) | Caucasian  -Turkey | | |  | | 1, 2 | | | 72  (57.6) | | | 53  (42.4) | | | 1 | | | 59 (51.3) | | 56  (48.7) | | no | | | 17  (5, 6, 6) | | |  |
| Altinisik, 2010 (81) | Caucasian  -Turkey | | | Yes  No | | 1, 2 | | | 47  (62.7)  40  (59.7)  6  (85.7) | | | 28  (37.3)  27  (40.3)  1  (14.3) | | | 1 | | | 40 (72.7)  11  (57.9)  29 (80.6) | | 15  (27.3)  8  (42.1)  7  (19.4) | | no | | | 15  (6, 4, 5) | | |  |
| Demir, 2007  (82) | Caucasian  -Turkey | | |  | | 1 | | | 52  (51.0) | | | 50  (49.0) | | | 1 | | | 73 (47.4) | | 81  (52.6) | | no | | | 21  (6, 10, 5) | | |  |
| Pinarbasi, 2003 (83) | Caucasian  -Turkey | | |  | | 1, 2 | | | 53  (52.5) | | | 48  (47.5) | | | 1 | | | 169 (82.0) | | 37  (18.0) | | no | | | 19  (7, 6, 6) | | |  |
| Ozturk, 2003  (84) | Caucasian  -Turkey | | |  | | 1 | | | 29  (52.7) | | | 26  (47.3) | | | 1 | | | 33 (50.8) | | 32  (49.2) | | no | | | 19  (7, 7, 5) | | |  |
| Pliarchopoulou, 2012 (85) | Caucasian  -Greece | | |  | | 1, 2 | | | 45  (45.0) | | | 55  (55.0) | | | 1 | | | 79 (63.2) | | 46  (36.8) | | no | | | 20  (6, 9, 5) | | |  |
| Rotunno, 2012 (86) | Caucasian  -Italy  Male  Female | | | Yes  No | | 1, 2 | | | 906  (46.8)  720  (47.1)  186 (45.9)  833  (46.7)  67  (48.6) | | | 1028 (53.2)  809  (52.9)  219 (54.1)  951  (53.3)  71  (51.4) | | | 1 | | | 951 (48.3)  736  (49.0)  215 (46.2)  633  (47.3)  315 (50.3) | | 1017 (51.7)  767  (51.0)  250 (53.8)  704  (52.7)  311 (49.7) | | yes | | | 20  (8, 6, 6) | | |  |
| Zupa, 2009  (87) | Caucasian  -Italy  Male  Female | | |  | | 1, 2 | | | 33  (44.0)  23  (36.5)  9  (75.0) | | | 42  (56.0)  40  (63.5)  3  (25.0) | | | 1 | | | 53 (43.8)  21  (37.5)  31 (47.7) | | 68  (56.2)  35  (62.5)  34  (52.3) | | no | | | 15  (6, 5, 4) | | |  |
| Sgambato, 2002 (88) | Caucasian  -Italy | | |  | | 1, 2 | | | 8  (61.5) | | | 5  (38.5) | | | 1 | | | 47 (47.0) | | 53  (53.0) | | no | | | 16  (6, 4, 6) | | |  |
| Ruano-Ravina, 2014 (89) | Caucasian  -Spain | | | Yes | | 1, 2 | | | 158 (55.8)  127  (58.5) | | | 125 (44.2)  90  (41.5) | | | 2 | | | 291 (60.2)  120  (60.3) | | 192 (39.8)  79  (39.7) | | no | | | 21  (5, 11, 5) | | |  |
| López-Cima, 2012 (90) | Caucasian  -Spain | | | Yes  No | | 1, 2 | | | 375  (48.3)  349  (48.1)  26  (51.0) | | | 401  (51.7)  376  (51.9)  25  (49.0) | | | 2 | | | 358  (46.1)  255  (46.0)  102 (46.2) | | 418  (53.9)  299  (54.0)  119 (53.8) | | yes | | | 22  (8, 8, 6) | | |  |
| Gervasini, 2010 (91) | Caucasian  -Spain | | | Yes | | 1, 2 | | | 56  (54.4)  53  (54.6) | | | 47  (45.6)  44  (45.4) | | | 2 | | | 127 (51.4)  92 (48.4) | | 120 (48.6)  98  (51.6) | | no | | | 22  (8, 8, 6) | | |  |
| Ruano-Ravina, 2003 (92) | Caucasian  -Spain | | | Yes  No | | 1, 2 | | | 53  (42.4)  52  (45.2)  1  (10.0) | | | 72  (57.6)  63  (54.8)  9  (90.0) | | | 2 | | | 100 (53.5)  58  (52.7)  42 (54.5) | | 87  (46.5)  52  (47.3)  35  (45.5) | | no | | | 12  (4, 5, 3) | | |  |
| To-Figueras, 1999 (93) | Caucasian  -Spain | | |  | | 1, 2 | | | 68  (41.5) | | | 96  (58.5) | | | 1 | | | 167 (50.3) | | 165 (49.7) | | no | | | 16  (7, 4, 5) | | |  |
| To-Figueras, 1997 (94) | Caucasian  -Spain | | |  | | 1, 2 | | | 67  (41.9) | | | 93  (58.1) | | | 1 | | | 157 (50.3) | | 155 (49.7) | | no | | | 20  (8, 7, 5) | | |  |
| To-Figueras, 1996 (95) | Caucasian  -Spain | | |  | | 1, 2 | | | 60  (43.2) | | | 79  (56.8) | | | 1 | | | 80 (54.4) | | 67  (45.6) | | yes | | | 22  (9, 8, 5) | | |  |
| Mota, 2015  (96) | Caucasian  -Portugal  Male  Female | | |  | | 1, 2 | | | 108  (59.0)  79  (57.7)  29  (63.0) | | | 75  (41.0)  58  (42.3)  17  (37.0) | | | 2 | | | 127  (55.7)  78  (54.2)  49 (58.3) | | 101 (44.3)  66  (45.8)  35  (41.7) | | yes | | | 23  (11, 6, 6) | | |  |
| Moreria, 1996 (97) | Caucasian  -Portugal | | |  | | 1, 2 | | | 55  (56.1) | | | 43  (43.9) | | | 1 | | | 40 (47.6) | | 44  (52.4) | | no | | | 10  (2, 5, 3) | | |  |
|  | | | |  | |  | | |  | | |  | | |  | | |  | |  | |  | | |  | | |  |
|  | | | |  | |  | | |  | | |  | | |  | | |  | |  | |  | | |  | | |  |
|  | | | |  | |  | | |  | | |  | | |  | | |  | |  | |  | | |  | | |  |
| **North American** | | | |  | |  | | |  | | |  | | |  | | |  | |  | |  | | |  | | |  |
| Cote, 2009  (98) | Caucasian  -US  Female  African  -US  Female | | |  | | 1 | | | 178 (45.9)  178 (45.9)  78  (68.4)  78  (68.4) | | | 210 (54.1)  210 (54.1)  36  (31.6)  36  (31.6) | | | 1 | | | 206 (51.1)  206 (51.1)  93 (76.9)  93 (76.9) | | 197 (48.9)  197 (48.9)  28  (23.1)  28  (23.1) | | yes | | | 21  (8, 7, 6) | | |  |
| Lam, 2009  (99) | Caucasian  -US  Male  Female | | | Yes  No | | 1, 2 | | | 62  (45.3)  28  (44.4)  34  (45.9)  56  (44.8)  6  (50.0) | | | 75  (54.7)  35  (55.6)  40  (54.1)  69  (55.2)  6  (50.0) | | | 1 | | | 219 (49.7)  106  (52.7)  113  (47.1)  194  (49.4)  25 (52.1) | | 222 (50.3)  95  (47.3)  127  (52.9)  199  (50.6)  23  (47.9) | | yes | | | 24  (7,11,6) | | |  |
| Cote, 2005  (100) | Caucasian  -US  African  -US | | |  | | 1, 2 | | | 123 (53.5)  64  (71.1) | | | 107 (46.5)  26  (28.9) | | | 1 | | | 151 (52.6)  88 (73.9) | | 136 (47.4)  31  (26.1) | | yes | | | 25  (9,10,6) | | |  |
| Wenzlaff, 2005 (101) | Caucasian  -US  African  -US | | |  | | 1, 2 | | | 62  (47.3)  20  (69.0) | | | 69  (52.7)  9  (31.0) | | | 1 | | | 70 (47.6)  22 (73.3) | | 77  (52.4)  8  (26.7) | | yes | | | 23  (9, 8, 6) | | |  |
| Wang, 2004  (102) | Caucasian  -US | | | Yes  No | | 1, 2 | | | 312 (43.6)  293  (43.5)  19  (45.2) | | | 404 (56.4)  381  (56.5)  23  (54.8) | | | 1 | | | 423 (45.0)  271  (44.6)  152 (45.8) | | 516 (55.0)  336  (55.4)  180 (54.2) | | no | | | 16  (5,5,6) | | |  |
| Cajas-Salazar, 2003 (103) | Caucasian  -US | | | Yes | | 1 | | | 62  (56.4)  46  (58.2) | | | 48  (43.6)  33  (41.8) | | | 1 | | | 80 (67.2)  40 (58.0) | | 39  (32.8)  29  (42.0) | | no | | | 19  (7,6,6) | | |  |
| Taioli, 2003  (104) | Caucasian  -US | | | Yes  No | | 1, 2 | | | 111 (47.0)  92  (46.9)  14  (43.8) | | | 125 (53.0)  104  (53.1)  18  (56.2) | | | 1 | | | 643 (50.2)  301  (50.3)  288 (50.3) | | 639 (49.8)  297  (49.7)  285 (49.7) | | no | | | 15  (6,4,5) | | |  |
| Tsai, 2003  (105) | Caucasian  -US | | |  | | 1, 2 | | | 98  (41.7) | | | 137 (58.3) | | | 1 | | | 46 (48.9) | | 48  (51.1) | | yes | | | 20  (7,7,6) | | |  |
| Miller, 2002  (106) | Caucasian  -US | | |  | | 1, 2 | | | 344 (44.9) | | | 423 (55.1) | | | 1 | | | 423 (45.6) | | 504 (54.4) | | yes | | | 26  (11, 9, 6) | | |  |
| Perera, 2002 (107) | Caucasian  -US | | | Yes  No | | 1, 2 | | | 39  (45.3)  31  (43.7)  7  (43.8) | | | 47  (54.7)  40  (56.3)  9  (56.2) | | | 2 | | | 91 (56.9)  75  (54.7)  18  (58.1) | | 69  (43.1)  62  (45.3)  13  (41.9) | | no | | | 18  (5, 7, 6) | | |  |
| Liu, 2001  (108) | Caucasian  -US | | |  | | 1 | | | 409 (44.2) | | | 517 (55.8) | | | 1 | | | 475 (45.8) | | 561 (54.2) | | yes | | | 18  (8, 5, 5) | | |  |
| Spitz, 2000  (109) | Caucasian  -US | | | Yes | | 1, 2 | | | 227 (51.5)  227 (51.5) | | | 214 (48.5)  214 (48.5) | | | 1 | | | 219 (51.8)  219 (51.8) | | 204 (48.2)  204 (48.2) | | no | | | 13  (6, 1, 6) | | |  |
| Gracia-Closas, 1997 (110) | Caucasian  -US | | | Yes | | 1, 2 | | | 190 (45.7)  179 (45.3) | | | 226 (54.3)  216 (54.7) | | | 1 | | | 214 (48.0)  139 (45.3) | | 232 (52.0)  168 (54.7) | | no | | | 18  (8, 5, 5) | | |  |
| el Zein, 1997 (111) | Caucasian  -US | | |  | | 1 | | | 31  (57.4) | | | 23  (42.6) | | | 1 | | | 27 (54.0) | | 23  (46.0) | | no | | | 17  (5, 7, 5) | | |  |
| London, 1996 (112) | Caucasian  -US  African  -US | | |  | | 1, 2 | | | 88  (49.7)  109 (71.7) | | | 89  (50.3)  43  (28.3) | | | 1 | | | 217 (47.5)  177 (74.1) | | 240 (52.5)  62  (25.9) | | no | | | 19  (7, 7, 5) | | |  |
| Cheng, 1995  (113) | Caucasian  -US | | | Yes  No | | 1, 2 | | | 32  (43.2)  30  (44.8)  2  (28.6) | | | 42  (56.8)  37  (55.2)  5  (71.4) | | | 1 | | | 35 (45.5)  21 (38.2)  14 (63.6) | | 42  (54.5)  34  (61.8)  8  (36.4) | | no | | | 19  (3,11,5) | | |  |
| London, 1995 (114) | Caucasian  -US  African  -US | | |  | | 1, 2 | | | 90  (48.9)  114 (72.2) | | | 94  (51.1)  44  (27.8) | | | 1 | | | 221 (47.5)  183 (72.9) | | 244 (52.5)  68  (27.1) | | no | | | 19  (8, 7, 4) | | |  |
| Carpenter, 2009 (115) | Mixed  -US | | |  | | 1, 2 | | | 184 (59.2) | | | 127 (40.8) | | | 1 | | | 346 (55.6) | | 276 (44.4) | | no | | | 16  (6, 4, 6) | | |  |
| Yang, 2004  (116) | Mixed  -US | | |  | | 1, 2 | | | 113 (48.1) | | | 122 (51.9) | | | 1 | | | 109 (46.8) | | 124 (53.2) | | no | | | 22  (9, 7, 6) | | |  |
| Nazar-Stewart, 2003 (117) | Mixed  -US | | | Yes  No | | 1, 2 | | | 131 (47.8)  127  (48.3)  4  (36.4) | | | 143 (52.2)  136  (51.7)  7  (63.6) | | | 1 | | | 255 (50.9)  190  (52.6)  65 (46.8) | | 246 (49.1)  171  (47.4)  74  (53.2) | | yes | | | 23  (9, 9, 5) | | |  |
| Dressler, 2000 (118) | Mixed  -US  Male  Female | | |  | | 1, 2 | | | 66  (39.1)  34  (39.1)  32  (39.0) | | | 103 (60.9)  53  (60.9)  50  (61.0) | | | 1 | | | 66 (40.2)  24  (40.7)  42 (40.0) | | 98  (56.8)  35  (59.3)  63  (60.0) | | no | | | 19  (8, 5, 6) | | |  |
| Le Marchand, 1998 (119) | Mixed  -US | | |  | | 1, 2 | | | 100  (42.6) | | | 135  (57.4) | | | 1 | | | 181  (40.3) | | 268  (59.7) | | yes | | | 23  (9, 8, 6) | | |  |
| Kelsey, 1997  (120) | Mexican  -US  African  -US | | |  | | 1, 2 | | | 27  (45.0)  84  (77.8) | | | 33  (55.0)  24  (22.2) | | | 1 | | | 87 (59.6)  102 (77.3) | | 59  (40.4)  30  (22.7) | | no | | | 16  (6, 5, 5) | | |  |
| Ford, 2000  (121) | African  -US | | | Yes  No | | 1, 2 | | | 80  (68.4)  73  (68.9)  4  (57.1) | | | 37  (31.6)  33  (31.1)  3  (42.9) | | | 2 | | | 96 (80.0)  69  (75.8)  22  (91.7) | | 24  (20.0)  22  (24.2)  2  (8.3) | | no | | | 23  (9, 8, 6) | | |  |
|  | | | |  | |  | | |  | | |  | | |  | | |  | |  | |  | | |  | | |  |
|  | | | |  | |  | | |  | | |  | | |  | | |  | |  | |  | | |  | | |  |
|  | | | |  | |  | | |  | | |  | | |  | | |  | |  | |  | | |  | | |  |
| **South American** | | | |  | |  | | |  | | |  | | |  | | |  | |  | |  | | |  | | |  |
| Cabral, 2010 (122) | Caucasian  -Brazil  African  -Brazil | | |  | | 1, 2 | | | 16  (38.1)  8  (36.4) | | | 26  (61.9)  14  (63.6) | | | 1 | | | 42 (56.0)  5 (41.7) | | 33  (44.0)  7  (58.3) | | no | | | 16  (5, 5, 6) | | |  |
| Honma, 2009 (123) | Mixed  -Brazil | | |  | | 1, 2 | | | 109 (54.4) | | | 91  (45.5) | | | 2 | | | 137 (51.9) | | 127 (48.1) | | yes | | | 19  (7, 6, 6) | | |  |
| Adonis, 2005 (124) | Mixed  -Chile | | |  | | 1, 2 | | | 33  (60.0) | | | 22  (40.0) | | | 1 | | | 67 (65.0) | | 36  (35.0) | | yes | | | 12  (3, 4, 5) | | |  |
| Quinones, 2001 (125) | Mixed  -Chile | | |  | | 1, 2 | | | 33  (56.9) | | | 25  (43.1) | | | 1 | | | 133 (76.4) | | 41  (23.6) | | no | | | 17  (6, 5, 6) | | |  |
|  |  | | |  | |  | | |  | | |  | | |  | | |  | |  | |  | | |  | | |  |
| **North East Asia** | |  | | |  | |  | | |  | | |  | | |  | | |  | |  | | |  | | |  | |
| Kiyohara, 2012 (126) | Asian  -Japan | | |  | | 1, 2 | | | 194 (42.0) | | | 268 (58.0) | | | 2 | | | 194 (51.2) | | 185 (48.8) | | no | | | 18  (6, 6, 6) | | |  |
| Kohno, 2011 (127) | Asian  -Japan | | |  | | 1b | | | 174 (46.5) | | | 200 (53.5) | | | 2 | | | 159 (50.2) | | 158 (49.8) | | no | | | 16  (7, 4, 5) | | |  |
| Tamaki, 2011 (128) | Asian  -Japan | | |  | | 1, 2 | | | 106 (55.2) | | | 86  (44.8) | | | 2 | | | 101 (49.8) | | 102 (50.2) | | yes | | | 17  (8, 3, 6) | | |  |
| Osawa, 2007 (129) | Asian  -Japan | | | Yes  No | | 1 | | | 56  (49.6)  38  (48.1)  17  (53.1) | | | 57  (50.4)  41  (51.9)  15  (46.9) | | | 2 | | | 59 (48.8)  29  (47.5)  26 (47.3) | | 62  (51.2)  32  (52.5)  29  (52.7) | | yes | | | 18  (7, 5, 6) | | |  |
| Kiyohara, 2003 (130) | Asian  -Japan  Female | | | No | | 1, 2 | | | 64  (40.5)  64  (40.5)  64  (40.5) | | | 94  (59.5)  94  (59.5)  94  (59.5) | | | 2 | | | 124 (47.9)  124 (47.9)  124 (47.9) | | 135 (52.1)  135 (52.1)  135 (52.1) | | no | | | 17  (6, 6, 5) | | |  |
| Oyama, 2003 (131) | Asian  -Japan | | |  | | 1 | | | 53  (43.1) | | | 70  (56.9) | | | 1 | | | 303 (48.7) | | 319 (51.3) | | no | | | 12  (3, 5, 4) | | |  |
| Sunaga, 2002 (132) | Asian  -Japan | | | Yes  No | | 1a | | | 105 (53.0)  69  (57.5)  35  (46.7) | | | 93  (47.0)  51  (42.5)  40  (53.3) | | | 2 | | | 96 (63.2)  57  (58.8)  36 (73.5) | | 56  (36.8)  40  (41.2)  13  (26.5) | | no | | | 22  (8, 8, 6) | | |  |
| Kiyohara, 2000 (133) | Asian  -Japan | | |  | | 1, 2 | | | 33  (38.4) | | | 53  (61.6) | | | 1 | | | 39 (44.3) | | 49  (55.7) | | no | | | 22  (7, 10, 5) | | |  |
| Kihara, 1999  (134) | Asian  -Japan  Male | | | Yes  No | | 1, 2 | | | 164 (41.9)  152 (41.1)  152  (42.5)  12  (50.0) | | | 218 (57.1)  218 (58.9)  206  (57.5)  12  (50.0) | | | 1 | | | 126 (49.0)  126 (49.0)  95  (51.6)  31 (42.5) | | 131 (51.0)  131 (51.0)  89  (48.4)  42  (57.5) | | no | | | 22  (7, 9, 6) | | |  |
| Katoh, 1995  (135) | Asian  -Japan | | |  | | 1, 2 | | | 18  (54.5) | | | 15  (45.5) | | | 1 | | | 53  (60.2) | | 35  (39.8) | | no | | | 13  (3, 5, 5) | | |  |
| Kihara, 1995  (136) | Asian  -Japan  Male | | | Yes  No | | 1b, 2 | | | 42  (43.3)  42  (43.3)  42  (43.3) | | | 55  (56.7)  55  (56.7)  55  (56.7) | | | 1 | | | 127 (49.2)  127 (49.2)  96 (51.9)  31 (42.5) | | 131 (50.8)  131 (50.8)  89  (48.1)  42  (57.5) | | no | | | 19  (7, 7, 5) | | |  |
| Kihara, 1995  (137) | Asian  -Japan  Male  Female | | | Yes  No | | 1, 2 | | | 197 (44.1)  150  (44.8)  47  (42.0)  141  (41.7)  52  (51.0) | | | 250 (55.9)  185  (55.2)  65  (58.0)  197  (58.3)  50  (49.0) | | | 1 | | | 241 (51.4)  150  (49.5)  91 (54.8)  120  (51.7)  121 (51.1) | | 228 (48.6)  153  (50.5)  75  (45.2)  112  (48.3)  116 (48.9) | | no | | | 17  (6, 5, 6) | | |  |
| Kihara, 1994  (138) | Asian  -Japan  Male  Female | | | Yes | | 1, 2 | | | 70  (39.3)  66  (42.0)  4  (19.0)  63  (43.2) | | | 108 (60.7)  91  (58.0)  17  (81.0)  83  (56.8) | | | 1 | | | 110 (54.7)  77  (55.0)  33  (54.1)  77 (55.0) | | 91  (45.3)  63  (45.0)  28  (45.9)  63  (45.0) | | no | | | 21  (8, 8, 5) | | |  |
| Nakachi, 1993 (139) | Asian  -Japan | | | Yes | | 1, 2 | | | 33  (38.8)  33  (38.8) | | | 52  (61.2)  52  (61.2) | | | 1 | | | 86  (50.6)  86  (50.6) | | 84  (49.4)  84  (49.4) | | yes | | | 20  (9, 5, 6) | | |  |
| Piao, 2013  (140) | Asian  -Korea  Male  Female | | |  | | 1, 2 | | | 1696 (43.1)  1331  (42.6)  365 (45.1) | | | 2237 (56.9)  1792  (57.4)  445 (54.9) | | | 1 | | | 776 (45.7)  369  (45.0)  407 (46.3) | | 923 (54.3)  451  (55.0)  472 (53.7) | | no | | | 20  (8, 6, 6) | | |  |
| Yang, 2007  (141) | Asian  -Korea | | |  | | 1a | | | 159 (50.2) | | | 158 (49.8) | | | 1 | | | 167 (48.3) | | 179 (51.7) | | no | | | 20  (6, 8, 6) | | |  |
| Lee, 2006  (142) | Asian  -Korea  Male | | |  | | 1, 2 | | | 71  (42.0)  71  (42.0) | | | 98  (58.0)  98  (58.0) | | | 2 | | | 91 (46.4)  91 (46.4) | | 105 (53.6)  105  (53.6) | | no | | | 25  (8, 11, 6) | | |  |
| Hong, 1998  (143) | Asian  -Korea | | |  | | 1, 2 | | | 38  (44.7) | | | 47  (55.3) | | | 2 | | | 30 (47.6) | | 33  (52.4) | | no | | | 14  (3, 5, 6) | | |  |
|  |  | | |  | |  | | |  | | |  | | |  | | |  | |  | |  | | |  | | |  |
| **East Asian** |  | | |  | |  | | |  | | |  | | |  | | |  | |  | |  | | |  | | |  |
| Chou, 2005  (144) | Asian  -Taiwan | | |  | | 1, 2 | | | 12  (40.0) | | | 18  (60.0) | | | 1 | | | 21  (35.0) | | 39  (65.0) | | no | | | 22  (7, 9, 6) | | |  |
| Cheng, 2000  (145) | Asian  -Taiwan | | |  | | 1 | | | 39  (53.4) | | | 34  (46.6) | | | 2 | | | 16 (48.5) | | 17  (51.5) | | no | | | 19  (7, 6, 6) | | |  |
| Jiang, 2014  (146) | Asian  -China | | | Yes  No | | 1, 2 | | | 132 (41.0)  65  (30.1)  48  (45.3) | | | 190 (59.0)  151  (69.9)  58  (54.7) | | | 1 | | | 268 (58.8)  115  (60.5)  130 (48.9) | | 188 (41.2)  75  (39.5)  136 (51.1) | | no | | | 18  (5, 7, 6) | | |  |
| Pan, 2014 (147) | Asian  -China  Male  Female | | | Yes  No | | 1 | | | 218 (41.7)  122  (43.0)  96  (37.2)  104  (43.5)  114  (40.1) | | | 305 (58.3)  143  (57.0)  162  (62.8)  135  (56.5)  170  (59.9) | | | 1 | | | 299 (57.2)  144  (54.3)  155  (60.1)  137  (57.3)  162  (57.0) | | 224 (42.8)  121  (45.6)  103  (39.9)  102  (42.7)  122  (43.0) | | no | | | 23  (7, 10, 6) | | |  |
| Zhang, 2014 (148) | Asian  -China | | |  | | 1 | | | 44  (40.0) | | | 66  (60.0) | | | 1 | | | 58  (58.0) | | 42  (42.0) | | no | | | 17  (7, 4, 6) | | |  |
| Chen, 2012 (149) | Asian  -China | | | Yes  No | | 1 | | | 77  (38.5)  57  (39.0)  20  (37.0) | | | 123  (61.5)  89  (61.0)  34  (63.0) | | | 1 | | | 79  (41.8)  50  (44.2)  29 (38.2) | | 110  (58.2)  63  (55.8)  47  (61.8) | | yes | | | 20  (6, 9, 5) | | |  |
| Liu, 2012 (150) | Asian  -China | | | Yes  No | | 1, 2 | | | 215 (59.7)  152  (59.6)  63  (60.0) | | | 145 (40.2)  103  (40.4)  42  (40.0) | | | 1 | | | 253 (70.3)  130  (70.3)  123 (70.3) | | 107 (29.7)  55  (29.7)  52  (29.7) | | yes | | | 22  (9, 7, 6) | | |  |
| Li, 2012 (151) | Asian  -China | | | Yes  No | | 1 | | | 90  (41.5)  49  (40.5)  41  (42.7) | | | 127 (58.5)  72  (59.5)  55  (57.3) | | | 1 | | | 105 (52.5)  40  (63.5)  65 (48.1) | | 95  (47.5)  23  (36.5)  70  (51.9) | | yes | | | 20  (6, 8, 6) | | |  |
| Fowke, 2011 (152) | Asian  -China  Female | | |  | | 1, 2 | | | 98  (47.1)  98  (47.1) | | | 110  (52.9)  110  (52.9) | | | 1 | | | 329  (41.9)  329  (41.9) | | 456  (58.1)  456  (58.1) | | yes | | | 22  (6, 10, 6) | | |  |
| Jin, 2011 (153) | Asian  -China | | |  | | 1 | | | 90  (58.4) | | | 64  (41.6) | | | 2 | | | 96 (62.3) | | 58  (37.7) | | yes | | | 21  (9, 6, 6) | | |  |
| Jin, 2010 (154) | Asian  -China | | | Yes  No | | 1 | | | 55  (36.7)  43  (38.1)  12  (32.4) | | | 95  (63.3)  70  (61.9)  25  (67.6) | | | 2 | | | 71 (47.3)  36  (41.4)  35 (55.6) | | 79  (52.7)  51  (58.6)  28  (44.4) | | yes | | | 19  (7, 6, 6) | | |  |
| Zheng, 2010  (155) | Asian  -China | | |  | | 1, 2 | | | 115  (43.4) | | | 150  (56.6) | | | 1 | | | 132  (43.0) | | 175  (57.0) | | no | | | 24  (7,11, 6) | | |  |
| Zhu, 2010  (156) | Asian  -China  Female | | |  | | 1, 2 | | | 67  (41.9)  67  (41.9) | | | 93  (58.1)  93  (58.1) | | | 1 | | | 88  (55.0)  88  (55.0) | | 72  (45.0)  72  (45.0) | | yes | | | 25  (8, 11, 6) | | |  |
| Chang, 2006  (157) | Asian  -China | | | Yes  No | | 1, 2 | | | 57  (35.0)  39  (38.6)  18  (29.0) | | | 106  (65.0)  62  (61.4)  44  (71.0) | | | 2 | | | 85  (52.1)  51  (76.1)  34 (35.4) | | 78  (47.9)  16  (23.9)  62  (64.6) | | no | | | 17  (5, 8, 4) | | |  |
| Chen, 2006  (158) | Asian  -China | | |  | | 1 | | | 37  (38.1) | | | 60  (61.9) | | | 1 | | | 108 (54.8) | | 89  (45.2) | | no | | | 16  (7, 4, 5) | | |  |
| Qian, 2006 (159) | Asian  -China | | | Yes  No | | 1, 2 | | | 39  (36.1)  31  (36.5)  8  (34.8) | | | 69  (63.9)  54  (63.5)  15  (65.2) | | | 1 | | | 55 (50.9)  31  (50.0)  24 (52.2) | | 53  (49.1)  31  (50.0)  22  (47.8) | | no | | | 19  (6, 7, 6) | | |  |
| Chan, 2005 (160) | Asian  -China  Male  Female | | |  | | 1 | | | 44  (58.7)  38  (61.3)  6  (46.2) | | | 31  (41.3)  24  (38.7)  7  (53.8) | | | 1 | | | 71 (43.8)  59  (42.4)  12 (52.2) | | 91  (56.2)  80  (57.6)  11  (47.8) | | no | | | 17  (6, 5, 6) | | |  |
| Li, 2005 (161) | Asian  -China | | | Yes  No | | 1 | | | 42  (42.4)  28  (44.4)  14  (38.9) | | | 57  (57.6)  35  (55.6)  22  (61.1) | | | 2 | | | 39 (59.1)  6  (37.5)  33 (66.0) | | 27  (40.9)  10  (62.5)  17  (34.0) | | no | | | 22  (6, 10, 6) | | |  |
| Li, 2005 (162) | Asian  -China | | | Yes  No | | 1, 2 | | | 40  (38.8)  33  (43.4)  7  (25.9) | | | 63  (61.2)  43  (56.6)  20  (74.1) | | | 1 | | | 77 (55.8)  41  (55.4)  36 (56.3) | | 61  (44.2)  33  (44.6)  28  (43.7) | | yes | | | 23  (7, 10, 6) | | |  |
| Wang, 2005 (163) | Asian  -China | | |  | | 1, 2 | | | 32  (41.6) | | | 45  (58.4) | | | 1 | | | 62 (57.9) | | 45  (42.1) | | no | | | 15  (4, 7, 4) | | |  |
| Ye, 2005 (164) | Asian  -China | | |  | | 1, 2 | | | 35  (60.3) | | | 23  (39.7) | | | 2 | | | 29 (46.8) | | 33  (53.2) | | no | | | 19  (5, 8, 6) | | |  |
| Zeng, 2005 (165) | Asian  -China | | |  | | 1, 2 | | | 35  (38.5) | | | 56  (61.5) | | | 2 | | | 40 (44.0) | | 51  (56.0) | | no | | | 21  (5, 10, 6) | | |  |
| Cao, 2004 (166) | Asian  -China | | |  | | 1, 2 | | | 39  (37.5) | | | 65  (62.5) | | | 1 | | | 110 (53.7) | | 95  (46.3) | | no | | | 16  (4, 7, 5) | | |  |
| Chen, 2004 (167) | Asian  -China | | |  | | 1, 2 | | | 35  (38.5) | | | 56  (61.5) | | | 1, 2 | | | 65 (47.1) | | 73  (52.9) | | no | | | 22  (7, 10, 5) | | |  |
| Dong, 2004 (168) | Asian  -China | | |  | | 1, 2 | | | 34  (41.5) | | | 48  (58.5) | | | 2 | | | 55 (60.4) | | 36  (39.6) | | no | | | 18  (4, 10, 4) | | |  |
| Gu, 2004 (169) | Asian  -China | | |  | | 1, 2 | | | 79  (43.9) | | | 101 (56.1) | | | 1, 2 | | | 122 (54.5) | | 102 (45.5) | | no | | | 20  (7, 8, 5) | | |  |
| Liang, 2004 (170) | Asian  -China | | |  | | 1 | | | 70  (46.1) | | | 82  (53.9) | | | 2 | | | 73 (48.0) | | 79  (52.0) | | no | | | 19  (6, 8, 5) | | |  |
| Luo, 2004 (171) | Asian  -China | | |  | | 1, 2 | | | 18  (28.6) | | | 45  (71.4) | | | 2 | | | 23 (48.9) | | 24  (51.1) | | no | | | 21  (6, 10, 5) | | |  |
| Wang, 2004 (172) | Asian  -China | | | Yes  No | | 1, 2 | | | 35  (38.5)  24  (43.6)  11  (30.6) | | | 56  (61.5)  31  (56.4)  25  (69.4) | | | 1, 2 | | | 65  (47.1)  28  (45.2)  37 (48.7) | | 73  (52.9)  34  (54.8)  39  (51.3) | | no | | | 17  (5, 8, 4) | | |  |
| Yang, 2004 (173) | Asian  -China | | |  | | 1, 2 | | | 78  (41.9) | | | 108 (58.1) | | | 1 | | | 64 (46.0) | | 75  (54.0) | | no | | | 18  (7, 5, 6) | | |  |
| Ye, 2004 (174) | Asian  -China | | |  | | 1, 2 | | | 23  (29.7) | | | 35  (60.3) | | | 2 | | | 33 (53.2) | | 29  (46.8) | | no | | | 21  (5, 10, 6) | | |  |
| Chan-Yeung, 2004 (175) | Asian  -China | | |  | | 1, 2 | | | 99  (43.2) | | | 130 (56.8) | | | 1 | | | 80 (40.6) | | 117 (59.4) | | no | | | 19  (8, 5, 6) | | |  |
| Chen, 2003 (176) | Asian  -China | | | Yes  No | | 1, 2 | | | 14  (36.8)  9  (36.0)  5  (38.5) | | | 24  (63.2)  16  (64.0)  8  (61.5) | | | 1 | | | 42 (42.4)  15  (41.7)  27 (42.9) | | 57  (57.6)  21  (58.3)  36  (57.1) | | no | | | 20  (7, 7, 6) | | |  |
| Wang, 2003  (177) | Asian  -China | | | Yes  No | | 1a | | | 67  (40.9)  26  (37.1)  41  (43.6) | | | 97  (59.1)  44  (62.9)  53  (56.4) | | | 1 | | | 91 (50.3)  38  (50.0)  53 (50.5) | | 90  (49.7)  38  (50.0)  52  (49.5) | | no | | | 20  (7, 7, 6) | | |  |
| Wang, 2003  (178) | Asian  -China | | | Yes  No | | 1a | | | 43  (38.4)  19  (39.6)  24  (37.5) | | | 69  (61.6)  29  (60.4)  40  (62.5) | | | 1 | | | 59 (49.6)  24  (50.0)  35 (49.3) | | 60  (50.4)  24  (50.0)  36  (50.7) | | yes | | | 14  (5, 3, 6) | | |  |
| Xian, 2003  (179) | Asian  -China | | | Yes  No | | 1, 2 | | | 35  (38.5)  24  (43.7)  11  (30.6) | | | 56  (61.5)  31  (56.3)  25  (69.4) | | | 1 | | | 65 (47.1)  28  (45.2)  37 (48.7) | | 73  (52.9)  34  (54.8)  39  (51.2) | | no | | | 21  (6, 10, 5) | | |  |
| Chan, 2002 (180) | Asian  -China | | |  | | 1, 2 | | | 13  (23.2) | | | 43  (76.8) | | | 1 | | | 34 (34.3) | | 65  (65.7) | | no | | | 12  (1, 7, 4) | | |  |
| Lu, 2002 (181) | Asian  -China | | | Yes  No | | 1 | | | 156 (49.7)  99  (48.8)  57  (51.4) | | | 158 (50.3)  104  (51.2)  54  (48.6) | | | 1 | | | 159 (50.6)  87  (52.7)  72 (48.3) | | 155 (46.4)  78  (47.3)  77  (51.7) | | no | | | 24  (8, 10, 6) | | |  |
| Shi, 2002 (182) | Asian  -China | | |  | | 1, 2 | | | 46  (38.3) | | | 74  (61.7) | | | 1 | | | 67 (55.8) | | 53  (44.2) | | no | | | 14  (6, 7, 1) | | |  |
| Zhang, 2002  (183) | Asian  -China | | | Yes  No | | 1, 2 | | | 24  (36.9)  18  (43.9)  6  (42.9) | | | 41  (63.1)  23  (56.1)  8  (57.1) | | | 2 | | | 33 (55.0)  19  (59.4)  14 (50.0) | | 27  (45.0)  13  (40.6)  14  (50.0) | | no | | | 17  (5, 8, 4) | | |  |
| Chen, 2001 (184) | Asian  -China | | | Yes | | 1 | | | 50  (47.2)  38  (47.5) | | | 56  (52.8)  42  (52.5) | | | 1 | | | 67 (63.2)  51 (63.8) | | 39  (36.8)  29  (36.2) | | no | | | 16  (7, 4, 5) | | |  |
| Xue, 2001 (185) | Asian  -China | | |  | | 1, 2 | | | 50  (47.2) | | | 56  (52.8) | | | 1, 2 | | | 67 (63.2) | | 39  (36.8) | | no | | | 16  (4, 7, 5) | | |  |
| Lan, 2000 (186) | Asian  -China | | |  | | 1, 2 | | | 40  (32.8) | | | 82  (67.2) | | | 1 | | | 62  (50.8) | | 60  (49.2) | | no | | | 22  (7, 9, 6) | | |  |
| London, 2000 (187) | Asian  -China | | |  | | 1, 2 | | | 110 (47.4) | | | 122 (52.6) | | | 1 | | | 283 (39.9) | | 427 (60.1) | | no | | | 19  (6, 8, 5) | | |  |
| Chen, 1999  (188) | Asian  -China | | |  | | 1, 2 | | | 29  (42.6) | | | 39  (57.4) | | | 1 | | | 63 (60.0) | | 42  (40.0) | | no | | | 12  (1, 7, 4) | | |  |
| Gao, 1999 (189) | Asian  -China | | | Yes  No | | 1 | | | 25  (42.4)  18  (47.4)  7  (33.3) | | | 34  (57.6)  20  (52.6)  14  (66.7) | | | 1, 2 | | | 67 (50.8)  28  (59.6)  39 (45.9) | | 65  (49.2)  19  (40.4)  46  (54.1) | | no | | | 18  (7, 6, 5) | | |  |
| Lan, 1999 (190) | Asian  -China | | |  | | 1, 2 | | | 30  (34.9) | | | 56  (65.1) | | | 1 | | | 48 (55.8) | | 38  (44.2) | | no | | | 16  (4, 8, 4) | | |  |
| Persson, 1999 (191) | Asian  -China | | |  | | 1, 2 | | | 27  (36.0) | | | 48  (64.0) | | | 1 | | | 40  (33.6) | | 79  (66.4) | | no | | | 12  (4, 4, 4) | | |  |
| Gao, 1998 (192) | Asian  -China | | |  | | 1, 2 | | | 19  (41.3) | | | 27  (58.7) | | | 1 | | | 45 (64.3) | | 25  (35.7) | | no | | | 17  (3, 9, 5) | | |  |
| Sun, 1997 (193) | Asian  -China  Male  Female | | |  | | 1, 2 | | | 60  (29.0)  38  (25.5)  22  (32.4) | | | 147  (71.0)  101  (74.5)  46  (67.6) | | | 2 | | | 178  (48.9)  129  (48.1)  49 (51.0) | | 186  (51.1)  139  (51.9)  47  (49.0) | | no | | | 14  (2, 7, 5) | | |  |
| Ge, 1996 (194) | Asian  -China | | |  | | 1 | | | 30  (33.7) | | | 59  (66.3) | | | 1, 2 | | | 18  (34.0) | | 35  (66.0) | | yes | | | 20  (7, 7, 6) | | |  |
|  | | | |  | |  | | |  | | |  | | |  | | |  | |  | |  | | |  | | |  |
| **South East Asian** | | | |  | |  | | |  | | |  | | |  | | |  | |  | |  | | |  | | |  |
| Klinchid, 2009 (195) | Asian  -Thailand | | |  | | 1 | | | 26  (29.9) | | | 61  (70.1) | | | 1 | | | 34 (42.0) | | 47  (58.0) | | yes | | | 18  (6, 6, 6) | | |  |
| Pisani, 2006  (196) | Asian  -Thailand | | |  | | 1, 2 | | | 67  (40.4) | | | 99  (59.6) | | | 1, 2 | | | 105 (36.3) | | 184 (63.7) | | no | | | 19  (7, 6, 6) | | |  |
| Zhao, 2001  (197) | Asian  -Singapore  Female | | | Yes  No | | 1, 2 | | | 87  (37.3)  87  (37.3)  35  (36.5)  52  (38.0) | | | 146 (62.7)  146 (62.7)  61  (63.5)  85  (62.0) | | | 2 | | | 68 (36.4)  68 (36.4)  8  (44.4)  60 (35.5) | | 119 (63.6)  119 (63.6)  10  (55.6)  109 (64.5) | | no | | | 20  (7, 8, 5) | | |  |
|  |  | | |  | |  | | |  | | |  | | |  | | |  | |  | |  | | |  | | |  |
| **South Asian** |  | | |  | |  | | |  | | |  | | |  | | |  | |  | |  | | |  | | |  |
| Girdhar, 2016  (198) | Asian  -India | | |  | | 1, 2 | | | 160  (50.0) | | | 198  (61.9) | | | 1 | | | 160  (50.0) | | 122  (38.1) | | yes | | | 14  (3, 5, 6) | | |  |
| Peddireddy, 2016 (199) | Asian  -India | | | Yes  No | | 1 | | | 182  (74.0)  106  (71.6)  76  (77.6) | | | 64  (26.0)  42  (28.4)  22  (22.4) | | | 1 | | | 187  (76.0)  54  (72.0)  133  (76.0) | | 63  (24.0)  21  (28.0)  42  (24.0) | | no | | | 24  (7, 11, 6) | | |  |
| Sharma, 2015 (200) | Asian  -India | | |  | | 1, 2 | | | 136  (50.4) | | | 134  (49.6) | | | 1 | | | 167  (61.9) | | 103  (38.1) | | yes | | | 19  (8, 5, 6) | | |  |
| Ihsan, 2014  (201) | Asian  -India | | | Yes | | 1, 2 | | | 97  (63.0)  68  (64.8) | | | 57  (37.0)  37  (35.2) | | | 1 | | | 88 (57.1)  40 (56.3) | | 66  (42.9)  31  (43.7) | | yes | | | 22  (8, 8, 6) | | |  |
| Phukan, 2014 (202) | Asian  -India  Female | | |  | | 1, 2 | | | 114 (49.6)  114 (49.6) | | | 116 (50.4)  116 (50.4) | | | 1 | | | 292 (63.5)  292 (63.5) | | 168 (36.5) 168 (36.5) | | no | | | 21  (7, 9, 5) | | |  |
| Shukla, 2013 (203) | Asian  -India | | | Yes  No | | 1, 2 | | | 134 (61.5)  102  (68.5)  32  (46.4) | | | 84  (38.5)  47  (31.5)  37  (53.6) | | | 1 | | | 148 (62.2)  42  (61.8)  106 (62.4) | | 90  (37.8)  26  (38.2)  64  (37.6) | | no | | | 21  (6, 10, 5) | | |  |
| Ihsan, 2011 (204) | Asian  -India | | | Yes  No | | 1, 2 | | | 122 (64.9)  91  (68.9)  31  (55.4) | | | 66  (35.1)  41  (31.1)  25  (44.6) | | | 1 | | | 177 (61.0)  86  (61.9)  91 (60.3) | | 113 (39.0)  53  (38.1)  60  (39.7) | | yes | | | 20  (7, 7, 6) | | |  |
| Kumar, 2009 (205) | Asian  -India | | | Yes  No | | 1, 2 | | | 49  (52.7)  42  (51.9)  7  (58.3) | | | 44  (47.3)  39  (48.1)  5  (41.7) | | | 1 | | | 154 (60.9)  65  (64.4)  89 (58.6) | | 99  (39.1)  36  (35.6)  63  (41.4) | | no | | | 11  (3, 4, 4) | | |  |
| Shah, 2008  (206) | Asian  -India  Male | | |  | | 1b | | | 111 (55.5)  111 (55.5) | | | 89  (44.5)  89  (44.5) | | | 1 | | | 152 (76.0)  152 (76.0) | | 48  (24.0)  48  (24.0) | | no | | | 18  (7, 5, 6) | | |  |
| Sobti, 2008 (207) | Asian  -India | | |  | | 1, 2 | | | 88  (58.3) | | | 63  (41.7) | | | 2 | | | 98 (64.9) | | 53  (35.1) | | no | | | 16  (6, 4, 6) | | |  |
| Sreeja, 2008 (208) | Asian  -India | | |  | | 1, 2 | | | 138 (65.4) | | | 73  (34.6) | | | 1, 2 | | | 147 (69.7) | | 64  (30.3) | | no | | | 19  (6, 7, 6) | | |  |
| Sreeja, 2005 (209) | Asian  -India | | | Yes  No | | 1, 2 | | | 100 (42.3)  83  (69.7)  17  (63.0) | | | 46  (31.5)  36  (30.3)  10  (37.0) | | | 1 | | | 107 (73.3)  61  (71.8)  46 (75.4) | | 39  (26.7)  24  (28.2)  15  (24.6) | | no | | | 22  (7, 9, 6) | | |  |
| Masood, 2016  (210) | Asian  -Pakistan  Male  Female | | | Yes  No | | 1, 2 | | | 199  (78.9)  156  (84.8)  64  (94.1)  117  (89.3)  109  (90.1) | | | 53  (21.0)  28  (15.2)  4  (5.9)  14  (10.7)  12  (9.9) | | | 1 | | | 212  (78.5)  169  (85.8)  68  (93.2)  70  (89.7)  171  (89.1) | | 58  (21.5)  28  (14.2)  5  (6.8)  8  (10.3)  21  (10.9) | | no | | | 17  (7, 4, 6) | | |  |

Note: HWE = Hardy Weinberg Equilibrium;

Cancer Site: 1 = Non-small cell lung cancer (NSCLC) (n=32, unique group); 1a: adenocarcinoma (n=4), 1b: squamous cell carcinoma (n=2 unique group); 2 = small cell lung cancer (SCLC) (n=131 mixed groups of 1 and 2);

Sources of Controls: 1 = population-based healthy adults (n=117 unique group); 2 = hospital adults with other health conditions (n=42 unique group), 1,2 = mixed control groups (n=10);

Quality Score ranges = 0-30: external validity = 0-11, Internal Validity = 0-12, report quality = 0-7;

^a^6 European Countries: Slovakia, Poland, Czech Republic, Hungary, Romania, Russia;

United Kingdom (UK); US = United States of America.

**Supplementary References Included in the Meta-Analysis**

**33 pooled and meta-analysis papers:**

1. Gao Y, Gao F, Hu TT, Li G, Sui YX. Combined effects of glutathione S-transferase M1 and T1 polymorphisms on risk of lung cancer: evidence from a meta-analysis. Oncotarget. 2017; 8:28135–43.<https://doi.org/10.18632/oncotarget.15943>

2. Ramos Hernández C, Mouronte-Roibás C, Barros-Dios JM, Fernández-Villar A, Ruano-Ravina A. Deletion of GSTM1 and GSTT1 genes and lung cancer survival: a systematic review. Tumori. 2017; 103:338–44.<https://doi.org/10.5301/tj.5000621>

3. Yang H, Yang S, Liu J, Shao F, Wang H, Wang Y. The association of GSTM1 deletion polymorphism with lung cancer risk in Chinese population: evidence from an updated meta-analysis. Sci Rep. 2015; 5:9392.<https://doi.org/10.1038/srep09392>

4. Liu H, Ma HF, Chen YK. Association between GSTM1 polymorphisms and lung cancer: an updated meta-analysis. Genet Mol Res. 2015; 14:1385–92.<https://doi.org/10.4238/2015.February.13.17>

5. Chen XP, Xu WH, Xu DF, Xie XH, Yao J, Fu SM. GSTM1 polymorphisms and lung cancer risk in the Chinese population: a meta-analysis based on 47 studies. Asian Pac J Cancer Prev. 2014; 15:7741–46.<https://doi.org/10.7314/APJCP.2014.15.18.7741>

6. Li W, Song LQ, Tan J. Combined effects of CYP1A1 MspI and GSTM1 genetic polymorphisms on risk of lung cancer: an updated meta-analysis. Tumour Biol. 2014; 35:9281–90.<https://doi.org/10.1007/s13277-014-2212-6>

7. Liu K, Lin X, Zhou Q, Ma T, Han L, Mao G, Chen J, Yue X, Wang H, Zhang L, Jin G, Jiang J, Zhao J, Zou B. The associations between two vital GSTs genetic polymorphisms and lung cancer risk in the Chinese population: evidence from 71 studies. PLoS One. 2014; 9:e102372.<https://doi.org/10.1371/journal.pone.0102372>

8. Liu X, Li Z, Zhang Z, Zhang W, Li W, Xiao Z, Liu H, Jiao H, Wang Y, Li G. Meta-analysis of GSTM1 null genotype and lung cancer risk in Asians. Med Sci Monit. 2014; 20:1239–45.<https://doi.org/10.12659/MSM.890490>

9. Yang Y, Xian L. The association between the GSTP1 A313G and GSTM1 null/present polymorphisms and the treatment response of the platinum-based chemotherapy in non-small cell lung cancer (NSCLC) patients: a meta-analysis. Tumour Biol. 2014; 35:6791–99.<https://doi.org/10.1007/s13277-014-1866-4>

10. Zhao Y, Zeng J, Zhang Y, Lu S, Zhao E, Huang Z, Lu W. GSTM1 polymorphism and lung cancer risk among East Asian populations: a meta-analysis. Tumour Biol. 2014; 35:6493–500.<https://doi.org/10.1007/s13277-014-1832-1>

11. Horgan AM, Yang B, Azad AK, Amir E, John T, Cescon DW, Wheatley-Price P, Hung RJ, Shepherd FA, Liu G. Pharmacogenetic and germline prognostic markers of lung cancer. J Thorac Oncol. 2011; 6:296–304.<https://doi.org/10.1097/JTO.0b013e3181ffe909>

12. Li C, Yin Z, Zhou B. [CYP1A1 gene and GSTM1 gene polymorphism and the combined effects and risk of lung cancer: a meta-analysis]. [Article in Chinese]. Zhongguo Fei Ai Za Zhi. 2011; 14:660–68.<https://doi.org/10.3779/j.issn.1009-3419.2011.08.05>

13. Langevin SM, Ioannidis JP, Vineis P, Taioli E, and Genetic Susceptibility to Environmental Carcinogens group (GSEC). Assessment of cumulative evidence for the association between glutathione S-transferase polymorphisms and lung cancer: application of the Venice interim guidelines. Pharmacogenet Genomics. 2010; 20:586–97.<https://doi.org/10.1097/FPC.0b013e32833c3892>

14. Okazaki I, Sugita M, Matsuki H, Billah SM, Watanabe T. Additional candidates to conventional genes susceptible for lung cancer and changing trend in Japan. Oncol Rep. 2010; 23:1493–500.<https://doi.org/10.3892/or_00000788>

15. Ragin CC, Langevin S, Rubin S, Taioli E. Review of studies on metabolic genes and cancer in populations of African descent. Genet Med. 2010; 12:12–18.<https://doi.org/10.1097/GIM.0b013e3181c8e160>

16. Lam TK, Gallicchio L, Lindsley K, Shiels M, Hammond E, Tao XG, Chen L, Robinson KA, Caulfield LE, Herman JG, Guallar E, Alberg AJ. Cruciferous vegetable consumption and lung cancer risk: a systematic review. Cancer Epidemiol Biomarkers Prev. 2009; 18:184–95.<https://doi.org/10.1158/1055-9965.EPI-08-0710>

17. Carlsten C, Sagoo GS, Frodsham AJ, Burke W, Higgins JP. Glutathione S-transferase M1 (GSTM1) polymorphisms and lung cancer: a literature-based systematic HuGE review and meta-analysis. Am J Epidemiol. 2008; 167:759–74.<https://doi.org/10.1093/aje/kwm383>

18. Neri M, Ugolini D, Dianzani I, Gemignani F, Landi S, Cesario A, Magnani C, Mutti L, Puntoni R, Bonassi S. Genetic susceptibility to malignant pleural mesothelioma and other asbestos-associated diseases. Mutat Res. 2008; 659:126–36.<https://doi.org/10.1016/j.mrrev.2008.02.002>

19. Shi X, Zhou S, Wang Z, Zhou Z, Wang Z. CYP1A1 and GSTM1 polymorphisms and lung cancer risk in Chinese populations: a meta-analysis. Lung Cancer. 2008; 59:155–63.<https://doi.org/10.1016/j.lungcan.2007.08.004>

20. Hosgood HD 3rd, Berndt SI, Lan Q. GST genotypes and lung cancer susceptibility in Asian populations with indoor air pollution exposures: a meta-analysis. Mutat Res. 2007; 636:134–43.<https://doi.org/10.1016/j.mrrev.2007.02.002>

21. Ye Z, Song H, Higgins JP, Pharoah P, Danesh J. Five glutathione s-transferase gene variants in 23,452 cases of lung cancer and 30,397 controls: meta-analysis of 130 studies. PLoS Med. 2006; 3:e91.<https://doi.org/10.1371/journal.pmed.0030091>

22. Raimondi S, Boffetta P, Anttila S, Bröckmoller J, Butkiewicz D, Cascorbi I, Clapper ML, Dragani TA, Garte S, Gsur A, Haidinger G, Hirvonen A, Ingelman-Sundberg M, et al. Metabolic gene polymorphisms and lung cancer risk in non-smokers. An update of the GSEC study. Mutat Res. 2005; 592:45–57.<https://doi.org/10.1016/j.mrfmmm.2005.06.002>

23. Seow A, Vainio H, Yu MC. Effect of glutathione-S-transferase polymorphisms on the cancer preventive potential of isothiocyanates: an epidemiological perspective. Mutat Res. 2005; 592:58–67.<https://doi.org/10.1016/j.mrfmmm.2005.06.004>

24. Habdous M, Siest G, Herbeth B, Vincent-Viry M, Visvikis S. [Glutathione S-transferases genetic polymorphisms and human diseases: overview of epidemiological studies]. [Article in French]. Ann Biol Clin (Paris). 2004; 62:15–24.

25. Mohr LC, Rodgers JK, Silvestri GA. Glutathione S-transferase M1 polymorphism and the risk of lung cancer. Anticancer Res. 2003; 23:2111–24.

26. Benhamou S, Lee WJ, Alexandrie AK, Boffetta P, Bouchardy C, Butkiewicz D, Brockmöller J, Clapper ML, Daly A, Dolzan V, Ford J, Gaspari L, Haugen A, et al. Meta- and pooled analyses of the effects of glutathione S-transferase M1 polymorphisms and smoking on lung cancer risk. Carcinogenesis. 2002; 23:1343–50. Erratum in: Carcinogenesis 2002; 23:1771.

27. Houlston RS. Glutathione S-transferase M1 status and lung cancer risk: a meta-analysis. Cancer Epidemiol Biomarkers Prev. 1999; 8:675–82.

28. Hengstler JG, Arand M, Herrero ME, Oesch F. Polymorphisms of N-acetyltransferases, glutathione S-transferases, microsomal epoxide hydrolase and sulfotransferases: influence on cancer susceptibility. Recent Results Cancer Res. 1998; 154:47–85.<https://doi.org/10.1007/978-3-642-46870-4_4>

29. Hung RJ, Boffetta P, Brockmöller J, Butkiewicz D, Cascorbi I, Clapper ML, Garte S, Haugen A, Hirvonen A, Anttila S, Kalina I, Le Marchand L, London SJ, et al. CYP1A1 and GSTM1 genetic polymorphisms and lung cancer risk in Caucasian non-smokers: a pooled analysis. Carcinogenesis. 2003; 24:875–82.<https://doi.org/10.1093/carcin/bgg026>

30. Lee KM, Kang D, Clapper ML, Ingelman-Sundberg M, Ono-Kihara M, Kiyohara C, Min S, Lan Q, Le Marchand L, Lin P, Lung ML, Pinarbasi H, Pisani P, et al. CYP1A1, GSTM1, and GSTT1 polymorphisms, smoking, and lung cancer risk in a pooled analysis among Asian populations. Cancer Epidemiol Biomarkers Prev. 2008; 17:1120–26.<https://doi.org/10.1158/1055-9965.EPI-07-2786>

31. Vineis P, Anttila S, Benhamou S, Spinola M, Hirvonen A, Kiyohara C, Garte SJ, Puntoni R, Rannug A, Strange RC, Taioli E. Evidence of gene gene interactions in lung carcinogenesis in a large pooled analysis. Carcinogenesis. 2007; 28:1902–05.<https://doi.org/10.1093/carcin/bgm039>

32. Stücker I, Boffetta P, Antilla S, Benhamou S, Hirvonen A, London S, Taioli E. Lack of interaction between asbestos exposure and glutathione S-transferase M1 and T1 genotypes in lung carcinogenesis. Cancer Epidemiol Biomarkers Prev. 2001; 10:1253–58.

33. McWilliams JE, Sanderson BJ, Harris EL, Richert-Boe KE, Henner WD. Glutathione S-transferase M1 (GSTM1) deficiency and lung cancer risk. Cancer Epidemiol Biomarkers Prev. 1995; 4:589–94.

**14 papers with subsidiary or duplicate use of data on genotype allele counts:**

34. Kant Shukla R, Kant S, Mittal B, Bhattacharya S. Comparative study of GST polymorphism in relation to age in COPD and lung cancer. Tuberk Toraks. 2013; 61:275–82.<https://doi.org/10.5578/tt.6252>

35. Sobti RC, Sharma S, Joshi A, Jindal SK, Janmeja A. Genetic polymorphism of the CYP1A1, CYP2E1, GSTM1 and GSTT1 genes and lung cancer susceptibility in a north indian population. Mol Cell Biochem. 2004; 266:1–9.<https://doi.org/10.1023/B:MCBI.0000049127.33458.87>

36. Shah PP, Singh AP, Singh M, Mathur N, Pant MC, Mishra BN, Parmar D. Interaction of cytochrome P4501A1 genotypes with other risk factors and susceptibility to lung cancer. Mutat Res. 2008; 639:1–10.<https://doi.org/10.1016/j.mrfmmm.2007.10.006>

37. Belogubova EV, Ulibina YM, Suvorova IK, Kuligina ES, Karpova MB, Shutkin VA, Koloskov AV, Kuchinskiy AP, Togo AV, Hanson KP, Hirvonen A, Imyanitov EN. Combined CYP1A1/GSTM1 at-risk genotypes are overrepresented in squamous cell lung carcinoma patients but underrepresented in elderly tumor-free subjects. J Cancer Res Clin Oncol. 2006; 132:327–31.<https://doi.org/10.1007/s00432-005-0071-7>

38. Adonis M, Martínez V, Marín P, Gil L. CYP1A1 and GSTM1 genetic polymorphisms in lung cancer populations exposed to arsenic in drinking water. Xenobiotica. 2005; 35:519–30.<https://doi.org/10.1080/00498250500057310>

39. Sørensen M, Autrup H, Tjønneland A, Overvad K, Raaschou-Nielsen O. Glutathione S-transferase T1 null-genotype is associated with an increased risk of lung cancer. Int J Cancer. 2004; 110:219–24.<https://doi.org/10.1002/ijc.20075>

40. Lan Q, He X. Molecular epidemiological studies on the relationship between indoor coal burning and lung cancer in Xuan Wei, China. Toxicology. 2004; 198:301–05.<https://doi.org/10.1016/j.tox.2004.02.006>

41. Harms C, Salama SA, Sierra-Torres CH, Cajas-Salazar N, Au WW. Polymorphisms in DNA repair genes, chromosome aberrations, and lung cancer. Environ Mol Mutagen. 2004; 44:74–82.<https://doi.org/10.1002/em.20031>

42. Li WY, Lai BT, Zhan XP. [The relationship between genetic polymorphism of metabolizing enzymes and the genetic susceptibility to lung cancer]. [Article in Chinese]. Zhonghua Liu Xing Bing Xue Za Zhi. 2004; 25:1042–45.

43. London SJ, Yuan JM, Coetzee GA, Gao YT, Ross RK, Yu MC. CYP1A1 I462V genetic polymorphism and lung cancer risk in a cohort of men in Shanghai, China. Cancer Epidemiol Biomarkers Prev. 2000; 9:987–91.

44. London SJ, Smart J, Daly AK. Lung cancer risk in relation to genetic polymorphisms of microsomal epoxide hydrolase among African-Americans and Caucasians in Los Angeles County. Lung Cancer. 2000; 28:147–55.<https://doi.org/10.1016/S0169-5002(99)00130-0>

45. Saarikoski ST, Reinikainen M, Anttila S, Karjalainen A, Vainio H, Husgafvel-Pursiainen K, Hirvonen A. Role of NAT2 deficiency in susceptibility to lung cancer among asbestos-exposed individuals. Pharmacogenetics. 2000; 10:183–85.<https://doi.org/10.1097/00008571-200003000-00010>

46. Stücker I, de Waziers I, Cenée S, Bignon J, Depierre A, Milleron B, Beaune P, Hémon D. GSTM1, smoking and lung cancer: a case-control study. Int J Epidemiol. 1999; 28:829–35.<https://doi.org/10.1093/ije/28.5.829>

47. el-Zein R, Conforti-Froes N, Au WW. Interactions between genetic predisposition and environmental toxicants for development of lung cancer. Environ Mol Mutagen. 1997; 30:196–204. [https://doi.org/10.1002/(SICI)1098-2280(1997)30:2<196::AID-EM12>3.0.CO;2-8](https://doi.org/10.1002/(SICI)1098-2280(1997)30:2%3C196::AID-EM12%3E3.0.CO;2-8)

**163 papers included with genotype allele counts:**

48. Young RP, Hopkins RJ, Hay BA, Gamble GD. GSTM1 null genotype in COPD and lung cancer: evidence of a modifier or confounding effect? Appl Clin Genet. 2011; 4:137–44.<https://doi.org/10.2147/TACG.S21517>

49. Larsen JE, Colosimo ML, Yang IA, Bowman R, Zimmerman PV, Fong KM. CYP1A1 Ile462Val and MPO G-463A interact to increase risk of adenocarcinoma but not squamous cell carcinoma of the lung. Carcinogenesis. 2006; 27:525–32.<https://doi.org/10.1093/carcin/bgi227>

50. Belogubova EV, Togo AV, Karpova MB, Kuligina ES, Buslova KG, Ulibina JM, Lemehov VG, Romanenko SM, Shutkin VA, Hanson KP, Hirvonen A, Imyanitov EN. A novel approach for assessment of cancer predisposing roles of GSTM1 and GSTT1 genes: use of putatively cancer resistant elderly tumor-free smokers as the referents. Lung Cancer. 2004; 43:259–66.<https://doi.org/10.1016/j.lungcan.2003.08.019>

51. Woodson K, Stewart C, Barrett M, Bhat NK, Virtamo J, Taylor PR, Albanes D. Effect of vitamin intervention on the relationship between GSTM1, smoking, and lung cancer risk among male smokers. Cancer Epidemiol Biomarkers Prev. 1999; 8:965–70.

52. Saarikoski ST, Voho A, Reinikainen M, Anttila S, Karjalainen A, Malaveille C, Vainio H, Husgafvel-Pursiainen K, Hirvonen A. Combined effect of polymorphic GST genes on individual susceptibility to lung cancer. Int J Cancer. 1998; 77:516–21. [https://doi.org/10.1002/(SICI)1097-0215(19980812)77:4<516::AID-IJC7>3.0.CO;2-X](https://doi.org/10.1002/(SICI)1097-0215(19980812)77:4%3C516::AID-IJC7%3E3.0.CO;2-X)

53. Hirvonen A, Husgafvel-Pursiainen K, Anttila S, Vainio H. The GSTM1 null genotype as a potential risk modifier for squamous cell carcinoma of the lung. Carcinogenesis. 1993; 14:1479–81.<https://doi.org/10.1093/carcin/14.7.1479>

54. Alexandrie AK, Nyberg F, Warholm M, Rannug A. Influence of CYP1A1, GSTM1, GSTT1, and NQO1 genotypes and cumulative smoking dose on lung cancer risk in a Swedish population. Cancer Epidemiol Biomarkers Prev. 2004; 13:908–14.

55. Hou SM, Fält S, Yang K, Nyberg F, Pershagen G, Hemminki K, Lambert B. Differential interactions between GSTM1 and NAT2 genotypes on aromatic DNA adduct level and HPRT mutant frequency in lung cancer patients and population controls. Cancer Epidemiol Biomarkers Prev. 2001; 10:133–40.

56. Nyberg F, Hou SM, Hemminki K, Lambert B, Pershagen G. Glutathione S-transferase mu1 and N-acetyltransferase 2 genetic polymorphisms and exposure to tobacco smoke in nonsmoking and smoking lung cancer patients and population controls. Cancer Epidemiol Biomarkers Prev. 1998; 7:875–83.

57. Alexandrie AK, Sundberg MI, Seidegård J, Tornling G, Rannug A. Genetic susceptibility to lung cancer with special emphasis on CYP1A1 and GSTM1: a study on host factors in relation to age at onset, gender and histological cancer types. Carcinogenesis. 1994; 15:1785–90.<https://doi.org/10.1093/carcin/15.9.1785>

58. Ryberg D, Skaug V, Hewer A, Phillips DH, Harries LW, Wolf CR, Ogreid D, Ulvik A, Vu P, Haugen A. Genotypes of glutathione transferase M1 and P1 and their significance for lung DNA adduct levels and cancer risk. Carcinogenesis. 1997; 18:1285–89.<https://doi.org/10.1093/carcin/18.7.1285>

59. Sørensen M, Raaschou-Nielsen O, Brasch-Andersen C, Tjønneland A, Overvad K, Autrup H. Interactions between GSTM1, GSTT1 and GSTP1 polymorphisms and smoking and intake of fruit and vegetables in relation to lung cancer. Lung Cancer. 2007; 55:137–44.<https://doi.org/10.1016/j.lungcan.2006.10.010>

60. Reszka E, Wasowicz W, Gromadzinska J. Antioxidant defense markers modulated by glutathione S-transferase genetic polymorphism: results of lung cancer case-control study. Genes Nutr. 2007; 2:287–94.<https://doi.org/10.1007/s12263-007-0057-y>

61. Brennan P, Hsu CC, Moullan N, Szeszenia-Dabrowska N, Lissowska J, Zaridze D, Rudnai P, Fabianova E, Mates D, Bencko V, Foretova L, Janout V, Gemignani F, et al. Effect of cruciferous vegetables on lung cancer in patients stratified by genetic status: a mendelian randomisation approach. Lancet. 2005; 366:1558–60.<https://doi.org/10.1016/S0140-6736(05)67628-3>

62. Dzian A, Halasova E, Matakova T, Kavcova E, Smolar M, Dobrota D, Hamzik J, Mistuna D. Lung adenocarcinoma and squamous cell carcinoma in association with genetic polymorphisms of GSTs in Slovak population. Neoplasma. 2012; 59:160–67.<https://doi.org/10.4149/neo_2012_021>

63. Matakova T, Sivonova M, Halasova E, Mistuna D, Dzian A, Berzinec P, Letkova L, Dobrota D. Gene polymorphisms of biotransforming enzymes (GSTs) and their association with lung cancer in the Slovakian population. Eur J Med Res. 2009 (Suppl 4); 14:275–79.<https://doi.org/10.1186/2047-783X-14-S4-275>

64. Habalová V, Salagovic J, Kalina I, Stubna J. Combined analysis of polymorphisms in glutathione S-transferase M1 and microsomal epoxide hydrolase in lung cancer patients. Neoplasma. 2004; 51:352–57.

65. Salagovic J, Kalina I, Stubna J, Habalová V, Hrivnák M, Valanský L, Kohút A, Biros E. Genetic polymorphism of glutathione S-transferases M1 and T1 as a risk factor in lung and bladder cancers. Neoplasma. 1998; 45:312–17.

66. Timofeeva M, Kropp S, Sauter W, Beckmann L, Rosenberger A, Illig T, Jäger B, Mittelstrass K, Dienemann H, Bartsch H, Bickeböller H, Chang-Claude J, Risch A, Wichmann HE, and LUCY-Consortium. Genetic polymorphisms of MPO, GSTT1, GSTM1, GSTP1, EPHX1 and NQO1 as risk factors of early-onset lung cancer. Int J Cancer. 2010; 127:1547–61.<https://doi.org/10.1002/ijc.25175>

67. Schneider J, Bernges U, Philipp M, Woitowitz HJ. GSTM1, GSTT1, and GSTP1 polymorphism and lung cancer risk in relation to tobacco smoking. Cancer Lett. 2004; 208:65–74.<https://doi.org/10.1016/j.canlet.2004.01.002>

68. Risch A, Wikman H, Thiel S, Schmezer P, Edler L, Drings P, Dienemann H, Kayser K, Schulz V, Spiegelhalder B, Bartsch H. Glutathione-S-transferase M1, M3, T1 and P1 polymorphisms and susceptibility to non-small-cell lung cancer subtypes and hamartomas. Pharmacogenetics. 2001; 11:757–64.<https://doi.org/10.1097/00008571-200112000-00003>

69. Malats N, Camus-Radon AM, Nyberg F, Ahrens W, Constantinescu V, Mukeria A, Benhamou S, Batura-Gabryel H, Bruske-Hohlfeld I, Simonato L, Menezes A, Lea S, Lang M, Boffetta P. Lung cancer risk in nonsmokers and GSTM1 and GSTT1 genetic polymorphism. Cancer Epidemiol Biomarkers Prev. 2000; 9:827–33.

70. Brockmöller J, Kerb R, Drakoulis N, Nitz M, Roots I. Genotype and phenotype of glutathione S-transferase class mu isoenzymes mu and psi in lung cancer patients and controls. Cancer Res. 1993; 53:1004–11.

71. Stücker I, Hirvonen A, de Waziers I, Cabelguenne A, Mitrunen K, Cénée S, Koum-Besson E, Hémon D, Beaune P, Loriot MA. Genetic polymorphisms of glutathione S-transferases as modulators of lung cancer susceptibility. Carcinogenesis. 2002; 23:1475–81.<https://doi.org/10.1093/carcin/23.9.1475>

72. Benhamou Anu Voho Christine Bouchardy Katja Mitrunen Pierre Dayer Ari Hirvonen S. Role of NAD(P)H:quinone oxidoreductase polymorphism at codon 187 in susceptibility to lung, laryngeal and oral/pharyngeal cancers. Biomarkers. 2001; 6:440–47.<https://doi.org/10.1080/13547500110057399>

73. Lewis S, Brennan P, Nyberg F, Ahrens W, Constantinescu V, Mukeria A, Benhamou S, Batura-Gabryel H, Brüske-Hohlfeld I, Simonato L, Menezes A, Boffetta P. Re: Spitz, M. R., Duphorne, C. M., Detry, M. A., Pillow, P. C., Amos, C. I., Lei, L., de Andrade, M., Gu, X., Hong, W. K., and Wu, X. Dietary intake of isothiocyanates: evidence of a joint effect with glutathione S-transferase polymorphisms in lung cancer risk. Cancer Epidemiol. Biomark. Prev., 9: 1017-1020, 2000. Cancer Epidemiol Biomarkers Prev. 2001; 10:1105–06.

74. Jourenkova-Mironova N, Wikman H, Bouchardy C, Voho A, Dayer P, Benhamou S, Hirvonen A. Role of glutathione S-transferase GSTM1, GSTM3, GSTP1 and GSTT1 genotypes in modulating susceptibility to smoking-related lung cancer. Pharmacogenetics. 1998; 8:495–502.<https://doi.org/10.1097/00008571-199812000-00006>

75. Lewis SJ, Cherry NM, Niven RM, Barber PV, Povey AC. GSTM1, GSTT1 and GSTP1 polymorphisms and lung cancer risk. Cancer Lett. 2002; 180:165–71.<https://doi.org/10.1016/S0304-3835(02)00028-9>

76. Harrison DJ, Cantlay AM, Rae F, Lamb D, Smith CA. Frequency of glutathione S-transferase M1 deletion in smokers with emphysema and lung cancer. Hum Exp Toxicol. 1997; 16:356–60.<https://doi.org/10.1177/096032719701600703>

77. Deakin M, Elder J, Hendrickse C, Peckham D, Baldwin D, Pantin C, Wild N, Leopard P, Bell DA, Jones P, Duncan H, Brannigan K, Alldersea J, et al. Glutathione S-transferase GSTT1 genotypes and susceptibility to cancer: studies of interactions with GSTM1 in lung, oral, gastric and colorectal cancers. Carcinogenesis. 1996; 17:881–84.<https://doi.org/10.1093/carcin/17.4.881>

78. Zhong S, Howie AF, Ketterer B, Taylor J, Hayes JD, Beckett GJ, Wathen CG, Wolf CR, Spurr NK. Glutathione S-transferase mu locus: use of genotyping and phenotyping assays to assess association with lung cancer susceptibility. Carcinogenesis. 1991; 12:1533–37.<https://doi.org/10.1093/carcin/12.9.1533>

79. Ada AO, Kunak SC, Hancer F, Soydas E, Alpar S, Gulhan M, Iscan M. Association between GSTM1, GSTT1, and GSTP1 polymorphisms and lung cancer risk in a Turkish population. Mol Biol Rep. 2012; 39:5985–93.<https://doi.org/10.1007/s11033-011-1411-0>

80. Atinkaya C, Taspinar M, Sakiragaoglu O, Oz G, Yazici U, Oztuna D, Tastepe I, Sunguroglu A. The effect of CYP1A1, GSTT1 and GSTM1 polymorphisms on the risk of lung cancer: a case-control study. Hum Exp Toxicol. 2012; 31:1074–80.<https://doi.org/10.1177/0960327111428630>

81. Altinisik J, Balta ZB, Aydin G, Ulutin T, Buyru N. Investigation of glutathione S-transferase M1 and T1 deletions in lung cancer. Mol Biol Rep. 2010; 37:263–67.<https://doi.org/10.1007/s11033-009-9673-5>

82. Demir A, Altin S, Pehlivan D, Demir M, Yakar F, Seyhan EC, Dincer SI. The role of GSTM1 gene polymorphisms in lung cancer development in Turkish population. J Carcinog. 2007; 6:13.<https://doi.org/10.1186/1477-3163-6-13>

83. Pinarbasi H, Silig Y, Cetinkaya O, Seyfikli Z, Pinarbasi E. Strong association between the GSTM1-null genotype and lung cancer in a Turkish population. Cancer Genet Cytogenet. 2003; 146:125–29.<https://doi.org/10.1016/S0165-4608(03)00059-1>

84. Oztürk O, Isbir T, Yaylim I, Kocatürk CI, Gürses A. GST M1 and CYP1A1 gene polymorphism and daily fruit consumption in Turkish patients with non-small cell lung carcinomas. In Vivo. 2003; 17:625–32.

85. Pliarchopoulou K, Voutsinas G, Papaxoinis G, Florou K, Skondra M, Kostaki K, Roussou P, Syrigos K, Pectasides D. Correlation of CYP1A1, GSTP1 and GSTM1 gene polymorphisms and lung cancer risk among smokers. Oncol Lett. 2012; 3:1301–06.<https://doi.org/10.3892/ol.2012.665>

86. Rotunno M, Lam TK, Vogt A, Bertazzi PA, Lubin JH, Caporaso NE, Landi MT. GSTM1 and GSTT1 copy numbers and mRNA expression in lung cancer. Mol Carcinog. 2012 (Suppl 1); 51:E142–50.<https://doi.org/10.1002/mc.21890>

87. Zupa A, Sgambato A, Bianchino G, Improta G, Grieco V, LA Torre G, Campisi B, Traficante A, Aieta M, Cittadini A. GSTM1 and NAT2 polymorphisms and colon, lung and bladder cancer risk: a case-control study. Anticancer Res. 2009; 29:1709–14.

88. Sgambato A, Campisi B, Zupa A, Bochicchio A, Romano G, Tartarone A, Galasso R, Traficante A, Cittadini A. Glutathione S-transferase (GST) polymorphisms as risk factors for cancer in a highly homogeneous population from southern Italy. Anticancer Res. 2002; 22:3647–52.

89. Ruano-Ravina A, Pereyra MF, Castro MT, Pérez-Ríos M, Abal-Arca J, Barros-Dios JM. Genetic susceptibility, residential radon, and lung cancer in a radon prone area. J Thorac Oncol. 2014; 9:1073–80.<https://doi.org/10.1097/JTO.0000000000000205>

90. López-Cima MF, Alvarez-Avellón SM, Pascual T, Fernández-Somoano A, Tardón A. Genetic polymorphisms in CYP1A1, GSTM1, GSTP1 and GSTT1 metabolic genes and risk of lung cancer in Asturias. BMC Cancer. 2012; 12:433.<https://doi.org/10.1186/1471-2407-12-433>

91. Gervasini G, San Jose C, Carrillo JA, Benitez J, Cabanillas A. GST polymorphisms interact with dietary factors to modulate lung cancer risk: study in a high-incidence area. Nutr Cancer. 2010; 62:750–58.<https://doi.org/10.1080/01635581003605946>

92. Ruano-Ravina A, Figueiras A, Loidi L, Barros-Dios JM. GSTM1 and GSTT1 polymorphisms, tobacco and risk of lung cancer: a case-control study from Galicia, Spain. Anticancer Res. 2003; 23:4333–37.

93. To-Figueras J, Gené M, Gómez-Catalán J, Piqué E, Borrego N, Carrasco JL, Ramón J, Corbella J. Genetic polymorphism of glutathione S-transferase P1 gene and lung cancer risk. Cancer Causes Control. 1999; 10:65–70.<https://doi.org/10.1023/A:1008811824890>

94. To-Figueras J, Gené M, Gómez-Catalán J, Galán MC, Fuentes M, Ramón JM, Rodamilans M, Huguet E, Corbella J. Glutathione S-transferase M1 (GSTM1) and T1 (GSTT1) polymorphisms and lung cancer risk among Northwestern Mediterraneans. Carcinogenesis. 1997; 18:1529–33.<https://doi.org/10.1093/carcin/18.8.1529>

95. To-Figueras J, Gene M, Gomez-Catalan J, Galan C, Firvida J, Fuentes M, Rodamilans M, Huguet E, Estape J, Corbella J. Glutathione-S-Transferase M1 and codon 72 p53 polymorphisms in a northwestern Mediterranean population and their relation to lung cancer susceptibility. Cancer Epidemiol Biomarkers Prev. 1996; 5:337–42.

96. Mota P, Silva HC, Soares MJ, Pego A, Loureiro M, Cordeiro CR, Regateiro FJ. Genetic polymorphisms of phase I and phase II metabolic enzymes as modulators of lung cancer susceptibility. J Cancer Res Clin Oncol. 2015; 141:851–60.<https://doi.org/10.1007/s00432-014-1868-z>

97. Moreira A, Martins G, Monteiro MJ, Alves M, Dias J, da Costa JD, Melo MJ, Matias D, Costa A, Cristóvão M, Rueff J, Monteiro C. Glutathione S-transferase mu polymorphism and susceptibility to lung cancer in the Portuguese population. Teratog Carcinog Mutagen. 1996; 16:269–74. [https://doi.org/10.1002/(SICI)1520-6866(1996)16:5<269::AID-TCM3>3.0.CO;2-G](https://doi.org/10.1002/(SICI)1520-6866(1996)16:5%3C269::AID-TCM3%3E3.0.CO;2-G)

98. Cote ML, Yoo W, Wenzlaff AS, Prysak GM, Santer SK, Claeys GB, Van Dyke AL, Land SJ, Schwartz AG. Tobacco and estrogen metabolic polymorphisms and risk of non-small cell lung cancer in women. Carcinogenesis. 2009; 30:626–35.<https://doi.org/10.1093/carcin/bgp033>

99. Lam TK, Ruczinski I, Helzlsouer K, Shugart YY, Li KE, Clipp S, Strickland PT, Alberg AJ. Copy number variants of GSTM1 and GSTT1 in relation to lung cancer risk in a prospective cohort study. Ann Epidemiol. 2009; 19:546–52.<https://doi.org/10.1016/j.annepidem.2009.03.003>

100. Cote ML, Kardia SL, Wenzlaff AS, Land SJ, Schwartz AG. Combinations of glutathione S-transferase genotypes and risk of early-onset lung cancer in Caucasians and African Americans: a population-based study. Carcinogenesis. 2005; 26:811–19.<https://doi.org/10.1093/carcin/bgi023>

101. Wenzlaff AS, Cote ML, Bock CH, Land SJ, Schwartz AG. GSTM1, GSTT1 and GSTP1 polymorphisms, environmental tobacco smoke exposure and risk of lung cancer among never smokers: a population-based study. Carcinogenesis. 2005; 26:395–401.<https://doi.org/10.1093/carcin/bgh326>

102. Wang LI, Giovannucci EL, Hunter D, Neuberg D, Su L, Christiani DC. Dietary intake of Cruciferous vegetables, Glutathione S-transferase (GST) polymorphisms and lung cancer risk in a Caucasian population. Cancer Causes Control. 2004; 15:977–85.<https://doi.org/10.1007/s10552-004-1093-1>

103. Cajas-Salazar N, Sierra-Torres CH, Salama SA, Zwischenberger JB, Au WW. Combined effect of MPO, GSTM1 and GSTT1 polymorphisms on chromosome aberrations and lung cancer risk. Int J Hyg Environ Health. 2003; 206:473–83.<https://doi.org/10.1078/1438-4639-00251>

104. Taioli E, Gaspari L, Benhamou S, Boffetta P, Brockmoller J, Butkiewicz D, Cascorbi I, Clapper ML, Dolzan V, Haugen A, Hirvonen A, Husgafvel-Pursiainen K, Kalina I, et al. Polymorphisms in CYP1A1, GSTM1, GSTT1 and lung cancer below the age of 45 years. Int J Epidemiol. 2003; 32:60–63.<https://doi.org/10.1093/ije/dyg001>

105. Tsai YY, McGlynn KA, Hu Y, Cassidy AB, Arnold J, Engstrom PF, Buetow KH. Genetic susceptibility and dietary patterns in lung cancer. Lung Cancer. 2003; 41:269–81. Erratum in: Lung Cancer. 2004; 44:271.

106. Miller DP, Liu G, De Vivo I, Lynch TJ, Wain JC, Su L, Christiani DC. Combinations of the variant genotypes of GSTP1, GSTM1, and p53 are associated with an increased lung cancer risk. Cancer Res. 2002; 62:2819–23.

107. Perera FP, Mooney LA, Stampfer M, Phillips DH, Bell DA, Rundle A, Cho S, Tsai WY, Ma J, Blackwood A, Tang D, and Physicians’ Health Cohort Study. Associations between carcinogen-DNA damage, glutathione S-transferase genotypes, and risk of lung cancer in the prospective Physicians’ Health Cohort Study. Carcinogenesis. 2002; 23:1641–46.<https://doi.org/10.1093/carcin/23.10.1641>

108. Liu G, Miller DP, Zhou W, Thurston SW, Fan R, Xu LL, Lynch TJ, Wain JC, Su L, Christiani DC. Differential association of the codon 72 p53 and GSTM1 polymorphisms on histological subtype of non-small cell lung carcinoma. Cancer Res. 2001; 61:8718–22.

109. Spitz MR, Duphorne CM, Detry MA, Pillow PC, Amos CI, Lei L, de Andrade M, Gu X, Hong WK, Wu X. Dietary intake of isothiocyanates: evidence of a joint effect with glutathione S-transferase polymorphisms in lung cancer risk. Cancer Epidemiol Biomarkers Prev. 2000; 9:1017–20.

110. Garcia-Closas M, Kelsey KT, Wiencke JK, Xu X, Wain JC, Christiani DC. A case-control study of cytochrome P450 1A1, glutathione S-transferase M1, cigarette smoking and lung cancer susceptibility (Massachusetts, United States). Cancer Causes Control. 1997; 8:544–53.<https://doi.org/10.1023/A:1018481910663>

111. el-Zein R, Zwischenberger JB, Wood TG, Abdel-Rahman SZ, Brekelbaum C, Au WW. Combined genetic polymorphism and risk for development of lung cancer. Mutat Res. 1997; 381:189–200.<https://doi.org/10.1016/S0027-5107(97)00166-8>

112. London SJ, Daly AK, Leathart JB, Navidi WC, Idle JR. Lung cancer risk in relation to the CYP2C9*1/CYP2C9*2 genetic polymorphism among African-Americans and Caucasians in Los Angeles County, California. Pharmacogenetics. 1996; 6:527–33.<https://doi.org/10.1097/00008571-199612000-00006>

113. Cheng TJ, Christiani DC, Wiencke JK, Wain JC, Xu X, Kelsey KT. Comparison of sister chromatid exchange frequency in peripheral lymphocytes in lung cancer cases and controls. Mutat Res. 1995; 348:75–82.<https://doi.org/10.1016/0165-7992(95)00049-6>

114. London SJ, Daly AK, Cooper J, Navidi WC, Carpenter CL, Idle JR. Polymorphism of glutathione S-transferase M1 and lung cancer risk among African-Americans and Caucasians in Los Angeles County, California. J Natl Cancer Inst. 1995; 87:1246–53.<https://doi.org/10.1093/jnci/87.16.1246>

115. Carpenter CL, Yu MC, London SJ. Dietary isothiocyanates, glutathione S-transferase M1 (GSTM1), and lung cancer risk in African Americans and Caucasians from Los Angeles County, California. Nutr Cancer. 2009; 61:492–99.<https://doi.org/10.1080/01635580902752270>

116. Yang P, Bamlet WR, Ebbert JO, Taylor WR, de Andrade M. Glutathione pathway genes and lung cancer risk in young and old populations. Carcinogenesis. 2004; 25:1935–44.<https://doi.org/10.1093/carcin/bgh203>

117. Nazar-Stewart V, Vaughan TL, Stapleton P, Van Loo J, Nicol-Blades B, Eaton DL. A population-based study of glutathione S-transferase M1, T1 and P1 genotypes and risk for lung cancer. Lung Cancer. 2003; 40:247–58.<https://doi.org/10.1016/S0169-5002(03)00076-X>

118. Dresler CM, Fratelli C, Babb J, Everley L, Evans AA, Clapper ML. Gender differences in genetic susceptibility for lung cancer. Lung Cancer. 2000; 30:153–60.<https://doi.org/10.1016/S0169-5002(00)00163-X>

119. Le Marchand L, Sivaraman L, Pierce L, Seifried A, Lum A, Wilkens LR, Lau AF. Associations of CYP1A1, GSTM1, and CYP2E1 polymorphisms with lung cancer suggest cell type specificities to tobacco carcinogens. Cancer Res. 1998; 58:4858–63.

120. Kelsey KT, Spitz MR, Zuo ZF, Wiencke JK. Polymorphisms in the glutathione S-transferase class mu and theta genes interact and increase susceptibility to lung cancer in minority populations (Texas, United States). Cancer Causes Control. 1997; 8:554–59.<https://doi.org/10.1023/A:1018434027502>

121. Ford JG, Li Y, O’Sullivan MM, Demopoulos R, Garte S, Taioli E, Brandt-Rauf PW. Glutathione S-transferase M1 polymorphism and lung cancer risk in African-Americans. Carcinogenesis. 2000; 21:1971–75.<https://doi.org/10.1093/carcin/21.11.1971>

122. Cabral RE, Caldeira-de-Araujo A, Cabral-Neto JB, Costa Carvalho MG. Analysis of GSTM1 and GSTT1 polymorphisms in circulating plasma DNA of lung cancer patients. Mol Cell Biochem. 2010; 338:263–69.<https://doi.org/10.1007/s11010-009-0360-6>

123. Honma HN, De Capitani EM, Perroud MW Jr, Barbeiro AS, Toro IF, Costa DB, Lima CS, Zambon L. Influence of p53 codon 72 exon 4, GSTM1, GSTT1 and GSTP1*B polymorphisms in lung cancer risk in a Brazilian population. Lung Cancer. 2008; 61:152–62.<https://doi.org/10.1016/j.lungcan.2007.12.014>

124. Adonis M, Martínez V, Marín P, Berrios D, Gil L. Smoking habit and genetic factors associated with lung cancer in a population highly exposed to arsenic. Toxicol Lett. 2005; 159:32–37.<https://doi.org/10.1016/j.toxlet.2005.04.007>

125. Quiñones L, Lucas D, Godoy J, Cáceres D, Berthou F, Varela N, Lee K, Acevedo C, Martínez L, Aguilera AM, Gil L. CYP1A1, CYP2E1 and GSTM1 genetic polymorphisms. The effect of single and combined genotypes on lung cancer susceptibility in Chilean people. Cancer Lett. 2001; 174:35–44.<https://doi.org/10.1016/S0304-3835(01)00686-3>

126. Kiyohara C, Horiuchi T, Takayama K, Nakanishi Y. Genetic polymorphisms involved in carcinogen metabolism and DNA repair and lung cancer risk in a Japanese population. J Thorac Oncol. 2012; 7:954–62.<https://doi.org/10.1097/JTO.0b013e31824de30f>

127. Kohno T, Kunitoh H, Mimaki S, Shiraishi K, Kuchiba A, Yamamoto S, Yokota J. Contribution of the TP53, OGG1, CHRNA3, and HLA-DQA1 genes to the risk for lung squamous cell carcinoma. J Thorac Oncol. 2011; 6:813–17.<https://doi.org/10.1097/JTO.0b013e3181ee80ef>

128. Tamaki Y, Arai T, Sugimura H, Sasaki T, Honda M, Muroi Y, Matsubara Y, Kanno S, Ishikawa M, Hirasawa N, Hiratsuka M. Association between cancer risk and drug-metabolizing enzyme gene (CYP2A6, CYP2A13, CYP4B1, SULT1A1, GSTM1, and GSTT1) polymorphisms in cases of lung cancer in Japan. Drug Metab Pharmacokinet. 2011; 26:516–22.<https://doi.org/10.2133/dmpk.DMPK-11-RG-046>

129. Osawa Y, Osawa KK, Miyaishi A, Higuchi M, Tsutou A, Matsumura S, Tabuchi Y, Tsubota N, Takahashi J. NAT2 and CYP1A2 polymorphisms and lung cancer risk in relation to smoking status. Asian Pac J Cancer Prev. 2007; 8:103–08.

130. Kiyohara C, Wakai K, Mikami H, Sido K, Ando M, Ohno Y. Risk modification by CYP1A1 and GSTM1 polymorphisms in the association of environmental tobacco smoke and lung cancer: a case-control study in Japanese nonsmoking women. Int J Cancer. 2003; 107:139–44.<https://doi.org/10.1002/ijc.11355>

131. Oyama T, Kagawa N, Kim YD, Matsumoto A, Isse T, Kawamoto T. Lung cancer andCYP1A1 orGSTM1 polymorphisms. Environ Health Prev Med. 2003; 7:230–34.<https://doi.org/10.1007/BF02908880>

132. Sunaga N, Kohno T, Yanagitani N, Sugimura H, Kunitoh H, Tamura T, Takei Y, Tsuchiya S, Saito R, Yokota J. Contribution of the NQO1 and GSTT1 polymorphisms to lung adenocarcinoma susceptibility. Cancer Epidemiol Biomarkers Prev. 2002; 11:730–38.

133. Kiyohara C, Yamamura KI, Nakanishi Y, Takayama K, Hara N. Polymorphism in GSTM1, GSTT1, and GSTP1 and Susceptibility to Lung Cancer in a Japanese Population. Asian Pac J Cancer Prev. 2000; 1:293–98.

134. Kihara M, Kihara M, Noda K. Lung cancer risk of the GSTM1 null genotype is enhanced in the presence of the GSTP1 mutated genotype in male Japanese smokers. Cancer Lett. 1999; 137:53–60.<https://doi.org/10.1016/S0304-3835(98)00337-1>

135. Katoh T. [Application of molecular biology to occupational health field--the frequency of gene polymorphism of cytochrome P450 1A1 and glutathione S-transferase M1 in patients with lung, oral and urothelial cancer]. [Article in Japanese]. J UOEH. 1995; 17:271–78.<https://doi.org/10.7888/juoeh.17.271>

136. Kihara M, Kihara M, Noda K. Risk of smoking for squamous and small cell carcinomas of the lung modulated by combinations of CYP1A1 and GSTM1 gene polymorphisms in a Japanese population. Carcinogenesis. 1995; 16:2331–36.<https://doi.org/10.1093/carcin/16.10.2331>

137. Kihara M, Noda K, Kihara M. Distribution of GSTM1 null genotype in relation to gender, age and smoking status in Japanese lung cancer patients. Pharmacogenetics. 1995; 5:S74–79.<https://doi.org/10.1097/00008571-199512001-00005>

138. Kihara M, Kihara M, Noda K. Lung cancer risk of GSTM1 null genotype is dependent on the extent of tobacco smoke exposure. Carcinogenesis. 1994; 15:415–18.<https://doi.org/10.1093/carcin/15.2.415>

139. Nakachi K, Imai K, Hayashi S, Kawajiri K. Polymorphisms of the *CYP1A1* and glutathione S-transferase genes associated with susceptibility to lung cancer in relation to cigarette dose in a Japanese population. Cancer Res. 1993; 53:2994–99.

140. Piao JM, Shin MH, Kim HN, Cui LH, Song HR, Kweon SS, Choi JS, Kim YC, Oh IJ, Kim KS. Glutathione-S-transferase (GSTM1, GSTT1) null phenotypes and risk of lung cancer in a Korean population. Asian Pac J Cancer Prev. 2013; 14:7165–69.<https://doi.org/10.7314/APJCP.2013.14.12.7165>

141. Yang M, Choi Y, Hwangbo B, Lee JS. Combined effects of genetic polymorphisms in six selected genes on lung cancer susceptibility. Lung Cancer. 2007; 57:135–42.<https://doi.org/10.1016/j.lungcan.2007.03.005>

142. Lee KM, Kang D, Lee SJ, Park SK, Lee KH, Choi JY, Kim SU, Choi H, Choi SH, Kim YW, Hong YC, Cho SH. Interactive effect of genetic polymorphism of glutathione S-transferase M1 and smoking on squamous cell lung cancer risk in Korea. Oncol Rep. 2006; 16:1035–39.

143. Hong YS, Chang JH, Kwon OJ, Ham YA, Choi JH. Polymorphism of the CYP1A1 and glutathione-S-transferase gene in Korean lung cancer patients. Exp Mol Med. 1998; 30:192–98.<https://doi.org/10.1038/emm.1998.28>

144. Chou YC, Wu MS, Wu CC, Yang T, Chu CM, Lai CH, Hsieh CY, You SL, Chen CJ, Sun CA. Total Urinary Isothiocyanates, Glutathione S-Transferase M1 Genotypes, and Lung Cancer Risk: A Preliminary Nested Case-Control Study in Taiwan. J Med Sci. 2005; 25:21–26.

145. Cheng YW, Chen CY, Lin P, Huang KH, Lin TS, Wu MH, Lee H. DNA adduct level in lung tissue may act as a risk biomarker of lung cancer. Eur J Cancer. 2000; 36:1381–88.<https://doi.org/10.1016/S0959-8049(00)00131-3>

146. Jiang XY, Chang FH, Bai TY, Lv XL, Wang MJ. Susceptibility of lung cancer with polymorphisms of CYP1A1, GSTM1, GSTM3, GSTT1 and GSTP1 genotypes in the population of Inner Mongolia region. Asian Pac J Cancer Prev. 2014; 15:5207–14.<https://doi.org/10.7314/APJCP.2014.15.13.5207>

147. Pan C, Zhu G, Yan Z, Zhou Y, Liu Z. Glutathione S-transferase T1 and M1 polymorphisms are associated with lung cancer risk in a gender-specific manner. Oncol Res Treat. 2014; 37:164–69.<https://doi.org/10.1159/000361083>

148. Zhang H, Wu X, Xiao Y, Chen M, Li Z, Wei X, Tang K. Genetic polymorphisms of glutathione S-transferase M1 and T1, and evaluation of oxidative stress in patients with non-small cell lung cancer. Eur J Med Res. 2014; 19:67.<https://doi.org/10.1186/s40001-014-0067-3>

149. Chen CM, Jin YT, Xu HY, Zhang CY, Zhang H, Zhang WM, Tan C, Sun XY. [Effects of CYP1A1 and GSTM1 gene polymorphisms and BPDE-DNA adducts on lung cancer]. [Article in Chinese]. Zhonghua Yi Xue Yi Chuan Xue Za Zhi. 2012; 29:23–27.<https://doi.org/10.3760/cma.j.issn.1003-9406.2012.01.007>

150. Liu D, Wang F, Wang Q, Guo X, Xu H, Wang W, Zhang L. Association of glutathione S-transferase M1 polymorphisms and lung cancer risk in a Chinese population. Clin Chim Acta. 2012; 414:188–90.<https://doi.org/10.1016/j.cca.2012.09.016>

151. Li W, Yue W, Zhang L, Zhao X, Ma L, Yang X, Zhang C, Wang Y, Gu M. Polymorphisms in GSTM1, CYP1A1, CYP2E1, and CYP2D6 are associated with susceptibility and chemotherapy response in non-small-cell lung cancer patients. Hai. 2012; 190:91–98.<https://doi.org/10.1007/s00408-011-9338-8>

152. Fowke JH, Gao YT, Chow WH, Cai Q, Shu XO, Li HL, Ji BT, Rothman N, Yang G, Chung FL, Zheng W. Urinary isothiocyanate levels and lung cancer risk among non-smoking women: a prospective investigation. Lung Cancer. 2011; 73:18–24.<https://doi.org/10.1016/j.lungcan.2010.10.024>

153. Jin Y, Zhang C, Xu H, Xue S, Wang Y, Hou Y, Kong Y, Xu Y. Combined effects of serum trace metals and polymorphisms of CYP1A1 or GSTM1 on non-small cell lung cancer: a hospital based case-control study in China. Cancer Epidemiol. 2011; 35:182–7. <https://doi.org/10.1016/j.canep.2010.06.004>

154. Jin Y, Xu H, Zhang C, Kong Y, Hou Y, Xu Y, Xue S. Combined effects of cigarette smoking, gene polymorphisms and methylations of tumor suppressor genes on non small cell lung cancer: a hospital-based case-control study in China. BMC Cancer. 2010; 10:422.<https://doi.org/10.1186/1471-2407-10-422>

155. Zheng D, Hua F, Mei C, Wan H, Zhou Q. [Association between GSTM1 genetic polymorphism and lung cancer risk by SYBR green I real-time PCR assay]. [Article in Chinese]. Zhongguo Fei Ai Za Zhi. 2010; 13:506–10.<https://doi.org/10.3779/j.issn.1009-3419.2010.05.23>

156. Zhu XX, Hu CP, Gu QH. [CYP1A1 polymorphisms, lack of glutathione S-transferase M1 (GSTM1), cooking oil fumes and lung cancer risk in non-smoking women]. Zhonghua Jie He He Hu Xi Za Zhi. [Article in Chinese]. 2010; 33:817–22.<https://doi.org/10.3760/cma.j.issn.1001-0939.2010.11.004>

157. Chang F, Hu T, Wang G. [Relationship between CYP1A1 and GSTM1 genetic polymorphisms and lung cancer susceptibility in population of Inner Mongolia]. [Article in Chinese]. Zhongguo Fei Ai Za Zhi. 2006; 9:413–17.<https://doi.org/10.3779/j.issn.1009-3419.2006.05.04>

158. Chen HC, Cao YF, Hu WX, Liu XF, Liu QX, Zhang J, Liu J. Genetic polymorphisms of phase II metabolic enzymes and lung cancer susceptibility in a population of Central South China. Dis Markers. 2006; 22:141–52.<https://doi.org/10.1155/2006/436497>

159. Qian BY, Han HW, Gu F, He M, Li HX, Song FJ, Lay L, Dong SF, Chen KS. [Case-control study genetic polymorphism in CYP1A1 and GSTM1 and smoking and susceptibility to lung cancer]. [Article in Chinese]. Chin J Clin Oncol. 2006; 9:500–02.

160. Chan EC, Lam SY, Fu KH, Kwong YL. Polymorphisms of the GSTM1, GSTP1, MPO, XRCC1, and NQO1 genes in Chinese patients with non-small cell lung cancers: relationship with aberrant promoter methylation of the CDKN2A and RARB genes. Cancer Genet Cytogenet. 2005; 162:10–20.<https://doi.org/10.1016/j.cancergencyto.2005.03.008>

161. Li D, Zhou Q, Yuan T, Guo Z, Zhu W, Wang Y, Chen X, Feng Z, Che G. [Study on the association between genetic polymorphism of CYP2E1, GSTM1 and susceptibility of lung cancer]. [Article in Chinese]. Zhongguo Fei Ai Za Zhi. 2005; 8:14–19.

162. Li Y, Tang XY, Ma XT, Chen J. Glutathione S-transferase M1 polymorphisms and susceptibility to lung cancer. J Med Drug Forum. 2005; 16:10–12.

163. Wang N, Zhuang DG, Wu YM. [Research on relationship between GSTM1 gene deletion and lung cancer genetic susceptibility]. [Article in Chinese]. Acta Henan Univ Sci Technol. 2005; 23:7–8.

164. Ye WY, Chen SD, Chen Q. [Interaction between serum selenium level and polymorphism of GSTM1 in lung cancer]. [Article in Chinese]. Ying Yang Xue Bao. 2005; 27:17–20.

165. Zeng M, Chen SD, Xie CM, Wang BG, Feng YX, Chen MX, Li BH. Case control study on relationship between lung cancer and its susceptibility marker. Chin J Publ Health. 2005; 21:771–74.

166. Cao Y, Chen H, Liu X. Study on the relationship between the genetic polymorphisms of GSTM1 and GSTT1 genes and lung cancer susceptibility in the population of Hunan province of China. Life Science Res. 2004; 8:126–32.

167. Chen M, Chen S, Wang B. A case-control study of the impact of glutathione S-transferase M1 on the risk of lung cancer. Chin Tumor. 2004; 13:686–88.

168. Dong C, Yang Q, Wang M, Dong Q. A study on the relationship between polymorphism of CYP1A1, lack of GSTM1, and susceptibility to lung cancer. J Occup Environ Med. 2004; 21:440–42.

169. Gu Y, Zhang S, Lai B, Wang H, Zhan X. [Relationship between genetic polymorphism of metabolizing enzymes and lung cancer susceptibility]. [Article in Chinese]. Zhongguo Fei Ai Za Zhi. 2004; 7:112–17.<https://doi.org/10.3779/j.issn.1009-3419.2004.02.08>

170. Liang GY, Pu YP, Yin LH. [Studies of the genes related to lung cancer susceptibility in Nanjing Han population, China]. [Article in Chinese]. Yi Chuan. 2004; 26:584–88.

171. Luo CL, Chen Q, Cao WF, Chen SD. Combined analysis of polymorphism of GSTM1 and mutagens and p53 gene in the patients with lung cancer. Chin J Clin Oncol. 2004; 31:1218–20.

172. Wang BG, Chen SD, Zhou WP, Zeng M, Li ZB, Cai XL, Wang DQ. [A case control study on the impact of CYP450 MSPI and GST-M1 polymorphisms on the risk of lung cancer]. [Article in Chinese]. Zhonghua Zhong Liu Za Zhi. 2004; 26:93–97.

173. Yang XR, Wacholder S, Xu Z, Dean M, Clark V, Gold B, Brown LM, Stone BJ, Fraumeni JF Jr, Caporaso NE. CYP1A1 and GSTM1 polymorphisms in relation to lung cancer risk in Chinese women. Cancer Lett. 2004; 214:197–204.<https://doi.org/10.1016/j.canlet.2004.06.040>

174. Ye W, Chen Q, Chen S. Study on relationship between GSTM1polymorphism, diet factors, and lung cancer. Chin J Publ Health. 2004; 20:1120–21.

175. Chan-Yeung M, Tan-Un KC, Ip MS, Tsang KW, Ho SP, Ho JC, Chan H, Lam WK. Lung cancer susceptibility and polymorphisms of glutathione-S-transferase genes in Hong Kong. Lung Cancer. 2004; 45:155–60.<https://doi.org/10.1016/j.lungcan.2004.01.016>

176. Chen L, Sun H, Xu Y. Study on the allele frequency of GSTM1 gene in normal Han population in Wannan area and the relationship between GSTM1 genotype and the risk of lung cancer. J Wannan Medical College. 2003; 22:13–16.

177. Wang J, Deng Y, Li L, Kuriki K, Ding J, Pan X, Zhuge X, Jiang J, Luo C, Lin P, Tokudome S. Association of GSTM1, CYP1A1 and CYP2E1 genetic polymorphisms with susceptibility to lung adenocarcinoma: a case-control study in Chinese population. Cancer Sci. 2003; 94:448–52.<https://doi.org/10.1111/j.1349-7006.2003.tb01463.x>

178. Wang J, Deng Y, Cheng J, Ding J, Tokudome S. GST genetic polymorphisms and lung adenocarcinoma susceptibility in a Chinese population. Cancer Lett. 2003; 201:185–93.<https://doi.org/10.1016/S0304-3835(03)00480-4>

179. Xian X, Chen S, Wang B. The relationship between polymorphism of GSTM1 and susceptibility to lung cancer. Pract Prev Med. 2003; 10:635–37.

180. Chan Y, Wang X, Wang XY, Liang ZQ. [A study of genetic polymorphism of GSTM1 gene in normal population and lung cancer population in Yunnan]. [Article in Chinese]. Acta Yunnan Normal Univ. 2002; 22:52–54.

181. Lu W, Xing D, Qi J, Tan W, Miao X, Lin D. Genetic polymorphism in myeloperoxidase but not GSTM1 is associated with risk of lung squamous cell carcinoma in a Chinese population. Int J Cancer. 2002; 102:275–79.<https://doi.org/10.1002/ijc.10712>

182. Shi Y, Zhou X, Zhou Y. Analysis of CYP2E1, GSTM1 genetic polymorphisms in relation to human lung cancer and esophagealcarcinoma. J Huazhong Uni Sci Tech. 2002; 31:14–17.

183. Zhang L, Wang X, Hao X, Liu Z. Relationship between susceptibility to lung cancer and genetic polymorphism in P4501A1, GSTM1. J Clin Oncol. 2002; 29:536–40.

184. Chen S, Xue K, Xu L, Ma G, Wu J. Polymorphisms of the CYP1A1 and GSTM1 genes in relation to individual susceptibility to lung carcinoma in Chinese population. Mutat Res. 2001; 458:41–47.

185. Xue K, Xu L, Chen S, Ma G, Wu J. [Polymorphisms of the CYP1A1 and GSTM1 genes and their combined effects on individual susceptibility to lung cancer in a Chinese population]. [Article in Chinese]. Zhonghua Yi Xue Yi Chuan Xue Za Zhi. 2001; 18:125–27.

186. Lan Q, He X, Costa DJ, Tian L, Rothman N, Hu G, Mumford JL. Indoor coal combustion emissions, GSTM1 and GSTT1 genotypes, and lung cancer risk: a case-control study in Xuan Wei, China. Cancer Epidemiol Biomarkers Prev. 2000; 9:605–08.

187. London SJ, Yuan JM, Chung FL, Gao YT, Coetzee GA, Ross RK, Yu MC. Isothiocyanates, glutathione S-transferase M1 and T1 polymorphisms, and lung-cancer risk: a prospective study of men in Shanghai, China. Lancet. 2000; 356:724–29.<https://doi.org/10.1016/S0140-6736(00)02631-3>

188. Chen SQ, Xu L, Ma GJ, Wu JZ, Xue KX. Identification of genetic polymorphism of CYP1A1 and GSTM1 and lung cancer patients by using allele-specific PCR and multiplex differential PCR. Carcinog Teratog Mutag. 1999; 11:119–21.

189. Gao Y, Zhang Q. Polymorphisms of the GSTM1 and CYP2D6 genes associated with susceptibility to lung cancer in Chinese. Mutat Res. 1999; 444:441–49.<https://doi.org/10.1016/S1383-5718(99)00092-3>

190. Lan Q, He X, Costa D, Tian W. [Glutathione S-transferase GSTM1 and GSTT1 genotypes and susceptibility to lung cancer]. [Article in Chinese]. Wei Sheng Yan Jiu. 1999; 28:9–11.

191. Persson I, Johansson I, Lou YC, Yue QY, Duan LS, Bertilsson L, Ingelman-Sundberg M. Genetic polymorphism of xenobiotic metabolizing enzymes among Chinese lung cancer patients. Int J Cancer. 1999; 81:325–29. [https://doi.org/10.1002/(SICI)1097-0215(19990505)81:3<325::AID-IJC2>3.0.CO;2-S](https://doi.org/10.1002/(SICI)1097-0215(19990505)81:3%3C325::AID-IJC2%3E3.0.CO;2-S)

192. Gao J, Ren C, Zhang Q. [CYP2D6 and GSTM1 genetic polymorphism and lung cancer susceptibility]. [Article in Chinese]. Zhonghua Zhong Liu Za Zhi. 1998; 20:185–86.

193. Sun GF, Shimojo N, Pi JB, Lee S, Kumagai Y. Gene deficiency of glutathione S-transferase mu isoform associated with susceptibility to lung cancer in a Chinese population. Cancer Lett. 1997; 113:169–72.<https://doi.org/10.1016/S0304-3835(97)04612-0>

194. Ge H, Lam WK, Lee J, Wong MP, Yew WW, Lung ML. Analysis of L-myc and GSTM1 genotypes in Chinese non-small cell lung carcinoma patients. Lung Cancer. 1996; 15:355–66.<https://doi.org/10.1016/0169-5002(95)00598-6>

195. Klinchid J, Chewaskulyoung B, Saeteng S, Lertprasertsuke N, Kasinrerk W, Cressey R. Effect of combined genetic polymorphisms on lung cancer risk in northern Thai women. Cancer Genet Cytogenet. 2009; 195:143–49.<https://doi.org/10.1016/j.cancergencyto.2009.08.011>

196. Pisani P, Srivatanakul P, Randerson-Moor J, Vipasrinimit S, Lalitwongsa S, Unpunyo P, Bashir S, Bishop DT. GSTM1 and CYP1A1 polymorphisms, tobacco, air pollution, and lung cancer: a study in rural Thailand. Cancer Epidemiol Biomarkers Prev. 2006; 15:667–74.<https://doi.org/10.1158/1055-9965.EPI-05-0667>

197. Zhao B, Seow A, Lee EJ, Poh WT, Teh M, Eng P, Wang YT, Tan WC, Yu MC, Lee HP. Dietary isothiocyanates, glutathione S-transferase -M1, -T1 polymorphisms and lung cancer risk among Chinese women in Singapore. Cancer Epidemiol Biomarkers Prev. 2001; 10:1063–67.

198. Girdhar Y, Singh N, Behera D, Sharma S. Combinations of the Variant Genotypes of CYP1A1, GSTM1 and GSTT1 are Associated with an Increased Lung Cancer Risk in North Indian Population: a Case-Control Study. Pathol Oncol Res. 2016; 22:647–52.<https://doi.org/10.1007/s12253-016-0058-5>

199. Peddireddy V, Badabagni SP, Gundimeda SD, Mamidipudi V, Penagaluru PR, Mundluru HP. Association of CYP1A1, GSTM1 and GSTT1 gene polymorphisms with risk of non-small cell lung cancer in Andhra Pradesh region of South India. Eur J Med Res. 2016; 21:17.<https://doi.org/10.1186/s40001-016-0209-x>

200. Sharma N, Singh A, Singh N, Behera D, Sharma S. Genetic polymorphisms in GSTM1, GSTT1 and GSTP1 genes and risk of lung cancer in a North Indian population. Cancer Epidemiol. 2015; 39:947–55.<https://doi.org/10.1016/j.canep.2015.10.014>

201. Ihsan R, Chauhan PS, Mishra AK, Singh LC, Sharma JD, Zomawia E, Verma Y, Kapur S, Saxena S. Copy number polymorphism of glutathione-S-transferase genes (GSTM1 & GSTT1) in susceptibility to lung cancer in a high-risk population from north-east India. Indian J Med Res. 2014; 139:720–29.

202. Phukan RK, Saikia BJ, Borah PK, Zomawia E, Sekhon GS, Mahanta J. Role of household exposure, dietary habits and glutathione S-Transferases M1, T1 polymorphisms in susceptibility to lung cancer among women in Mizoram India. Asian Pac J Cancer Prev. 2014; 15:3253–60.<https://doi.org/10.7314/APJCP.2014.15.7.3253>

203. Shukla RK, Tilak AR, Kumar C, Kant S, Kumar A, Mittal B, Bhattacharya S. Associations of CYP1A1, GSTM1 and GSTT1 polymorphisms with lung cancer susceptibility in a Northern Indian population. Asian Pac J Cancer Prev. 2013; 14:3345–49.<https://doi.org/10.7314/APJCP.2013.14.5.3345>

204. Ihsan R, Chauhan PS, Mishra AK, Yadav DS, Kaushal M, Sharma JD, Zomawia E, Verma Y, Kapur S, Saxena S. Multiple analytical approaches reveal distinct gene-environment interactions in smokers and non smokers in lung cancer. PLoS One. 2011; 6:e29431.<https://doi.org/10.1371/journal.pone.0029431>

205. Kumar M, Agarwal SK, Goel SK. Lung cancer risk in north Indian population: role of genetic polymorphisms and smoking. Mol Cell Biochem. 2009; 322:73–79.<https://doi.org/10.1007/s11010-008-9941-z>

206. Shah PP, Singh AP, Singh M, Mathur N, Mishra BN, Pant MC, Parmar D. Association of functionally important polymorphisms in cytochrome P4501B1 with lung cancer. Mutat Res. 2008; 643:4–10.<https://doi.org/10.1016/j.mrfmmm.2008.05.001>

207. Sobti RC, Kaur P, Kaur S, Janmeja AK, Jindal SK, Kishan J, Raimondi S. Combined effect of GSTM1, GSTT1 and GSTP1 polymorphisms on histological subtypes of lung cancer. Biomarkers. 2008; 13:282–95.<https://doi.org/10.1080/13547500701843437>

208. Sreeja L, Syamala V, Hariharan S, Syamala VS, Raveendran PB, Sivanandan CD, Madhavan J, Ankathil R. Glutathione S-transferase M1, T1 and P1 polymorphisms: susceptibility and outcome in lung cancer patients. J Exp Ther Oncol. 2008; 7:73–85.

209. Sreeja L, Syamala V, Hariharan S, Madhavan J, Devan SC, Ankathil R. Possible risk modification by CYP1A1, GSTM1 and GSTT1 gene polymorphisms in lung cancer susceptibility in a South Indian population. J Hum Genet. 2005; 50:618–27.<https://doi.org/10.1007/s10038-005-0303-3>

210. Masood N, Taseer B, Yasmin A. Association of GSTM1 and GSTT1 deletion with lung cancer development in Pakistani population. J Cancer Res Ther. 2016; 12:731–34.<https://doi.org/10.4103/0973-1482.148667>
